# Regulation of Bim in Health and Disease

**DOI:** 10.18632/oncotarget.5492

**Published:** 2015-09-05

**Authors:** Ronit Vogt Sionov, Spiros A. Vlahopoulos, Zvi Granot

**Affiliations:** ^1^ Department of Developmental Biology and Cancer Research, Institute for Medical Research Israel Canada, Hebrew University, Hadassah Medical School, Jerusalem, Israel; ^2^ First Department of Pediatrics, University of Athens, Horemeio Research Laboratory, Thivon and Levadias, Goudi, Athens, Greece

**Keywords:** Bim, apoptosis, cancer, autoimmunity, neurodegenerative diseases

## Abstract

The BH3-only Bim protein is a major determinant for initiating the intrinsic apoptotic pathway under both physiological and pathophysiological conditions. Tight regulation of its expression and activity at the transcriptional, translational and post-translational levels together with the induction of alternatively spliced isoforms with different pro-apoptotic potential, ensure timely activation of Bim. Under physiological conditions, Bim is essential for shaping immune responses where its absence promotes autoimmunity, while too early Bim induction eliminates cytotoxic T cells prematurely, resulting in chronic inflammation and tumor progression. Enhanced Bim induction in neurons causes neurodegenerative disorders including Alzheimer's, Parkinson's and Huntington's diseases. Moreover, type I diabetes is promoted by genetically predisposed elevation of Bim in β-cells. On the contrary, cancer cells have developed mechanisms that suppress Bim expression necessary for tumor progression and metastasis. This review focuses on the intricate network regulating Bim activity and its involvement in physiological and pathophysiological processes.

## INTRODUCTION

Understanding the dynamic nature of apoptotic regulators is very important in the design of intervention schemes for a number of debilitating conditions. It has become apparent that many pharmaceutical agents target only the initiating step in an intracellular signal cascade. While this is important, because an initiating step gets subsequently amplified, in many diseases a downstream step malfunctions, making most available drug treatments futile.

Over the past decade, Bim has emerged as an essential pro-apoptotic protein for initiating the intrinsic apoptotic pathway under many physiological and pathophysiological conditions. The complex network regulating its expression and activity has made it possible to manipulate cell death at several nodal points. A fine balance in the intracellular expression levels of Bim and its regulatory proteins is crucial for properly regulating apoptosis. This review will take us into a journey through the fascinating world of Bim.

## 1. THE STRUCTURE OF BIM

### 1.1. General Aspects of Bim

The BH3-only proteins participate in vital biological processes, and their absence contributes to autoimmunity and neoplasia [1, 2]. Bim is a Bcl-2 homology 3 (BH3)-only protein that was discovered by O'Connor et al. [3] in 1998, while screening for proteins binding the anti-apoptotic Bcl-2 protein, giving raise to its name *B*cl-2 *i*nteracting *m*ediator of cell death. In the same year, Hsu et al. [4] discovered the same gene using Mcl-1 as a bait and termed the gene Bcl-2 related ovarian death agonist (BOD). Its official gene name is now Bcl-2-like 11 (Bcl-2L11/apoptosis facilitator). The Bim gene is conserved in diverse mammalian species [4]. Bim function seems also to be conserved in non-mammalian vertebrates. A zebrafish ortholog of mammalian Bim was found to be the most toxic product of the zebrafish BH3-only genes examined, sharing this characteristic with the mammalian Bim gene [5].

Bim proteins are expressed in a wide variety of tissues including brain, heart, kidney, liver, lung, ovary, testis, spleen, thymus and trachea, but are most prominently expressed by cells of hematopoietic origin [6]. In the yeast two-hybrid protein-protein interaction assay, Bim interacts with the anti-apoptotic Bcl-2 proteins Mcl-1, Bcl-2, Bcl-xL, Bcl-w, Bfl-1 and Epstein-Barr virus (EBV) BHRF-1, but not with the pro-apoptotic Bcl-2 proteins Bad, Bak, Bok and Bax [4]. In mammalian cells, Bim can engage all anti-apoptotic proteins of the Bcl-2 superfamily, making it an efficient killer [1]. The BH3 domain becomes inserted into a hydrophobic groove of the pro-survival relatives [1]. The hydrophobic C-terminal part of Bim (MVILRLLRYIVRLVW) targets the protein to intracellular membranes [3]. In addition, Bim can directly interact with Bax and Bak, leading to mitochondrial outer membrane permeabilization [7-11]. Genetic analyses by Merino et al. [11] showed that only the Bim BH3 domain, but not other BH3 sequences inserted into the Bim protein, could rescue the leukocyte accumulation and autoimmune phenotype of Bim knockout (KO) mice, emphasizing the unique killing potency of Bim BH3. Thus, Bim may promote apoptosis by both acting as a death agonist and survival antagonist [8]. Other pro-apoptotic members of the BH3-only family include Bad, Bik, Bmf, Bid, Hrk, Noxa and Puma [12]. Bim often acts in concert with these pro-apoptotic proteins, which may explain why apoptosis is only partly compromised in Bim KO mice, as seen for glucocorticoid-induced apoptosis of thymocytes [2]. In contrast to normal cells, apoptosis of cancer cells often shows a complete dependence on Bim [13, 14]. The BH3-only pro-apoptotic proteins act upstream of the multi-domain pro-apoptotic proteins Bak, Bax and Bok, which have three BH domains (BH1, BH2 and BH3). Double knockout of Bax and Bak often leads to complete resistance to apoptosis mediated by the intrinsic apoptotic pathway [15].

The multiple defects in Bcl-2^−/−^ mice can be prevented by loss of Bim, including the severe lymphopenia [16]. Even the loss of only one Bim allele was sufficient for the correction of the disorders, suggesting that the Bim levels set the threshold for initiation of apoptosis in several tissues [16].

### 1.2. Bim Isoforms

**Figure 1:**
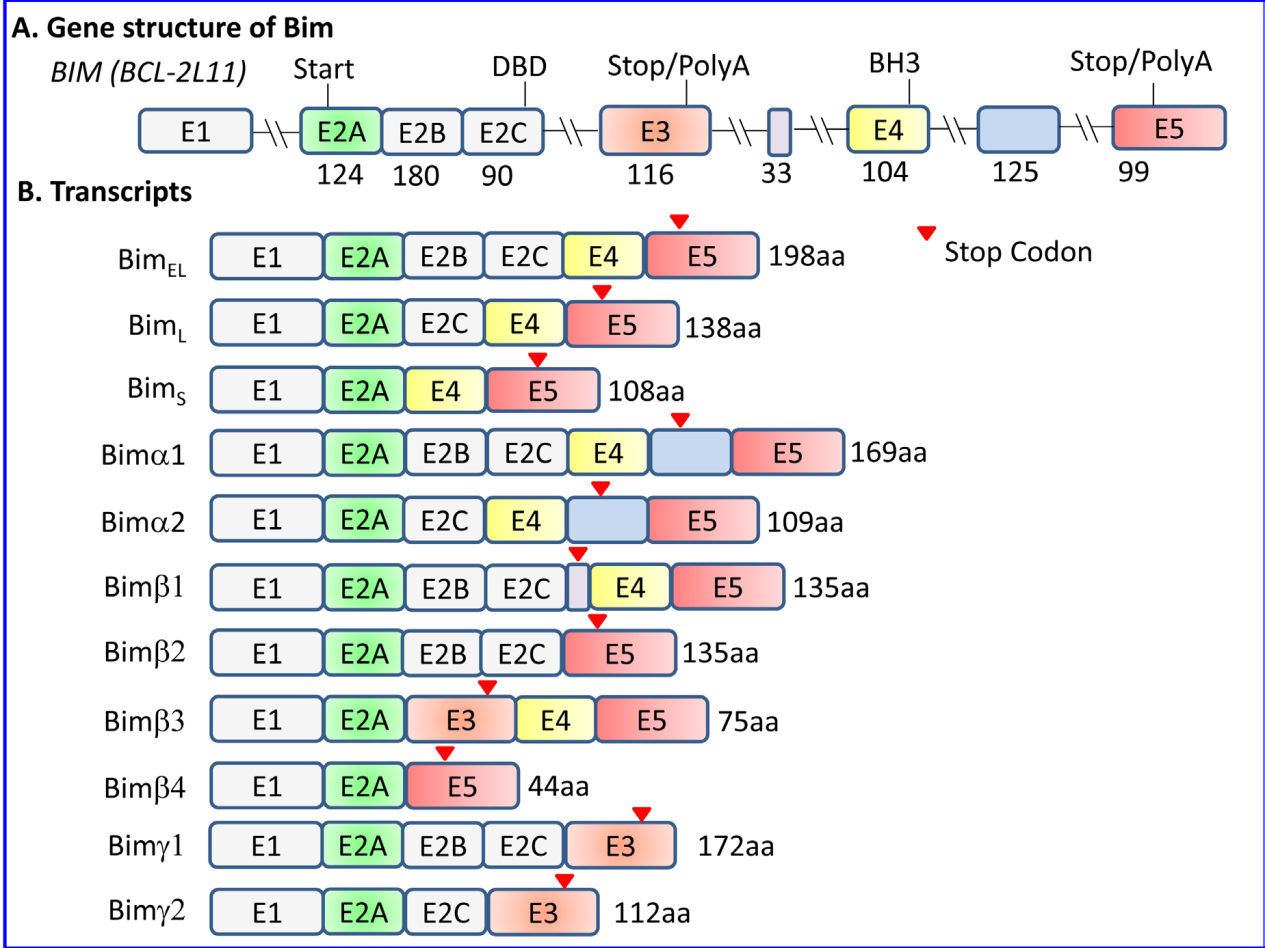
The gene structure of Bim and its major isoform transcripts **A.** Presentation of the Bim (Bcl-2L11) gene structure according to the nomenclature of U et al. [28]. There has been much confusion in the literature concerning the nomenclature of the exon numbers where some research groups have denoted exons E2A, E2B and E2C as exons 2, 3 and 4 respectively, and exons E3, E4 and E5 as exons 5, 6 and 7, respectively. As there is no intron between E2A and E2B, and E2B and E2C, these regions are part of one exon, where intraexonal alternative splicing gives raise to the inclusion or exclusion of the E2B and/or E2C region. DBD - Dynein-binding domain. The BH3 domain is located in exon E4. The numbers beneath the exons refer to the amount of coding nucleotides in each exon. **B.** Presentation of various Bim isoform transcripts formed by alternative splicing. Bim_EL_, Bim_L_ and Bim_S_ are the major classical isoforms, but also other isoforms have been identified as described in Section 1.2. Bimα1-2 and Bimβ1-4 were described by U et al. [28], while Bimγ by Ng et al. [31] that was coined Bimγ2 by Anczukow et al. [229]. The latter research group characterized an additional Bimγ isoform (Bimγ1) that also retained the E2B exon [229]. Although Ng et al. [31] claim for a mutual exclusion of exon E3 and E4, the Bimβ3 isoform described by U et al. [28] does contain both exons. As the E3 exon contains a stop codon, its inclusion leads to a truncated protein lacking the pro-apoptotic BH3 domain. The Bim-ABCD, Bim-ACD and Bim-AD described by Marani et al. [9] corresponds to Bimα1, Bimα2 and Bimα3. Bimα3 resembles Bimα2, but lacks E2C.

The gene locus of Bcl2L11 is located at chromosome 2q13 in human. The gene has 6 exons, and undergoes alternative splicing to form at least 18 different isoforms transcribed from mRNA harboring 3-6 exons of which 2-4 are coding exons (Figure 1 and Supplementary Table 1). The three major alternative transcription variants of Bim described in mouse give raise to Bim_EL_ (Extra Long) (196 aa; MW∼23,000 Da), Bim_L_ (Long) (140 aa; MW∼19,000 Da) and Bim_S_ (Short) (110 aa; MW∼15,000 Da), where Bim_EL_ is the most abundant [3, 17]. In human, these isoforms correspond to Bim_EL_ (Bcl2L11 isoform 1) (198 aa; 22.1 kDa), Bim_L_ (Bcl2L11 isoform 6) (138 aa; 15.9 kDa) and Bim_S_ (Bcl2L11 isoform 11) (108 aa; 12.7 kDa). Bim_L_ and Bim_S_ are formed by alternative splicing within exon 2. Bim_EL_ is the most abundant form in thymocytes and T cells, whereas Bim_S_ is almost undetectable [18, 19]. All three isoforms are induced by glucocorticoids in thymocytes [20] and pre-B acute lymphoblastic leukemia [21].

All of the major isoforms contain a consensus BH3 domain of 9 amino acids (LRRIGDEFN) forming an amphipathic α helix, but lack other BH domains (BH1, BH2 and BH4) found in channel-forming Bcl-2 family proteins [3, 4]. Bim_EL_ and Bim_L_, but not Bim_S_, possess the dynein light (L) chain-binding domain (DBD) encoded by exon E2C. Bim_EL_ and Bim_L_ are released from microtubules in response to apoptotic stimuli, making them available for interaction with anti-apoptotic proteins such as Bcl-2 [22].

All 3 isoforms induce apoptosis; the shortest being the most potent [3]. The latter can be explained by the ability of Bim_S_ to directly bind the pro-apoptotic Bax protein [9, 23, 24], together with the absence of sequestration to the cytoskeleton [22] and post-translational regulation [17, 25]. There are some cell-specific effects. For instance, enforced expression of Bim_L_ or Bim_S_ readily induced apoptosis in Baf-3 and 293 cells, while Bim_S_, but not Bim_L_, killed glioma cells [26, 27].

Upregulated expression of Bim_S_ in epithelial cells was followed by its rapid mitochondrial translocation and mitochondrial insertion in the absence of any detectable binding to anti-apoptotic Bcl-2 proteins [24]. This caused mitochondrial recruitment and activation of Bax and apoptosis. The mitochondrial targeting, but not binding to Bcl-2 or Mcl-1, was required for apoptosis induction by Bim_S_ [24]. In yeast, Bim_S_ enhanced the killing activity of Bax in the absence of anti-apoptotic Bcl-2 proteins [24].

Additional isoforms have been described in human with differential pro-apoptotic activity [9, 28-30] (Figure 1 and Supplementary Table 1). U et al. [28] described six isoforms of human Bim (Bimα1,α2, and β1-4) that lacked the C-terminal hydrophobic region. Among these isoforms, only α1 and α2 contained a BH3 domain and were pro-apoptotic, although less potent than the classical isoforms [28]. Marani et al. [9] described six isoforms that they termed BimAC, BimABC, BimAD, BimACD, BimA and BimABCD, all having a truncated C-terminus, ending with either GIFE or LEK instead of the classical hydrophobic region. They showed that the small BimAD isoform that encodes for an 80 aa protein and harbors the BH3 domain, is pro-apoptotic [9]. Chen et al. [29] defined the BimAD as Bimα3, and showed that this isoform is pro-apoptotic to a similar extent as Bimα2, but is less potent than Bim_S_ and Bim_L_. Some of the isoforms described by Marani et al. [9] are similar to those described by U et al. [28] (Supplementary Table 1). Liu et al. [30] described an isoform termed Bimγ (112 aa; MW∼15,000), that is generated as a result of a retention of a 126-bp of intron 2 of the Bim gene. As the sequence of intron 2 contains a stop codon, the Bimγ isoform contains only the E2A, E2B, E2C coding exons and the intron 2 until reaching the pre-mature TGA stop codon. It contains the dynein-binding domain (DKSTQT) presented in E2C, but lacks the classical BH3 domain encoded by exon 4 [30]. A BH3-like domain (LEDIGD instead of LRRIGD) is formed from the intron sequence that may be responsible for its pro-apoptotic and growth-inhibitory function [30].

### 1.3. Bim Polymorphism

Ng et al. [31] observed that a common intronic 2903bp-deletion polymorphism within intron 2 in the Bim gene switched Bim splicing from exon 4 to exon 3 leading to the preferential expression of Bimγ. This deletion polymorphism occurs naturally in 12.9% of East-Asian individuals and is observed in some individuals with chronic myeloid leukemia (CML) or epidermal growth factor receptor (EGFR)-mutated non-small-cell lung cancer (NSCLC) that conferred resistance to tyrosine kinase inhibitors such as gefitinib [31, 32]. Of note, the resistance could be overcome by the BH3-mimetic drug ABT-737 [31] or by combining gefitinib with the histone deacetylase inhibitor vorinostat that increased the splicing to exon 4, leading to augmented expression of the pro-apoptotic BH3-containing Bim [32].

The intronic deletion polymorphism may explain the heterogeneic response of cancer patients to tyrosine kinase inhibitors [31]. The progression free survival following EGFR tyrosine kinase inhibitor treatment of NSCLC was significantly shorter in patients with Bim polymorphism (6.6 months) than those with wild-type Bim (11.9 months) [31]. However, others couldn't find an association of Bim deletion polymorphism and intrinsic resistance to tyrosine kinase inhibitors [33].

Another study showed that acute lymphoblastic leukemia patients harboring the single nucleotide BimC29201T (rs724710) polymorphism in exon 4 had shorter overall survival [34]. Overall survival was even shorter in patients with both Bim polymorphism and Mcl-1 gene polymorphism (G486T) [34]. The single nucleotide polymorphism C>T in Bim affected the inclusion of exon 3 and seems to contribute to drug resistance [34].

### 1.4. Bim Knockout (KO) Mice

A significant number of *bim*-null mice die *in utero* before E9.5, suggesting that Bim plays a role in development [2]. These mice accumulate lymphoid, myeloid and plasma cells and develop autoimmune kidney disease due to impaired apoptosis [2]. Bim-deficient mice have a higher number of B cells, CD4 and CD8 single-positive T cells, macrophages and granulocytes in the periphery. Expansion of the B cell population is associated with accumulation of serum immunoglobulins [2]. The abnormal increase in serum levels of IgM and IgG could be due to protection of plasma cells from endoplasmic reticulum (ER) stress-induced apoptosis, which in lymphoid and certain other cell types requires Bim [35]. The sensitivity of pre-B cells and autoreactive B cells to apoptotic stimuli was low in Bim^−/−^ mice [2, 36]. With age, Bim KO mice develop splenomegaly, lymphadenopathy, and hyper-gammaglobulinemia [2]. Although Bim is required for deletion of autoreactive thymocytes, Bim-deficient mice do not succumb to extensive organ-specific autoimmune disease, which may be due to an increase in T regulatory (T_reg_) cells [37-40], impaired T cell activation [41] and reduced apoptotic sensitivity of the Bim-deficient target cells (See Section 4). Bim KO mice also showed gastric abnormality due to excessive accumulation of cells in the gastric epithelial layer [42].

In T cells, loss of Bim increases T cell production and function in interleukin-7 receptor (IL-7R; CD127)-deficient mice [43]. Bim deficiency can partially rescue B cell development in mice deficient for the crucial B cell growth factor IL-7 [44]. Bim deficiency attenuates hematopoietic cell death in the fetal liver of Bcl-x-deficient mice, and could rescue testicular degeneration in Bcl-x^+/−^ mice [45]. However, Bim deficiency couldn't prevent neuronal cell death in Bcl-x-deficient mice [45]. Loss of Bim renders lymphocytes refractory to paclitaxel (Taxol), ionomycin and cytokine deprivation, and partial resistance to glucocorticoids [2]. Death of thymocytes recognizing superantigens (Mtv-9 and *Staphylococcus* enterotoxin B) and male antigen HY was almost completely blocked in *Bim^−/−^* mice [46]. Deletion of antigen-activated T cells during the shutdown of immune responses is also hindered in these mice [47].

Further studies show that Puma co-operates with Bim in apoptosis induction during lymphocyte development [48]. The absence of Puma or Bim renders thymocytes and mature lymphocytes refractory to varying degrees to death induced *in vitro* by growth factor withdrawal, DNA damage or glucocorticoids [49]. Bim^−/−^/Puma^−/−^ mice develop multiple postnatal defects that are not observed in the single knockout mice [48]. Hyperplasia of lymphatic organs is comparable with that observed in mice overexpressing Bcl-2 in all hematopoietic cells, exceeding the hyperplasia observed in Bim^−/−^ mice [48]. Mice deficient for both Puma and Bim spontaneously developed autoimmunity in multiple organs, and their T cells could transfer organ-specific autoimmunity [50]. Puma- and Bim-double-deficient mice showed accumulation of mature, single-positive thymocytes, suggesting that an additional defect in thymic deletion is the basis for the autoimmune disease [50]. Transgenic mouse models of thymocyte deletion by peripheral neoantigens confirmed that the loss of Bim and Puma allowed increased numbers of autoreactive thymocytes to escape deletion [50].

Deficiency of Bim, but not Puma, partially rescued B cell development in the absence of IL-7 [51]. The numbers of both sIgM-negative and sIgM-positive B cells were markedly increased in the bone marrow of recipients lacking IL-7 upon reconstitution with Bim-deficient hematopoietic progenitors, compared with their control or Puma-deficient counterparts [51]. The augmentation of B cell lymphopoiesis in the absence of Bim was reflected in the mature peripheral compartment by an increase in both the number of immature and mature B cells in the spleen and in the circulating IgM levels [51].

Mice lacking both Bim and Bik showed similar hematopoietic alterations as Bim-deficient mice [52]. However, the double Bim/Bik KO male mice were infertile with reduced testicular cellularity and no spermatozoa [52]. The testis of young Bim/Bik double KO male mice had increased numbers of spermatogonia and spermatocytes, suggesting that spermatogenesis fails due to overwhelming amounts of supporting Sertoli cells [52].

## 2. ROLE OF BIM IN APOPTOSIS

### 2.1. Indirect and Direct Apoptosis Induction by Bim

**Figure 2:**
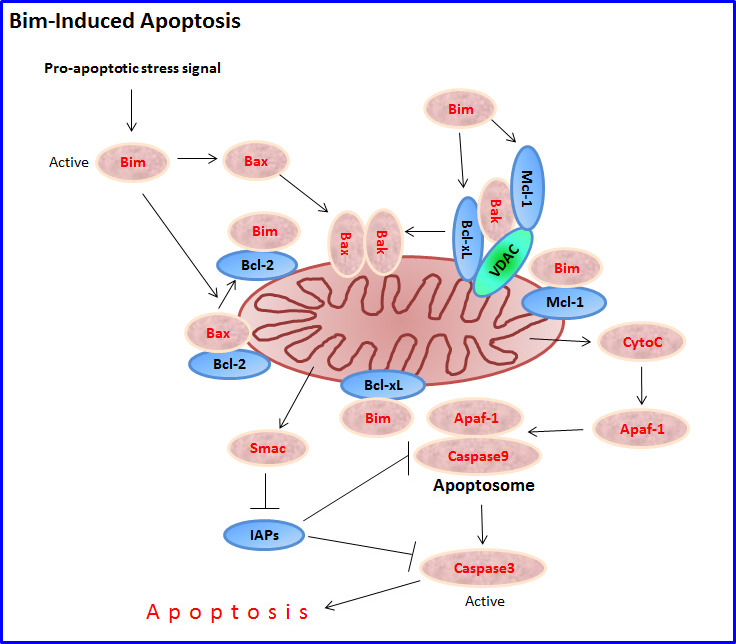
Bim-induced apoptosis Following exposure to a pro-apoptotic stimulus, a sudden intracellular rise in free activated Bim molecules (e.g., by increased transcription and/or translation, increased alternative splicing in favor of Bim_S_, and/or release of Bim from sequestered intracellular storages as a result of phosphorylation) initiates the intrinsic mitochondrial apoptotic pathway. Bim induces apoptosis by directly activating Bax and Bak, or indirectly by interacting with the anti-apoptotic proteins Bcl-2, Bcl-xL and Mcl-1, leading to the release and mitochondrial transfer of Bax and Bak. Under normal conditions, Bak is hold in check by Mcl-1, VDAC2 and Bcl-xL. Bax/Bak oligomerization in the mitochondrial outer membrane results in dissipation of the mitochondrial outer membrane potential (Δψ_m_) and release of the apoptogenic proteins cytochrome C (CytoC), Smac/DIABLO and HtrA2 into the cytosol. Cytochrome C activates Apoptotic protease activating factor 1 (Apaf-1) that facilitates the formation of the apoptosome where caspase 9 is activated to initiate the apoptotic cascade concluded with the activation of caspase 3. Smac/DIABLO antagonizes the anti-apoptotic function of Inhibitors of apoptosis protein (IAPs) such as XIAP, cIAP1 and cIAP2, thereby enhancing apoptosis induction by cytochrome C.

Bim has been implicated in the regulation of intrinsic cell death induced by a large number of stimuli, including growth factor or cytokine deprivation, calcium flux, ligation of antigen receptors on T and B cells, loss of adhesion (anoikis), glucocorticoids, microtubule perturbation and tyrosine kinase inhibitors (Tables 1, 2, 3, 4, 5, 6, 7). It has been shown to be critical for apoptosis in B and T lymphocytes, macrophages and granulocytes [53]. The pioneer studies by O'Connor et al. [3] and Hsu et al. [4], showed that overexpression of Bim in Chinese hamster ovary (CHO) cells or 293T human embryonic kidney cells led to apoptosis that could be prevented by the baculoviral caspase inhibitor P35, and the anti-apoptotic Bcl-2, Bcl-xL and Bcl-w proteins. However, the more distant viral homologues adenovirus E1B19K and Epstein-Barr virus BHRF-1 were unable to prevent the pro-apoptotic effect of Bim [3]. The BH3 domain is essential for its pro-apoptotic function [3, 4]. A mutant Bim protein lacking the BH3 domain did not interact with Bcl-2, Bcl-xL or Bcl-w [3]. Although all three Bim isoforms in mouse bind Bcl-2, Bim_S_ antagonizes Bcl-2 more effectively than Bim_L_, while Bim_EL_ was the least potent [3]. Besides neutralizing the anti-apoptotic proteins, Bim promotes apoptosis by binding to Bax leading to a conformation change in Bax that leads to its activation [8, 9] (Figure 2).

**Table 1:** Bim Function in The Immune System

Apoptotic Stimulus	Remarks	References
Negative selection of self-reactive thymocytes	• Thymocytes lacking Bim are refractory to apoptosis induced by TCR-CD3 stimulation. • Bim is required for apoptosis of CD4^+^CD8^+^ thymocytes induced by high-affinity antigens. • Bim is involved in clonal deletion of thymocytes recognizing tissue-restricted antigens (TRAs), but not superantigen-mediated apoptosis. • Autoreactive NODBim^−/−^ thymocytes receiving strong TCR signals that would normally delete them, escape apoptosis and are diverted into T_reg_ cells. • Bim is essential for deletion of CD4^+^CD8^−^CD24^−^ thymocytes in response to TCR ligation.	[40, 46, 401, 402, 404-406, 416]
Activated T cell death	• Bim is a key regulator of T cell apoptosis during the contraction phase of CD8^+^ T cell response. • Vβ8^+^ T cells from Bim-deficient mice are resistant to staphylococcus enterotoxin B-induced T cell death. • While the Bim levels did not change after exposure to staphylococcal enterotoxin B, the Bcl-2 levels decreased. • Shutdown of an acute T cell response to herpes simplex virus involved Bim. • Bim deficiency increases antigen-specific CD8^+^ T cell responses during viral infection.	[47, 395, 398, 399]
B7-H1 (PD-L1)-induced apoptosis of effector T cells	• B7-H1 (PD-L1) engagement with its receptor PD-1 promotes apoptosis of effector T cells through upregulation of Bim. • More memory CD8^+^ T cells were generated in B7-H1-deficient mice following immunization.	[409]
Elimination of poorly functional Th1 responder cells	• Rescued Bim^−/−^ CD4^+^ memory T cells showed deficient effector functions, poor sensitivity to antigen and an inability to respond to secondary challenge. • Bim mediates T cell death in the absence of appropriate TCR-driven activation and differentiation. • Bim shapes the CD4^+^ memory T cell repertoire by eliminating Th1 effector cells with suboptimal function, thereby ensuring the emergence of highly functional CD4^+^ memory cells.	[408]
Regulation of T memory cells	• The absence of Bim increased the effector CD8^+^ T cell population with more memory potential. • Survival of memory T cells depends on TRAF1-mediated Bim downregulation. • The absence of Bim-mediated death of lymphocytic choriomeningitis virus-specific CD4^+^ and CD8^+^ T cells *in vivo* leads to increased differentiation, even of CD127^lo^ T cells, into memory T cells.	[411, 422, 471]
Regulation of T regulatory cells	• In the absence of Bim, T regulatory cells accumulate rapidly, accounting for >25% of the CD4^+^ T cells in aged mice. • Rapid peripheral T regulatory cell turnover depends on Bim. • Induced regulatory T cells show decreased Bcl-2 expression and increased Bim expression and were more prone to apoptosis. • Rag^−/−^ hosts repopulated with Bim^−/−^ conventional CD4^+^ T cells resulted in a larger induction of regulatory T cells than mice given wild-type conventional CD4^+^ cells. • Bim deficient mice showed increased numbers of CD25^low^Foxp3^+^ cells in the thymus and peripheral lymph tissue. The CD25^low^Foxp3^+^ CD4^+^ cells were anergic and had weaker regulatory function than CD25^high^Foxp3^+^ CD4^+^ T cells from the same mice.	[37-39, 394, 413]
B cell antigen receptor (BCR) stimulation-induced apoptosis	• B lymphocytes lacking Bim are refractory to apoptosis induced by BCR ligation. • The loss of Bim also inhibited deletion of autoreactive B cells *in vivo* in a B cell tolerance model. • Siglecs induce tolerance to cell surface antigens by Bim-dependent deletion of antigen-reactive B cells. • Cross-linked anti-μ antibodies that trigger apoptosis of human B lymphocytes, induce ERK-dependent downregulation of Bim_EL_, with simultaneous upregulation of the Bim_L_ and Bim_S_ isoforms.	[36, 83, 429]
Elimination of autoreactive B cells	• Bim is involved in the elimination of autoreactive and anergic B cells.	[36, 426]
Superantigen-mediated B cell death	• The microbial virulence factor protein A of Staphylococcus aureus interact with evolutionarily conserved BCR-binding sites to induce a form of Bim-dependent activation-associated B cell death.	[427]
Apoptosis of low-affinity-expressing B cells.	• After immunization, Bim-deficient mice showed persistence of memory B cells lacking affinity-enhancing mutations in their immunoglobulin genes and antibody-forming cells secreting low-affinity antibodies.	[392]
Spontaneous and stress-induced apoptosis of granulocytes	• Bim deficiency renders granulocytes resistant to cytokine withdrawal and cytotoxic drugs such as etoposide and paclitaxel. • GM-CSF treatment temporarily blocks apoptosis by inducing Mcl-1 with rapid turnover and Bim, which limits GM-CSF-mediated prolonged survival of neutrophils.	[357, 433, 434]
Phagocytosis-induced apoptosis of macrophages	• Phagocytosis and intracellular killing of bacteria lead to apoptosis of macrophages that involve TLR-, p38- and JNK-dependent upregulation of Bim. • Phagocytosis-induced apoptosis was strongly reduced in Bim^−/−^ macrophages.	[430]
Spontaneous cell death of dendritic cells	• Bim-deficient dendritic cells showed decreased spontaneous cell death and induced more robust T cell activation.	[432]
Antigen-specific NK cell contraction	• Antigen-specific NK cell contraction after mouse cytomegalovirus infection depends on Bim.	[436]
Mast cell apoptosis	• Bim is induced together with Bcl-xL upon IgE receptor activation of mast cells.	[358]
Osteoclast apoptosis	• Liver X receptor activation leads to osteoclast apoptosis through Bim upregulation.	[645]
Cytokine deprivation	• Cytokine withdrawal leads to activation of FKHR-L1 in lymphocytes, which is responsible for the upregulation of Bim expression. •Early hematopoietic progenitor cells (Sca-1^+^, c-Kit^+^, Lin^−^) undergo rapid apoptosis in the absence of cytokines concomitant with Bim induction. • IL-3 signaling leads to phosphorylation of Bim_EL_ and its consequent degradation in hematopoietic stem cells. • IL-3 downregulates Bim through the Ras/MAPK and PI3K/Akt pathways. • M-CSF deprivation of osteoclasts leads to Bim-dependent apoptosis. • Bim deficiency prevented cytokine withdrawal-induced mast cell apoptosis. • PGE_2_ increases mast cell death during cytokine deprivation by augmenting Bim expression.	[26, 122, 358, 360, 438, 646]
IL-21-induced apoptosis of CLL	• IL-21 induces apoptosis of chronic lymphoblastic leukemia (CLL) by activating the STAT-1 pathway and Bim induction. • IL-21 increased the cytotoxic effect of fludarabine and rituximab on CLL.	[190]

**Table 2:** Bim Function in The Nerve System

Apoptotic Stimulus	Remarks	References
Sympathetic neuronal death induced by NGF deprivation	• Nerve growth factor (NGF) withdrawal induced Bim_EL_ expression and apoptosis by a mechanism dependent on c-Jun. • Bim deletion protected against neuronal apoptosis. • NGF promoted MEK/MAPK-mediated phosphorylation of Bim_EL_ at Ser109 and Thr110, thereby suppressing its activity.	[125, 311, 355]
β-Amyloid-induced neuronal apoptosis	• Cdk4 and its downstream effector B-myb are required for β-amyloid-dependent Bim induction and death in cultured neurons. • β-Amyloid activates Mst-1-mediated nuclear translocation of FoxO3 that is important for Bim imduction.	[352, 388]
Thrombin-induced apoptosis of cultured cerebral cortical neurons	• Cyclin D1, Cdk4 and Bim were shown to be involved in thrombin-induced apoptosis of cultured cerebral cortical neurons.	[383]
Ischemic neuronal cell death	• Ischemia leads to neuronal cell death mediated by Notch- and NFκB-dependent Bim upregulation. • Bim-deficient mice showed decreased parenchymal loss in the hippocampal area following neonatal hypoxia-ischemia.	[351, 447]
Parkinson's disease	• Dysfunction of mitochondrial complex I leads to degeneration of dopaminergic neurons through JNK-dependent activation of Bim.	[445]
Huntington's disease	• Overexpression of mutant Huntingtin protein leads to increased Bim_EL_ expression, and knockdown of Bim prevents apoptosis mediated by mutant Huntingtin.	[180]
p75 Neurotrophin receptor (p75NTR)	• Overexpression of p75NTR induced JNK-dependent phosphorylation of Bim_EL_ at Ser65 in primary cerebellar granule neurons, which resulted in apoptosis.	[324]

**Table 3:** Bim in Stress-Induced Apoptosis

Apoptotic Stimulus	Remarks	References
Anoikis	• Bim is strongly induced after epithelial cell detachment and downregulation of Bim inhibited anoikis. • Overexpression of EGFR maintains ERK activity following detachment, thus preventing Bim induction and anoikis. • Overexpression of HER2 in breast cancer increases HIF-1α expression and activates ERK and Akt, resulting in reduced Bim expression and prevention of anoikis. • Pokemon renders liver cells resistant to anoikis via suppression of Bim transcription. • Extracellular matrix metalloproteinase inducer (CD147) confers resistance of breast cancer cells to anoikis through inhibition of Bim. • Mesothelin and PINCH-1 promote ERK-mediated Bim degradation and anchorage-independent growth.	[210, 224, 488, 489, 490, 492, 495-497]
cAMP-induced apoptosis	• 8-CPT-cAMP treatment of T lymphoma cells induced Bim expression and apoptosis. • Cyclic AMP-dependent protein kinase A regulates apoptosis by stabilizing Bim. • Bim is required for cAMP-mediated apoptosis of double positive thymocytes.	[341-343]
AICAR	• 5-Aminoimidazole-4-carboxamide riboside or acadesine (AICAR) induces apoptosis in chronic lymphocytic leukemia through a mechanism dependent on Bim and Noxa, but independent of AMPKα1, p53 and ATM.	[647]
ER stress	• Deficiency of Bim/Puma impedes ER-stress-induced Bax/Bak activation and apoptosis. • ER stress activates Bim by CHOP-C/EBPα-mediated transactivation and phosphatase 2A-mediated dephosphorylation that leads to Bim stabilization.	[35, 69]
Coagulation factor Fxa-induced apoptosis	• The coagulation factor Fxa that converts prothrombin into active thrombin, induces apoptosis of epithelial tumor cells through a CREB- and Bim-dependent mechanism.	[386]
Heat shock-induced apoptosis	• Bim-deficient cells are resistant to heat shock-induced cell death to a similar extent as Bax^−/−^Bak^−/−^ cells.	[648]
Ionomycin	•CD4^+^8^+^ thymocytes from Bim KO mice were resistant to ionomycin.	[2]
Serum-withdrawal-induced apoptosis	• Serum withdrawal induces Bim expression and apoptosis of CCl39 fibroblasts. Both processes were prevented by thrombin through activation of the Raf-MEK-ERK1/2 and PI3K pathways. A protease-activated receptor 1 (PAR1) agonist peptide also protected cells from serum-withdrawal-induced apoptosis.	[376]
UV-induced apoptosis	• Knockdown of Bim inhibited UV-induced cell death of COS-7 cells.	[60]

**Table 4:** Bim in Diabetes

Apoptotic Stimulus	Remarks	References
Diabetes-susceptibility genes	• Diabetes-related GLIS deficiency leads to preferential upregulation of the Bim_S_ isoform. • Diabetes-related PTPN2 deficiency leads to STAT-1-mediated Bim upregulation. • Deficiency of BACH2 leads to downregulation of PTPN2 and Bim upregulation. • Reduced expression of Cathepsin H leads to increased Bim expression.	[183, 233, 454, 455]
Pdx-1- haploinsufficiency	• The increased apoptosis of β-cells in Pdx-1-haploinsufficient mice could be prevented by simultaneous knockdown of Bim.	[456]
IRS2-deficiency	• Bim mediates β-cell death in IRS2-deficient mice.	[457]
Non-obese diabetic (NOD) mice	• Decreased thymic deletion associated with diminished induction of Bim when encountering high-avidity autoantigen.	[452]
Glucose cytotoxicity of insulin-producing β-cells	• Langerhans' islets lacking Bim or Puma were protected from glucose cytotoxicity. • High glucose induces the expression of CHOP that co-operates with FoxO3a to regulate Bim and Puma expression.	[181, 450, 451, 649]
Pro-inflammatory cytokines	• Bim induced in Langerhans' islets by pro-inflammatory cytokines promotes β-cell death.	[183, 188]
Thapsigargin	• Loss of Bim or Puma partially protects Langerhans' islets from the ER stressor thapsigargin.	[450]

**Table 5:** Bim in Liver Pathophysiology

Apoptotic Stimulus	Remarks	References
Acetaminophen-induced liver damage	• Acetaminophen (paracetamol) induced Bim expression in hepatocytes through a JNK-dependent manner. • Bim-deficient mice were protected from acetaminophen-induced liver damage.	[464]
Hepatocyte lipoapoptosis	• Free saturated fatty acids induce lipoapoptosis of hepatocytes through FoxO3a-dependent Bim upregulation and JNK-mediated Bim activation. • Free fatty acids induce the expression of PP2A responsible for FoxO3a activation. • Palmitic acid induces degradation of Keap-1, resulting in JNK-mediated upregulation of Bim.	[466, 467, 469]
Inflammation-induced hepatocyte apoptosis	• Overactivation of the immune system can lead to apoptotic death of hepatocytes that is mediated by TNFα-induced JNK-mediated activation of Bim and caspase 8-mediated activation of Bid.	[468]
Virus-induced hepatitis	• Bim is involved in the elimination of liver-activated virus-specific T cells following HBV/HCV and LCMV infections. • LCMV leads to persistence of cytotoxic T cells that induce hepatocyte apoptosis by a Bim-dependent mechanism. • Bim^−/−^ hepatocytes showed reduced sensitivity to T cell-induced apoptosis. • Bim is upregulated in HBV-specific CD8^+^ T cells from patients with chronic HBV infection, leading to early contraction of the immune response.	[353, 472, 474, 475]

**Table 6:** Bim in the Cancer Context

Apoptotic Stimulus	Remarks	References
Granzyme B	• Bim-deficient 3T9 transformed mouse embryonic fibroblasts are resistant to Granzyme B-induced apoptosis.	[650]
IFNα	• IFNα-induced apoptosis of multiple myeloma cells depends on Jak1 and Bim.	[601]
Melatonin • (N-acetyl-5-methoxy-tryptamine)	• Melatonin induces apoptosis of HepG2 hepatoma cells through FoxO3a-mediated induction of Bim. • Melatonin induces apoptosis of human renal Caki cells by upregulating Bim through induction of SP-1 and E2F1-mediated transcription and inhibition of proteasomal activity.	[318, 320]
c-Myc induced apoptosis	• Bim mediates c-Myc induced apoptosis in solid tumors.	[191]
TGFβ-induced apoptosis	• Bim is the most downstream apoptotic effector of the TGFβ pathway, and its downmodulation abrogates TGFβ-dependent apoptosis in the gastric epithelial cell line SNU16. • TGFβ-induced apoptosis of adenomatous polyposis coli cells is mediated by Bim. • TGFβ1 induces human osteoclast apoptosis by upregulating Bim. • TGFβ induces Bim expression through a mechanism that involves Smad3/4, Runx1/3, p38 and JNK. • Runx1 cooperates with FoxO3a to transcriptionally induce Bim.	[42, 152, 153, 165, 166, 553-555]

**Table 7:** Bim in Drug-Induced Apoptosis

Apoptotic Stimulus	Remarks	References
ALK and c-Met inhibitors	• The ALK inhibitor TAE684 induces apoptosis in lung cancer cells through upregulation of Bim and downregulation of survivin. • The dual ALK and c-Met inhibitor Crizotinib induces apoptosis in c-Met-amplificated lung cancer cells and gastric cancer cells through upregulation of Bim. • When combined with EGFR inhibitors, the dual ALK and c-Met inhibitor CM-118 induced apoptosis of c-Met amplified NSCLC cells through Bim upregulation and Mcl-1 downregulation.	[623, 625-627]
BH3 mimetics (e.g., ABT-737, ABT-263 and ABT-199)	• The apoptosis induction of ovarian cancer by ABT-737 was dependent on Bim expression. • ABT-737-mediated release of Bak couldn't induce apoptosis unless Bim associates with oligomeric Bak to promote its conversion to a membrane-inserted pore. • The pro-apoptotic effect of ABT-737 in CLL depends on sufficient amount of Bcl-2 that tonically sequesters the pro-apoptotic Bim protein. • Lung cancer with EGFR mutation and high Bcl-2 expression could be sensitized to cell death by a combination of erlotinib and ABT-737 that was dependent on Bcl-2 primed with Bim. • Primary B-ALL cells expressing high levels of Bcl-2 exhibited great sensitivity to ABT-263 and ABT-199 • BH3 profiling of lymphoma cells identifies cells dependent on Bcl-2 and predicts sensitivity to ABT-737. • Hepatocyte apoptosis induced by ABT-737 was completely prevented in Bim/Bid double knockout mice.	[78, 309, 515, 572, 577, 585, 651]
Bortezomib (Velcade)	• Bortezomib-induced apoptosis of multiple myeloma cells was prevented upon knockdown of Bim. • Bortezomib sensitized prostate cancer cells to TRAIL-induced apoptosis through a mechanism dependent on Bim.	[485, 507]
Etoposide and Doxorubicin	• Etoposide- and doxorubicin-induced apoptosis of neuroblastoma cells was dependent on FoxO3-mediated Bim expression and ROS production that could be prevented by Bcl-xL.	[57]
Flt3 inhibitors	• The Flt3 inhibitors AG1295 and PKC412 induced apoptosis of acute myeloid leumemia cells that was dependent on FoxO3a-mediated Bim induction and inactivation of the PI3K/Akt pathway.	[652]
Glucocorticoids	• Glucocorticoid-induced apoptosis of thymocytes, T and B acute lymphoblastic leukemia, chronic lymphoblastic leukemia, multiple myeloma and thymoma cells depends on Bim. • Glucocorticoid-induced apoptosis of osteoblasts and insulin-producing β-cells is mediated by Bim. • Glucocorticoid-induced Bim expression is mediated by FoxO3, c-Jun and Runx1, and promoted by p38 MAPK signaling. • Glucocorticoid-mediated apoptosis of thymocytes and lymphoid malignancies depends on GSK3 and resistance is often caused by PI3K/Akt-mediated inhibition of GSK3. • GSK3 interacts with Bim following glucocorticoid treatment of thymocytes and lymphoid malignancies.	[14, 21, 49, 63, 158, 341, 458, 517-521, 523, 653]
Histone deacetylase (HDAC) inhibitors	• The HDAC inhibitor suberic bishydroxamate (SBHA) upregulated Bim, Bax and Bak in human melanoma cells. • The HDAC inhibitor Trichostatin A restored histone acetylation, with concomitant upregulation of Bim. • Trichostatin A suppressed miR-106b∼93∼25 expression through downregulation of c-Myc, thereby increasing Bim expression and apoptosis in human endometrial cancer cells. • The HDAC inhibitor vorinostat increased Bim expression and sensitized EGFR-mutant non-small-cell lung cancer (NSCLC) to the tyrosine kinase inhibitor gefitinib.	[32, 242, 245, 272, 503, 539]
Imatinib (STI571)-induced apoptosis	• Bim expression is downregulated in Bcr-Abl^+^ leukemia cells. • STI571 increases FoxO3a-dependent transcription of Bim, which was shown to be important for apoptosis. • Imatinib induces the expression of hypophosphorylated Bim_EL_ in K562 and BV173 chronic myelogenic leukemia cells. • Bim knockdown reduced susceptibility to imatinib-induced apoptosis. • Imatinib induces apoptosis of c-Kit-dependent gastrointestinal stromal tumor cells.	[121, 248, 249, 304, 305, 501, 549, 646]
Erlotinib-induced apoptosis	• The EGFR kinase inhibitor Erlotinib increased Bim expression in lung cancer cells sensitive to the drug, but not in resistant cells. • Bcl-2 inhibits the cell death induced by erlotinib.	[308, 309]
Gefitinib-induced apoptosis	• Bim is involved in gefitinib-induced apoptosis in sensitive EGFR-mutant cancer cells. • T790M mutation in EGFR prevented gefitinib-induced upregulation of Bim and apoptosis. • T790M mutated cells responded with Bim upregulation and apoptosis when using the irreversible tyrosine kinase inhibitor CL-387,785.	[306, 307]
Lapatinib-induced apoptosis	• Lapatinib treatment of sensitive, but not resistant, HER2^+^ breast cancer cells led to increased Bim expression. • Downregulation of PTK6 induced apoptosis of resistant breast cancer cells through a Bim-dependent mechanism.	[500]
Mutant B-Raf^V600E^ inhibitor-induced apoptosis	• Inhibition of B-Raf^V600E^ in melanoma cells by PLX4720 led to induction of the three Bim isoforms Bim_EL_, Bim_L_, and Bim_S_, but the increase in Bim_S_ was the most profound. • The splicing factor SRp55 was responsible for the increased Bim_S_ splicing. • Dual treatment of PTEN-negative melanoma cells with PLX4720 and a PI3K inhibitor enhanced Bim expression and apoptosis. • Combining the B-Raf inhibitor vemurafenib with the MEK inhibitor trametinib increased Bim expression and apoptosis.	[235, 541, 633]
Paclitaxel (Taxol)-induced apoptosis	• Paclitaxel induced FoxO3a and Bim expression in MCF-7 cells, both involved in apoptosis. • Knockdown of Bim decreased the susceptibility of non-small-cell lung cancer (NSCLC) cells to paclitaxel-mediated killing. • siRNA silencing of Bim reduced sensitivity of K562 cells to Taxol-induced cell death.	[118, 346, 482, 654, 655]
PP2A activator	• The PP2A activator FTY720 induces apoptosis of chronic myelogenic leukemia cells through activation of Bim and Bid. • FTY720 overcomes tyrosine kinase inhibitor resistance caused by Abl kinase domain mutations.	[656]

The pro-apoptotic effect of Bim depends on Bak and Bax [54], as do most apoptotic stimuli [15, 55]. A constitutively active form of Bim induces apoptosis in cells derived from either Bax^−/−^ or Bak^−/−^ animals, but failed to do so in Bax^−/−^Bak^−/−^ double KO cells [54]. Activation of Bak and Bax leads to homo-oligomerization and assembly within the mitochondrial outer membrane (MOM) followed by MOM permeabilization (MOMP), cytochrome C release, and initiation of the intrinsic apoptotic pathway [12, 56]. Bim also leads to uncoupling of mitochondrial respiration and the subsequent increase in the cellular levels of reactive oxygen species (ROS) [57].

While many studies have demonstrated the ability of Bim to bind to anti-apoptotic proteins, less is known on how Bim activates Bax and Bak. In the indirect activation model, binding of BH3-only proteins with the anti-apoptotic Bcl-2 proteins leads to the release of Bak and Bax, making them available to induce apoptosis [58]. This displacement mechanism takes place during Tumor necrosis factor α (TNFα)-induced apoptosis of PC12 and MCF7 cells [59]. This study showed that the interaction between Bcl-xL and Bax decreased after TNFα treatment, while the interaction between Bcl-xL and Bim_L_ increased [59]. Similarly, UV-induced apoptosis led to Bim_L_ binding to Bcl-xL with subsequent Bax release [60]. TCR ligation of developing thymocytes led to upregulation of Bim, and increased Bim binding to Bcl-xL [46], while B cell antigen receptor (BCR) ligation led to increased Bim binding to Bcl-2 [36]. However, the indirect Bax/Bak activation model seems to be only part of the story in light of the fact that only a small fraction of Bax and Bak is bound to pro-survival proteins, and in most cell types, Bax is located in the cytosol away from the anti-apoptotic proteins [61, 62]. Moreover, Bim activation can also lead to apoptosis in the absence of anti-apoptotic proteins, e.g., following glucocorticoid treatment of double negative thymic lymphoma cells lacking anti-apoptotic Bcl-2 proteins [63-65]. Yamaguchi and Wang [66] used mutant Bim_EL_ proteins to demonstrate that Bim_EL_ may activate Bax directly, in addition to its binding to Bcl-2/Bcl-xL. Also other studies showed that Bim, especially Bim_S_, can directly activate Bax and Bak [9, 23, 24, 67, 68].

In the absence of apoptotic stimuli, the α1 helix of Bax keeps the α9 helix engaged in the dimerization pocket formed by the BH1-3 domains, maintaining Bax as a monomer in cytosol [69, 70]. The C-terminal α9 helix of Bak is constitutively inserted in the mitochondrial outer membrane, but its activity is inhibited by VDAC2, which occupies the dimerization pocket of Bak to restrict Bak in the monomeric inactive conformation [71, 72]. Upon interaction of the BH3 helix of Bim with the α1 and α6 helices of Bax, the unstructured loop between α helices 1 and 2 is displaced, and the C-terminal helix 9 is mobilized for membrane translocation [10, 69, 73]. This leads to the exposure of the Bax BH3 domain that propagates the death signal through an auto-activating interaction with the trigger site of inactive Bax monomers [73]. Activator BH3-only proteins remain associated with the N-terminally exposed Bax through the BH1 domain to drive homo-oligomerization [69].

With respect to Bak, its activation depends on the exposure of its BH3 domain, which becomes reburied in dimers [74]. The oligomerization involves the insertion of the BH3 domain of one Bak molecule into the groove of another [74]. The resulting BH3:groove dimers can be converted to larger oligomers that permeabilize mitochondria by an interface between α6 helices [75]. Genetic deletions of Bid, Bim and Puma prevented the homo-oligomerization of Bax and Bak, and cytochrome C-mediated activation of caspases in response to diverse death signals in neurons and T cells, suggesting that these three BH3-only proteins co-operate in activating Bax and Bak [68]. A recent study [76] showed that Bim preferentially activates Bax, while Bid preferentially activates Bak. This study contrasts previous studies showing direct binding of Bid BH3 helix with Bax [77] and Bim with Bak [78]. Puma was recently shown to directly bind and activate Bak and Bax [79-81], besides binding to Bcl-xL [81]. Weber et al. [78] showed that ABT-737-mediated release of Bak from Bcl-xL couldn't induce apoptosis unless Bim associates with oligomeric Bak to promote its conversion to a membrane-inserted pore.

While Bcl-2 is a biomarker of resistance to both chemotherapy and radiotherapy, the association of Bim with the anti-apoptotic Bcl-2 protein may explain why Bcl-2 overexpressing lung and breast cancer cells are more sensitive to microtubule-targeting agents such as paclitaxel and vinorelbine [82]. This paradox of an anti-apoptotic protein can be explained by the upregulation of Bim in lung cancer upon Bcl-2 overexpression that makes Bim easily available upon exposure to stress stimuli [82].

### 2.2. Interaction of Bim with the Cytoskeleton

In some cell types (e.g., breast carcinoma), Bim seems to affect cytoskeletal integrity through sequestration to microtubule-associated dynein motor complexes by binding tightly to the LC8 cytoplasmic dynein L (light) chain (DYNLL1) [22]. In hematopoietic cells, Bim is not associated with microtubules, but rather associates with Bcl-2-like proteins on mitochondria [7, 18, 83]. In B and multiple myeloma cells, Bim appears to be constitutively associated with Mcl-1. At induction of apoptosis, Bim is released from Mcl-1, thus activating its pro-apoptotic function [84-86].

**Figure 3:**
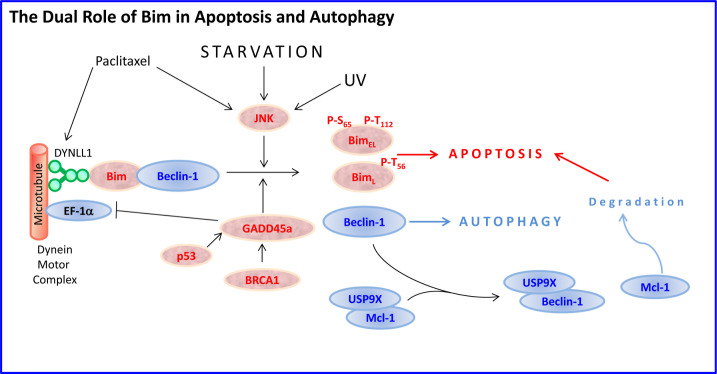
Bim at the cross-road between apoptosis and autophagy In certain cell types such as epithelial and neuronal cells, Bim_EL_ and Bim_L_ are sequestered to the dynein motor complex through interaction with the dynein light chain DYNLL1. Bim also binds Beclin-1, thus preventing autophagy. Upon exposure to microtubule-disrupting agents such as paclitaxel, or stress-stimuli such as starvation or UV radiation that activate JNK, Bim_EL_ becomes phosphorylated at Ser65 and Thr112 and Bim_L_ at Thr56, leading to their release from the microtubule and mitochondrial translocation where the intrinsic apoptotic pathway is activated. Simultaneously, Beclin-1 is released from Bim resulting in induction of autophagy. Beclin-1 also competes with Mcl-1 for the USP9X deubiquitinase resulting in enhanced Mcl-1 degradation, thereby tipping the Bim/Mcl-1 balance towards Bim-induced apoptosis. In addition, the p53- and BRCA1-regulated stress protein GADD45a promotes Bim dissociation from the microtubules. GADD45a also inhibits the function of the microtubule-severing protein EF-1α.

Bim_EL_ and Bim_L_, but not Bim_S_ harbors the dynein-binding domain (DKSTQT) [22, 30]. Interaction of Bim with DYNLL1 facilitates the interaction of DYNLL1 with Beclin-1, leading to inhibition of autophagy [87]. Overexpression of Bim suppressed autophagy, whereas Bim KO cells showed enhanced autophagocytosis [87]. Under starvation conditions, Bim is phosphorylated by MAPK8/JNK, leading to the dissociation of the Bim-Beclin-1-DYNLL1 complex, which leads to activation of autophagy by Beclin-1 and initiation of apoptosis by Bim [88] (Figure 3). Especially the isoforms Bim_EL_ and Bim_L_ can interact with Beclin-1 [88]. Bim_L_ may also promote acidification of lysosomes required for the formation of autophagic vesicles [89].

UV-irradiation leads to phosphorylation of Bim_L_ at Thr56 by activated JNK and consequent release from the microtubule, followed by translocation to the mitochondria [90]. Bim is also released from microtubule following paclitaxel (Taxol) treatment that interferes with the normal breakdown of microtubules during cell division [91]. The p53- and BRCA1-regulated stress protein GADD45a that has been implicated in the maintenance of genomic integrity, causes dissociation of Bim from microtubule-associated components leading to Bim translocation to mitochondria [92]. The Bim accumulation in mitochondria enhances the interaction of Bim with Bcl-2, relieves Bax from Bcl-2-bound complexes, and subsequently results in release of cytochrome C into the cytoplasm [92]. Thus, Bim can be indirectly upregulated by the p53 tumor suppressor gene. Suppression of Bim inhibited GADD45a-induced apoptosis [92]. GADD45a interacts with and inhibits the function of elongation factor 1α (EF-1α), a microtubule-severing protein that plays a role in maintaining cytoskeletal stability, leading to disruption of microtubule bundling [92]. Overexpression of EF-1α leads to resistance to apoptosis induced by growth factor withdrawal, ER and genotoxic stress stimuli [93, 94].

### 2.3. The Reciprocal Roles of Mcl-1 and Bim in Regulating Apoptosis

Han et al. [95] described an intriguing interplay between Mcl-1 and Bim in Tumor necrosis factor-related apoptosis-inducing ligand (TRAIL)-induced apoptosis. In the absence of pro-apoptotic stimuli, Bim is sequestrated to Mcl-1 in tumor cells. Upon stimulation with TRAIL, caspase 8 is activated and promotes degradation of Mcl-1, resulting in the release of Bim that triggers Bax-dependent apoptosis. The Mcl-1 expression level at the mitochondrial outer membrane determines the release efficiency for the apoptogenic proteins cytochrome C, Second mitochondria-derived activator of caspase (Smac), and High temperature requirement serine protease A2 (HtrA2) in response to Bim [95]. Earlier studies by Herrant et al. [96] showed that Mcl-1 is cleaved by caspases during the induction of apoptosis in various cancer cells. The Mcl-1 cleavage results in the loss of the BH4 homolgy domain required for its anti-apoptotic activity [96]. Similarly, Mcl-1 could be degraded by the T cell granule serine protease granzyme B, again releasing Bim that promotes apoptosis [97].

Mcl-1 is subjected to tight regulation at the level of protein stability. The degradation of Mcl-1 is often, but not always, required for initiation of apoptosis [64, 98-100]. The mRNA level of Mcl-1 decreases in response to various apoptotic stimuli such as UV irradiation and staurosporine [98]. Mcl-1 is a short-lived protein, positively regulated by the mTOR signaling pathway, while negatively by the E3 ligases Mcl-1 ubiquitin ligase E3 (Mule), F-Box and WD repeat domain containing 7 (Fbw7), Tripartite motif containing 17 (Trim17) and β-transducin repeat containing E3 ubiquitin protein ligase 1 (β-TrCP1), that promote its degradation [101-106]. Phosphorylation of Mcl-1 by Glycogen synthase kinase 3 (GSK3) triggers the interaction of Mcl-1 with Fbw7 [106]. The microtubule-targeting agents Paclitaxel and Vinblastine induce phosphorylation-dependent, Fbw7-mediated degradation of Mcl-1 [107]. The mTOR complex 2 (mTORC2) stabilizes Mcl-1 by suppressing the GSK3-dependent and Fbw7-mediated degradation [108]. It is noteworthy that GSK3 is activated upon glucocorticoid-induced apoptosis, leading to interaction of GSK3 with Bim and induction of Bim-dependent apoptosis of lymphoma cells [63]. Thus, GSK3 has a dual function, the first is to reduce Mcl-1 levels, and the other to enhance Bim activity, thereby fortifying apoptosis.

The VTLISFG in the BH1 domain of Mcl-1 seems to be important for regulating its degradation [109]. The deubiquitinase Ubiquitin-Specific Protease 9X (USP9X) stabilizes Mcl-1, thereby promotes cell survival [110]. Increased USP9X expression correlates with increased Mcl-1 protein in human follicular lymphomas and diffuse large B cell lymphomas [110]. Moreover, patients with multiple myeloma overexpressing USP9X have a poor prognosis [110]. Importantly, targeting USP9X attenuates B cell acute lymphoblastic leukemia cell survival and overcomes glucocorticoid resistance [111], a death process dependent on Bim [14].

A competing BH3-ligand derived from Bim interacted with Mcl-1 and prevented its interaction with Mule, leading to increased Mcl-1 expression [112]. This suggests that Bim needs to be released from Mcl-1 prior to its degradation by Mule [112]. However, binding of the p53-regulated pro-apoptotic Noxa protein to Mcl-1 leads to its degradation [113]. The Noxa-mediated degradation of Mcl-1 required Mule, but is also mediated through interruption of the deubiquitinase USP9X with Mcl-1 [113]. Also, Beclin-1 leads to destabilization of Mcl-1 through competitive binding to USP9X [114]. USP9X inhibition using WP1130 led to Mcl-1 inhibition and sensitization of solid tumors to various chemotherapeutic agents [115].

## 3. REGULATION OF BIM EXPRESSION AND ACTIVITY

**Figure 4:**
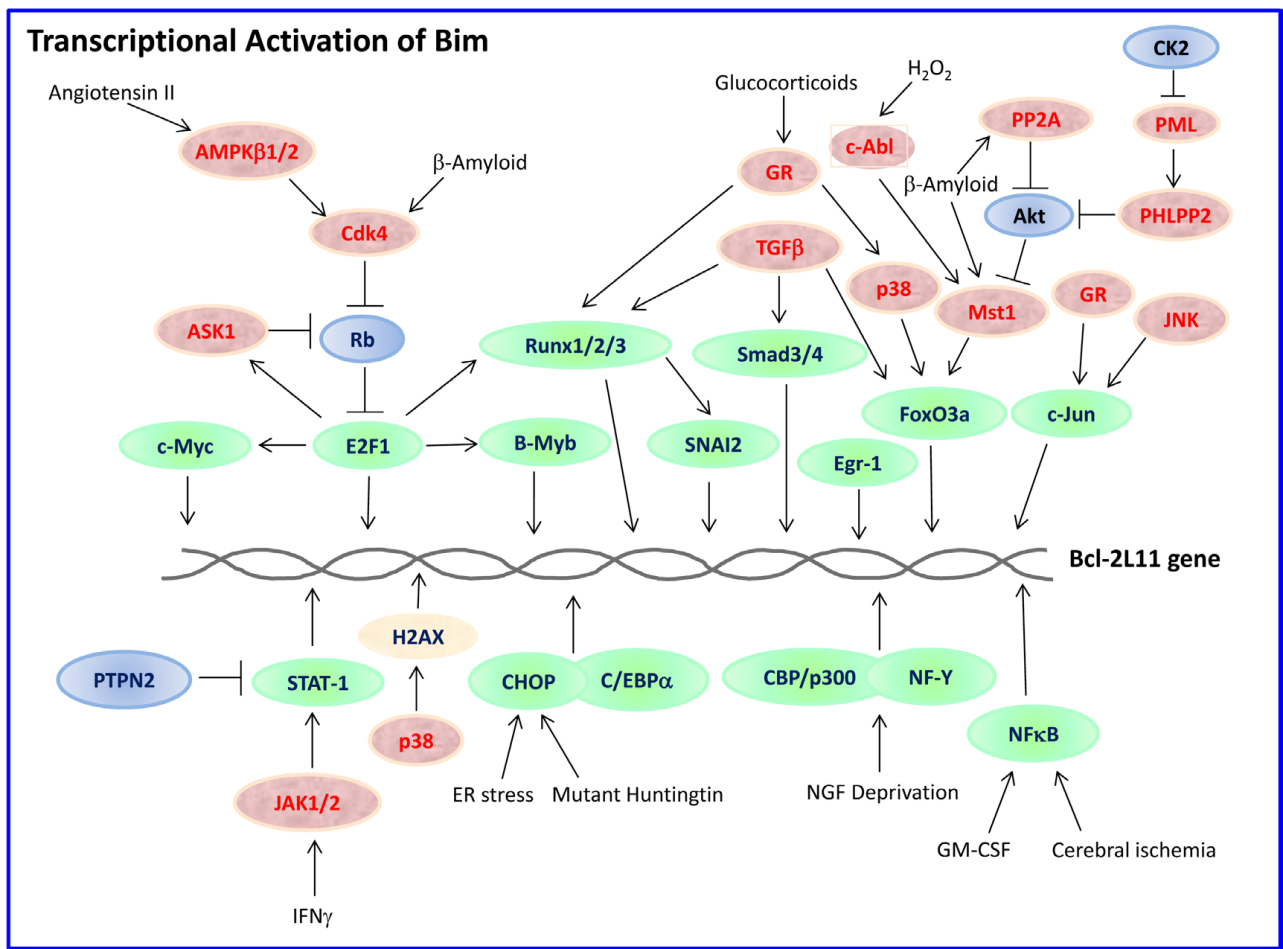
Transcriptional activation of Bim Bim transcription can be upregulated by a whole range of transcription factors (outlined in green) whose activities are tightly regulated. Some of these pathways are cell-type specific and depend on the stimulus. Those proteins positively regulating the transcription factors are presented in red, while those inhibiting in blue. H2AX is a histone whose phosphorylation at Ser139 by p38 is important for Bim transcription. The different pathways are described in more detail in Section 3.1.1.

**Figure 5:**
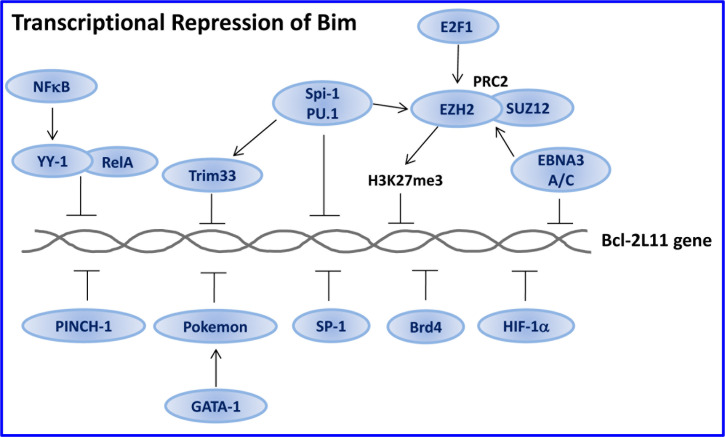
Transcriptional repression of Bim Several transcription factors prevent Bim transcription. Some of them, e.g., NFκB and E2F-1, play a dual role by regulating both Bim transactivation (Figure 4) and transrepression. The RelA component of NFκB acts as a transactivator in the absence of YY-1, while becomes a transrepressor in its presence. E2F1 may directly or indirectly transactivate Bim by inducing c-Myc and B-Myb expression (Figure 4), but, in addition, it induces the polycomb histone methyl transferase EZH2 that promotes trimethylation of Histone 3 on Lys27 (H3K27me3), leading to the repression of Bim expression. Spi-1/PU.1 and Trim33 cooperate with EZH2 to repress Bim transcription. Furthermore, E2F1 directly or through c-Myc induces miR106∼25 that prevents Bim translation (Figure 6). The multiple effects of E2F1 may ensure fine tuning of Bim expression. HIF-1α that is, among others, induced by hypoxic conditions within the tumor microenvironment, prevents Bim expression, thus providing a growth advantage to the tumor cells. Description of the other repressors can be found in Section 3.1.2.

**Figure 6:**
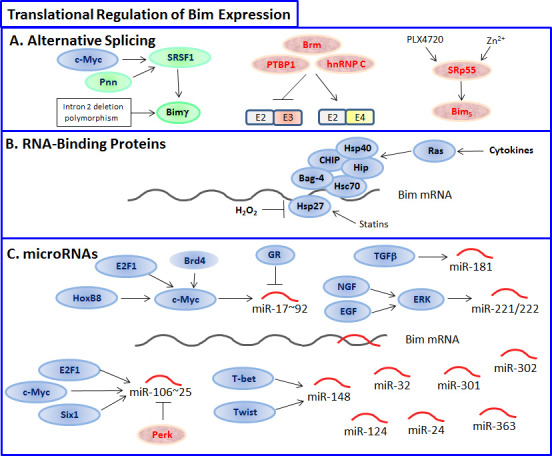
Translational regulation of Bim **A.** Various Bim isoforms can be formed by alternative splicing, where upregulation of Bim_S_ is especially efficient in inducing apoptosis, while formation of BH3-deficient Bimγ lacks pro-apoptotic activity. The splicing factor SRSF1 promotes Bimγ formation, while activation of SRp55 by the B-Raf inhibitor PLX4720 or Zn^2+^ favors Bim_S_. PTBP1, Brm and hnRNP2 favor the inclusion of the BH3-containing E4 exon over the E3 exon. B. Various RNA-binding proteins such as Hsp27 and Hsc70 prevent Bim translation. C. A range of microRNAs can target Bim, among them miR-17∼92, miR-106∼25, miR-181, miR-148 and miR-221/222 are considered as oncomiRs that can promote tumor progression.

**Figure 7:**
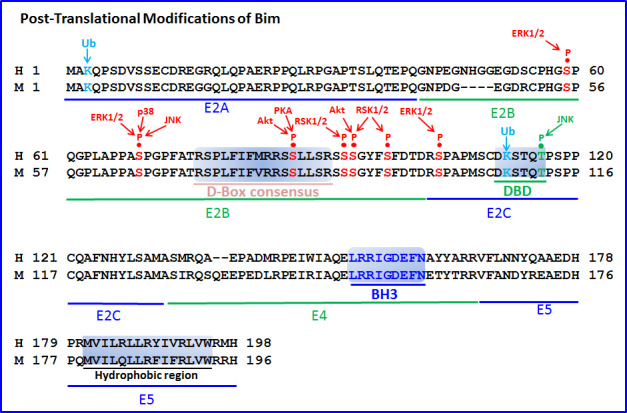
Post-translational modifications of Bim The amino acid sequences of human (H) and mouse (M) Bim_EL_ have been aligned, and important post-translational modification sites of Bim highlighted. ERK1/2 phosphorylates Bim_EL_ at three serine residues (Ser55, Ser65 and Ser100 in mouse corresponding to Ser59, Ser69 and Ser104 in human), which facilitate RSK1/2-mediated phosphorylation of Ser93/94/98 in human (corresponding to Ser89/90/94 in mouse), leading to ubiquitination at Lys3 and Lys108(M)/Lys112(H) by β-TrCP1 and proteasomal degradation. Aurora A phosphorylates the same residues as RSK1/2 during mitosis that occurs independently of previous ERK phosphorylation and leads to binding of APC^Cdc20^ to the D-box consensus region, resulting in Bim degradation. PP2A dephosphorylates Ser93/94/98 at the exit from mitosis, thereby stabilizing Bim_EL_. p38 and JNK also phosphorylate Bim_EL_ at Ser65 (M)/Ser69 (H), but, in addition, JNK phosphorylates Thr112(M)/Thr116(H) which lies within the dynein-binding domain (DBD). The latter phosphorylation leads to dissociation of Bim from the microtubules. JNK may also phosphorylate Bim_L_ at Thr56 (same residue as Thr112 in Bim_EL_). p38 and JNK phosphorylation of Bim, at least in some cell types, lead to increased Bim activity. Also, Akt and PKA can phosphorylate Bim. The coding exons (E2A, E2B, E2C, E4 and E5) have been outlined, as well as the D-box consensus, DBD, BH3 domain and the hydrophobic C-terminal region.

The expression and pro-apoptotic activity of Bim are tightly regulated at several levels (Figures 4, 5, 6, 7). Its transcription is regulated by several transcription factors that can either induce its expression (e.g., FoxO3a, E2F1, c-Myc, NF-Y, Smad1/3, Runx1-3, c-Jun and RelA) (Figure 4) or repress it (e.g., YY1, HoxB8, SPi-1/PU.1, PINCH-1, Pokemon) (Figure 5). The *bim* promoter is also epigenetically regulated through methylation of CpG dinucleotides at the 5′ end of the Bim gene. The Bim transcript undergoes alternative splicing leading to translation of various Bim isoforms with differential pro-apoptotic potential (see Section 1.2). Translation of Bim mRNA is negatively regulated by a series of microRNAs (e.g., miR-9, -181a, -17∼92, -25, -32, -221/222 and -301a) and RNA-binding proteins (e.g., Hsc70 and Hsp27) (Figure 6). The activity and stability of the Bim protein are tightly regulated at the post-translational level (Figure 7). In healthy cells, Bim can be sequestered in its inactive form to the microtubular cytoskeleton or exist as inactive heterodimers with anti-apoptotic Bcl-2 family members sequestered to the mitochondria. Upon apoptosis induction, the sequestered Bim is released from these cellular stores to promote apoptosis. Another major regulatory mechanism of Bim-dependent apoptosis is driven by phosphorylation. Phosphorylation may either increase its activity (e.g., by JNK), or promote its degradation (e.g., by ERK/MAPK) thereby antagonizing apoptosis.

### 3.1. Transcriptional regulation of Bim

A 800-bp region upstream of exon 1 contains elements for the control of Bim transcription [17]. Basal Bim mRNA levels were observed in most normal tissues [3, 6]. Binding sites for several transcription factors including FoxO, c-Myb and c-Jun are present in the *bim* promoter [17, 116-118]. Mutation of either the FoxO, c-Myb or c-Jun sites abolished induction of a *bim* promoter-derived reporter in response to nerve growth factor (NGF) deprivation [116]. This suggests that the *bim* promoter acts as a coincidence detector that optimally responds to the simultaneous activation of the the three different pro-apoptotic transcriptional pathways. Such a mechanism provides a “fail-safe” that prevents neurons from dying by accidental activation of any single pathway [116]. In addition to the proximal *bim* promoter, the first intron contains additional regulatory sequences that respond to NGF deprivation [117, 119]. This intron contains FoxO and Myb binding sites [116, 117]. Xie et al. [120] observed that early growth response 1 (Egr-1), rather than c-Jun, FoxO or c-Myb, transactivates Bim expression in cerebellar granule neurons. A putative Egr-binding sequence was found between nucleotides −56 and −47 upstream of the start site [120].

### 3.1.1. Transcriptional Upregulation

#### 3.1.1.1. FKHR-L1/FoxO3a

The forkhead transcription factor FKHR-L1/FoxO3a was found to be essential for the transcriptional activation of Bim, especially after cytokine withdrawal and various chemotherapeutic agents [118, 121, 122]. Dijkers et al. [122] demonstrated that cytokine withdrawal led to FoxO3a activation and Bim induction in T lymphocytes accompanied by apoptosis. Cytokines promote lymphocyte survival by inhibiting the activity of FoxO3a, thus preventing Bim expression [122]. Overexpression of FoxO transcription factors induces Bim expression and promotes death of sympathetic neurons in a Bim-dependent manner [117]. In neuroblastoma cells, the activation of FoxO3 triggers the intrinsic death pathway via induction of Bim and Noxa [123]. Inhibition of Breakpoint cluster region-Abelson murine leukaemia (Bcr-Abl) kinase by STI571 in Bcr-Abl expressing cells results in FoxO3a activation, induction of Bim expression and apoptosis [121]. The microtubule interfering drug paclitaxel induced Bim expression in breast cancer cells that expressed high basal levels of FoxO3a, but not in those with low basal FoxO3a levels [118]. Knockdown of FoxO3a prevented Bim induction and apoptosis induced by paclitaxel [118]. In neuroblastoma, brain-derived neurotrophic factor (BDNF) activates the PI3K/Akt pathway resulting in suppression of FoxO3a activity and Bim induction, thereby preventing paclitaxel-induced apoptosis [124]. Simultaneous activation of the MEK/ERK pathway further reduced Bim expression at the protein level [124]. FoxO3 binds to a FoxO-binding site (FHRE) within the *bim* promoter [118, 121]. FoxO3a acts in concert with the Activator protein-1 (AP-1) transcription factor [116, 117, 125].

High expression levels of FoxO3a correlated with long-term survival in breast cancer patients [126], and nuclear localization of FoxO3a is associated with longer luminal-like breast cancer survival and longer distant metastasis free interval [127]. FoxO3a expression was lower in nasopharyngeal carcinoma than in the normal nasopharyngeal tissues [128]. Nasopharyngeal carcinoma patients with low FoxO3a and high Hypoxia-inducible factor 1α (HIF-1α) expression had poorer prognosis than patients with high FoxO3a and low HIF-1α levels [128]. Low levels of FoxO factors are associated with poor prognosis in liver cancer [129] and gastric adenocarcinoma [130]. Prostate cancer with increasing Gleason grade showed marked cytoplasmic accumulation of FoxO3a, in contrast to exclusive nuclear accumulation in benign prostate cells [131]. Thus, FoxO factors act as tumor suppressors that are frequently downregulated in advanced cancers.

Since FoxO3a is an important regulator of Bim, factors affecting FoxO3a expression have direct effect on tumorigenesis. For instance, ERK phosphorylates FoxO3a at Ser294, Ser344 and Ser425, which leads to Mdm2-mediated degradation of FoxO3a via the ubiquitin-proteasome system [132]. Sirtuin 1 (SIRT1) deacetylates FoxO3a leading to its ubiquitination and degradation, thus protecting against oxidative stress-induced apoptosis of endothelial cells [133]. Similarly, IκB kinase (IKK) and Akt (PKB) phosphorylate and cause proteolysis of FoxO3a [134, 135]. Akt phosphorylation of FoxO3a leads to cytoplasmic sequestration of FoxO3a to 14-3-3 proteins, thereby preventing the transcription of genes required for apoptosis [136] Akt phosphorylates FoxO3a at Thr32 and Ser253, while the Serum- and glucocorticoid-inducible kinase (SGK) phosphorylates it at Thr32 and Ser315. Cytoplasmic FoxO3a correlated with expression of IKKβ or phosphorylated Akt in many tumors and was associated with poor survival in breast cancer [134]. Inhibition of both the mTOR and MEK/ERK pathways led to increased nuclear accumulation and activation of FoxO3a that promoted differentiation and reduced tumorigenicity of glioblastoma cancer stem-like cells [137]. p38 MAPK phosphorylates FoxO3a at Ser7 leading to its nuclear accumulation [138], and induction of Bim expression [139].

In a recent publication, Chatterjee et al. [140] described an additional level of FoxO3a/Bim regulation (Figure 4). Namely, the Casein kinase II (CK2), which is activated under various stress conditions and is abnormally deregulated in many human malignancies, indirectly suppresses FoxO3a activity by promoting phosphorylation of the Promyelocytic leukemia (PML) protein at Ser517, resulting in its ubiquitin-mediated proteasomal degradation [141]. PML is required for the functional interaction of activated phosphorylated Akt and its phosphatases (e.g., PHLPP2) inside the nucleus, that leads to inactivation of Akt [142] and proper functioning of FoxO3a [140]. In the absence of PML, Akt is aberrantly activated, leading to nuclear exclusion of FoxO3a. As a consequence, the FoxO3 target genes p27^Kip1^, p21^Cip1^ and Bim are downregulated, while VEGF gene expression, which is suppressed by FoxO3a [143], becomes upregulated [140, 142]. Thus, CK2 antagonizes apoptosis by relieving the negative effect of PML on Akt, that in turns negatively regulate FoxO3a-mediated transcription of Bim.

Although FoxO3a is a central regulator of Bim expression after growth factor and cytokine withdrawal and some chemotherapeutic agents [117, 118, 121, 122, 144], mice with mutated FoxO-binding sites within the *bim* promoter had a normal hematopoietic system, and showed similar death rates of cytokine-dependent haematopoietic cells as wild-type mice [145]. This study suggests that the direct transcriptional induction of Bim by FoxO transcription factors is not critical for Bim's pro-apoptotic function in the hematopoietic system, at least under normal homeostasis. This FoxO independence could be due to already high basal Bim expression in these cells [63, 64, 145]. The same research group showed that endothelial cell apoptosis in response to VEGF-A inhibitors is Bim-dependent, but could proceed normally in mice lacking FoxO-binding sites in the *bim* promoter [146]. As FoxO3 KO mice show altered hematopoietic homeostasis [147-149], it is likely that FoxO3a may affect Bim transcription through interaction with other transcription factors, or that there is a compensation for FoxO3.

#### 3.1.1.2. NF-Y

The heterotrimeric transcription factor NF-Y is required for Bim induction in neurons following NGF withdrawal [150]. The transcriptional coactivators CBP and p300 interact with NF-Y and FoxO3a and bind to the CCAAT box (ICB) of the *bim* promoter [150]. The amount of CBP/p300 bound to the *bim* promoter increases after NGF deprivation, and inhibition of CBP/p300 activity reduces Bim induction [150]. Thus, NF-Y cooperates with FoxO3a to recruit CBP/p300 to the *bim* promoter [150]. The C subunit of NF-Y was found to be upregulated in hippocampal CA1 neurons following cerebral ischemia concomitant with the upregulation of Bim [151].

#### 3.1.1.3. Smad2/3/4

Overexpression of Smad3, a transcription factor activated by TGFβ, increased the expression of Bim in B lymphocytes [152]. Activation of the pro-survival transmembrane glycoprotein CD40 abrogated TGFβ-mediated Bim induction and apoptosis [152]. Smad3 was also found to be involved in the transcriptional activation of Bim in human gastric carcinoma cells when simultaneously exposed to both TGFβ and TNFα [153]. Under these conditions the Bim protein was stabilized by a JNK-dependent mechanism [153]. TGFβ-induced apoptosis of human hepatoma cells was also mediated by a Smad3/Smad4-mediated upregulation of Bim [154]. TGFβ also activates FoxO3 dephosphorylation of Thr32, leading to its nuclear translocation [155]. These authors further showed that the TGFβ-mediated upregulation of Bim and apoptosis was dependent on Casein kinase Iε (CKIε) [155]. Moreover, the TGFβ-activated FoxO3 cooperated with Smad2/3 to mediate Bim upregulation and apoptosis [155].

#### 3.1.1.4. c-Jun and Runx1-3

The JNK/c-Jun pathway was found to be important for Bim upregulation in neurons following survival signaling withdrawal [116, 125, 156]. However, activity deprivation-dependent induction of Bim during apoptosis in cerebellar granule neurons occurred independently of JNK/c-Jun activation [157]. p38 MAPK that is important for the upregulation of Bim in CCRF-CEM T-ALL cells in response to glucocorticoids [158], acts upstream to the transcription factors Runx2 and c-Jun that positively activate the transcription of Bim [159]. c-Jun was upregulated in glucocorticoid-sensitive, but not glucocorticoid-resistant CCRF-CEM T-ALL cells, which correlated with Bim induction [160]. It should be noted that the requirement for p38 in the upregulation of Bim was only observed in certain leukemic cell types, while it was dispensable for Bim expression in others [63]. Another protein kinase that might contribute to Bim induction is GSK3 [161].

Runx2 may affect Bim expression through the zinc finger transcription factor Snail family zinc finger 2 (SNAI2). Runx2 induces epithelial-mesenchymal transition (EMT) through induction of SNAI expression [162], which binds to the *bim* promoter and promotes Bim expression [163]. Runx3 is responsible for the transcriptional upregulation of Bim by TGFβ [164]. Lack of Runx3 functions is causally related to the genesis and progression of gastric cancer [164]. TGFβ also induces the expression of the transcription factor Runx1 that binds to FoxO3, to enhance its ability to transcribe Bim [165]. A putative forkhead binding element (FHBE) located at position −168 to −174 from the transcription start site is involved in Bim induction by Runx1 and FoxO3a [165]. In addition, TGFβ indirectly increases Bim protein stability through Smad3-dependent induction of the mitogen-activated protein kinase (MAPK) phosphatase MKP2/DUSP4 that reduces the activity of ERK [166]. As ERK phosphorylates Bim and promotes its proteasomal degradation (see Section 3.3.1.1), inhibition of ERK leads to increased Bim stability. Thus, TGFβ increases Bim expression both at the transcriptional and post-translation levels.

Double positive thymocytes overexpressing the Runt domain of Runx1 expressed elevated levels of Bim [167]. As the Runt domain is expected to act as a dominant negative form of Runx1, Abe et al. [167] suggested that Runx1 suppresses the apoptotic sensitivity of double positive thymocytes in the context of T cell receptor signaling.

#### 3.1.1.5. E2F1

E2F transcription factors are best known for their involvement in the timely activation of genes required for cell cycle progression. E2F activity is negatively regulated through its interaction with the retinoblastoma (Rb) tumor suppressor. Human tumors with inactive Rb pathway, often show deregulated E2F1 activity. Ectopic expression of E2F1 resulted in induction of Bim expression and apoptosis [168]. Also the BH3-only proteins PUMA, Noxa and Hrk/DP5 were upregulated by E2F1 [168]. Inhibitors of histone deacetylases (HDACi) were shown to target the Rb/E2F1 pathway for apoptosis induction through activation of Bim [169]. Cancer cells with elevated E2F1 activity were highly sensitive to HDACi-induced cell death [169]. E2F1 also transactivates apoptosis signal-regulated kinase 1 (ASK1) that further enhances E2F1-mediated Bim transcription through inhibition of Rb [170]. ASK1 knockdown led to reduced E2F1-induced Bim transcription and reduced apoptosis in response to the HDAC inhibitor suberoylanilide hydroxamic acid (SAHA) [170].

Spinal cord injury led to strong induction of E2F1 and Bim followed by neuronal apoptosis that could be prevented by inhibition of E2F1 [171]. Angiotensin II, a key pro-apoptotic factor in fibrosis, mediates apoptosis in primary pulmonary artery endothelial cells through E2F1-mediated upregulation of Bim [172]. Angiotensin II regulates the association of AMP-regulated protein kinase β1/2 (AMPKβ1/2) with cyclin-dependent kinase 4 (Cdk4) leading to hyperphosphorylation of Rb and the release of E2F1 for transcriptional activation [172].

E2F1 also induces the expression of the oncogenic polycomb histone methyltransferase enhancer of zeste homolog 2 (EZH2), that antagonizes the induction of Bim by E2F1 [173] as well as the transcription of Runx3 [174] that also upregulates Bim [164]. Thus, the apoptotic activity of E2F1 is restrained in human cancer by the concomitant induction of EZH2 and Bim [173]. Moreover, deregulated E2F1 activity in gastric cancer may confer resistance to TGFβ-induced apoptosis through upregulation of the miR-106∼25 cluster that targets Bim [175]. Thus, E2F1 has opposing effects on Bim expression.

Upregulated Bim expression in prostate and breast cancer cells was dependent on E2F1, where E2F1 silencing led to loss of Bim [176]. These authors identified eight endogenous E2F1-binding sites in the Bim promoter. However, FoxO3a didn't bind to the Bim promoter in these cancer cells [176]. Interestingly, Gogada et al. [176] observed that Bim silencing or microinjection of anti-Bim antibodies into the cell cytoplasm of breast cancer cells resulted in cell rounding, detachment, and subsequent apoptosis, suggesting that Bim might have a pro-survival role in addition to being pro-apoptotic. The low pro-apoptotic activity of Bim in the epithelial cancer cells was explained by sequestration of Bim to microtubules, Bcl-xL and Mcl-1 together with low expression of Bax and Bax and elevated expression of X-linked inhibitor of apoptosis protein (XIAP) that inhibits caspase 9 and caspase 3 [176]. Since Bim associates with the microtubule, it could be that Bim is important for stabilizing the cytoskeleton required for survival. Alteration in cytoskeleton integrity might affect mitochondrial respiration and apoptosis [177].

#### 3.1.1.6. CHOP-C/EBPα

Bim was found to be essential for ER stress-induced apoptosis in thymocytes, macrophages, and epithelial cells from breast and kidney [35]. In other cell types, e.g., neuroblastoma, colon carcinoma and mouse embryonal fibroblasts (MEFs), ER-stress-induced apoptosis can also be mediated by Puma and Noxa [178, 179]. ER stress activates Bim through two different pathways. One involves direct transcriptional activation by the C/EBP homologous protein (CHOP/GADD153)-C/EBPα transcription factors, while the other involves protein phosphatase 2A (PP2A)-mediated dephosphorylation, which prevents its ubiquitin-dependent proteasomal degradation [35]. Also, mutant huntingtin that is responsible for the selective loss of medium spiny neurons in the striatum of patients with Huntington's disease, upregulates both GADD153 and Bim_EL_ [180]. Knockdown of Bim_EL_ prevented mutant huntingtin-induced apoptosis [180]. In thapsigargin-treated MCF-7 cells, FoxO3a and FoxO1 undergo dephosphorylation after Bim induction, suggesting that FoxO3a is not important in this setting [35]. FoxO3a-deficient thymocytes are normally sensitive to thapsigargin and tunicamycin [35]. According to Gosh et al. [181], CHOP co-operates with FoxO3a to upregulate Bim and Puma in response to ER stress in cortical neurons. Co-immunoprecipitation studies showed that CHOP interacts with FoxO3a [181]. CHOP may also co-operate with Activating transcription factor 4 (ATF4) for transcriptional upregulation, as seen for Puma in cortical neurons [182].

#### 3.1.1.7. STAT-1

The transcription factor Signal transducer and activator of transcription 1 (STAT-1) has been shown to be involved in the induction of Bim during TNFα- and IFNγ-induced pancreatic β-cell apoptosis [183]. IFNγ induces Bim transcription [183], while TNFα activates JNK that phosphorylates, and thereby upregulates Bim protein expression by protein stabilization [184]. IFNγ activates signal transduction pathways that involve the tyrosine Janus kinases JAK1 and JAK2, which phosphorylate and induce the dimerization of STAT-1 [185]. Silencing of Bim or STAT-1 protected β-cells from cytokine-induced apoptosis [183, 186]. STAT-1 directly binds to the *bim* promoter in positions −686 to −385 [183]. This region contains two STAT-1 binding sites, TTCtacGAA and TTCttgGAA [183]. Knockdown of the PTPN2 phosphatase, a candidate gene for type 1 diabetes [187], led to increased phosphorylation and activation of STAT-1 in β-cells, and increased cytokine-induced Bim expression [183, 188]. Diabetic retinopathy caused by high glucose-induced apoptosis of retinal pericytes involved STAT-1-mediated Bim expression [189]. TNFα was responsible for the high glucose-mediated activation of STAT-1, while Bim was responsible for the high glucose-induced ROS production [189]. STAT-1-dependent Bim transactivation was also observed in chronic lymphoblastic leukemia cells after exposure to IL-21, a gamma-chain receptor cytokine family member that promotes B cell apoptosis [190].

#### 3.1.1.8. c-Myc

c-Myc is one of the most frequently overexpressed oncogenes in human cancer and promotes ectopic proliferation in many post-mitotic cells. However, deregulated c-Myc expression sensitizes the cells to apoptosis by a Bim-dependent mechanism [191]. c-Myc was found to bind to the *bim* promoter and promote Bim transcription [191, 192]. Bim induction by c-Myc was independent of the p53 tumor suppressor that is also activating by wild-type c-Myc [193]. Mutant c-Myc, however, was unable to induce Bim expression, while still activated the p53 pathway to a similar extent as wild-type c-Myc [193]. This may explain why tumors with mutant c-Myc are less prone to apoptosis, and mutant c-Myc is more oncogenic [193]. Of note, wild-type and mutant c-Myc were equally oncogenic in the absence of Bim [193], further emphasizing the role of Bim in antagonizing the oncogenic effects of c-Myc. Hemann et al. [193] further showed that Burkitt's lymphoma expressing wild-type c-Myc displayed substantially higher Bim levels than diffuse, large B cell lymphomas (DLBCL) without c-Myc translocation. In contrast, Burkitt's lymphomas with mutated c-Myc were usually Bim negative [193]. The differential effect of wild-type and mutant c-Myc on Bim expression may also explain the opposing effects described for c-Myc on glucocorticoid-induced apoptosis [194-198], a death process dependent on Bim [199]. Moreover, c-Myc may reduce Bim expression through induction of miR-17∼92 [200-202] (see Section 3.2.2.2).

c-Myc was also found to be responsible for Bim overexpression in human epidermal growth factor receptor 2 (HER2)-overexpressing breast cancer cells [192]. c-Myc expression in these cells was dependent on mTORC1 activity [192]. A simultaneous elevation of Mcl-1 in naïve HER2-positive cancer cells antagonized the pro-apoptotic effect of Bim [192]. Reduction of Mcl-1 expression was sufficient to induce apoptosis of HER2-overexpressing cells, suggesting that cell survival/death is determined by the Mcl-1/Bim balance [192]. Similar findings were observed by Horiuchi et al. [203] in triple-negative breast cancer that overexpressed c-Myc. c-Myc overexpression led to increased expression of Bim and the anti-apoptotic proteins Bcl-2, Bcl-xL and Mcl-1 [203]. The elevated c-Myc expression sensitized the triple-negative breast cancer cells to CDK inhibition by either Purvalanol A or Dinaciclib that was dependent on Bim [203].

#### 3.1.1.9. Nrf2

The nuclear factor erythroid-derived 2, like 2 (Nrf2) is a key regulator of the antioxidant defense system that is activated under hyperprolifeation conditions in the liver. Activated Nrf2 delays proliferation and induces apoptosis of hepatocytes in the regenerating liver through direct transcriptional upregulation of Bim [204].

### 3.1.2. Transcriptional Repression

#### 3.1.2.1. YY-1-RelA

In multiple myeloma, Bim transcription is repressed by the Yin Yang 1 (YY1)-RelA (p65) complex [205]. RelA is a NFκB subunit, while YY1 is a target gene of NFκB [205]. Deletion of either YY1 or RelA was sufficient to increase the expression level of Bim, with subsequent induction of apoptosis [205]. All three Bim isoforms, Bim_EL_, Bim_L_ and Bim_S_, were upregulated upon repression of either YY1 or RelA [205]. This research group further showed that YY1 and RelA need to be co-expressed for transcriptional repression of Bim [205]. YY1 and RelA bind within a region spanning between nucleotides −300 and −156 of the *bim* promoter [205]. However, under conditions of cerebral ischemia, RelA functioned as a transcriptional activator of Bim [206]. It could be that the interaction of YY1 with RelA is involved in switching RelA from being an activator to a repressor of the *bim* promoter.

#### 3.1.2.2. HoxB8

In hematopoietic progenitor cells, Homeobox B8 (HoxB8) overexpression repressed Bim through a c-Myc-dependent upregulation of miR-17∼92 that binds to the 3′UTR of Bim mRNA [207]. HoxB8 overexpression immortalizes hematopoietic progenitor cells in a growth factor-dependent manner and co-operates with IL-3 to induce acute myeloid leukemia. Downregulation of HoxB8, in the presence of IL-3, caused cell-cycle arrest and apoptosis that was dependent on Bax and Bak, and in part, on Bim [207]. The requirement of miR-17∼92 to suppress Bim expression by HoxB8 is evident in the strong selection against deletion of miR-17∼92 in HoxB8-immortalized myeloid cells [207].

#### 3.1.2.3. Spi-1/PU.1

In erythroleukemia, the transcription factor Spi-1/PU.1 inhibits apoptosis through transcriptional repression of Bim [208]. Spi-1 represses Bim transcription by binding to the *bim* promoter and by promoting trimethylation of histone 3 on Lys27 (H3K27me3, a repressive histone marker) on the *bim* promoter [208]. The Polycomb-repressive complex 2 (PRC2) is responsible for the histone methylation [208]. A Spi-1 binding site was identified 0.3 kb upstream of the *Bim* transcriptional start site [208]. The two PRC2 subunits SUZ12 and the histone methyltransferase EZH2 interact with Spi-1 and bind to the *bim* promoter at the H3K27me3 sites that are 0.9 kb distant from the Spi-1 binding motif [208]. The transcription co-factor Tripartite motif containing 33 (Trim33) is recruited to an enhancer upstream to the Bim gene by PU.1, leading to suppression of Bim expression and protection of B lymphoblastic leukemia from apoptosis [209].

#### 3.1.2.4. PINCH-1

Particularly interesting cysteine-histidine-rich protein-1 (PINCH-1) is a cytoplasmic component of cell-extracellular matrix adhesions required for protection of multiple types of cancer cells from apoptosis. Although PINCH-1 is not a transcription factor, its knockdown leads to a 10-fold increase in Bim_EL_ mRNA levels, suggesting for a role of PINCH-1 in suppressing Bim transcription [210]. Depletion of Bim blocked apoptosis induced by the loss of PINCH-1 [210]. Besides affecting Bim transcription, PINCH-1 promotes activating phosphorylation of Src family kinases and ERK1/2 that leads to Ser69 phosphorylation of Bim resulting in enhanced degradation [210].

#### 3.1.2.5. Pokemon/Lrf

The erythroid myeloid ontogenic factor Pokemon (also known as Lrf/Zbtb7a) is a member of the POK (POZ and Krüppel) family of transcriptional repressors that is a master regulator of B and T cell lymphoid fates and erythroid development and maturation [211, 212]. Pokemon was found to directly repress Bim transcription through a tandem-binding site in the *bim* proximal promoter region [212]. As Pokemon is a downstream target of GATA-1, the expression of GATA-1 indirectly promotes erythroid cell survival [212]. Thus, Pokemon facilitates erythroid differentiation and survival through prevention of Bim expression. Maeda et al. [212] further showed that Bim deficiency rescues the anemia observed in Lrf/Pokemon KO mice.

#### 3.1.2.6. SP-1

SP-1 (Specificity Protein 1) is often overexpressed in cancer and may be a negative prognostic factor for the overall survival [213, 214]. The *bim* promoter contains six GGGCGG motifs that are recognized by the transcription factor SP-1 [17]. Inhibition of SP-1 by the curcumin analogue dibenzylideneacetone, led to increased expression of Bim and apoptosis in mucoepidermoid carcinomas [215], suggesting that SP-1 acts as a suppressor of Bim expression. Also mithramycin A, another SP-1 inhibitor induced Bim expression and apoptosis [215]. SP-1 may positively regulate the anti-apoptotic Mcl-1 expression [216], such that inhibition of SP-1 has dual pro-apoptotic effects.

#### 3.1.2.7. Brd4

In malignant peripheral nerve sheath tumors (MPNSTs), Bim expression is repressed by the chromatin regulator Brd4, which is a member of the bromodomain and extra-terminal (BET) protein family [217]. Brd4 contains two bromodomains in tandem that permit recognition and binding to acetylated histones, and subsequent recruitment of co-factors (including pTEFb) for RNA polymerase II-dependent elongation, resulting in the upregulation of c-Myc and Bcl-2. Inhibition of Brd4 with either of the small molecule inhibitors JQ1, I-BET 151 or CPI203, induced apoptosis of these malignant cells through induction of Bim along with downregulation of Bcl-2 and Cyclin D1 [217]. Cotreatment with the Brd4 inhibitor JQ1 and the HDAC inhibitor panobinostat synergistically induced apoptosis of human acute myelogenous leukemia (AML) blast progenitor cells (BPC) through attenuation of c-Myc and Bcl-2 expression, while increasing p21 and Bim expression [218]. Similarly, the Brd4 inhibitor I-BET151 induced Bim-dependent apoptosis in melanoma cells that was not affected by the B-Raf and N-Ras mutational status [219]. JQ1 also had a synergistic effect on Flt3 tyrosine kinase inhibitor (ponatinib)-induced apoptosis of CD34^+^ human AML BPC cells, which led to increased upregulation of Bim [220]. JQ1 downregulated c-Myc and Birc5 expression in primary paedriatric B-precursor acute lymphoblastic leukemia (B-precursor ALL) and sensitized the malignant cells to dexamethasone-induced apoptosis [221]. The therapeutic effect of JQ1 was dependent on basal high c-Myc expression [221].

#### 3.1.2.8. HIF-1α

Sympathetic neurons exposed to low O_2_ tension upregulate hypoxia-inducible factor-1α (HIF-1α) that protects against NGF-deprivation-induced apoptosis through suppression of Bim expression [222]. The Tax protein of human T-lymphotropic virus type 1 (HTLV-1) downregulates Bim expression at the transcriptional level through induction of HIF-1α [223]. Knockdown of HIF-1α or chemical inhibition of its transactivation activity resulted in increased Bim expression and sensitization of HTLV-1-infected leukemic T cells to CD95/TRAIL- and anticancer drug-induced apoptosis [223]. HIF-1α may also reduce Bim protein expression through ERK-dependent phosphorylation and degradation [224].

#### 3.1.2.9. EBNA3A and EBNA3C

Epstein-Barr virus (EBV) contributes to the development of various human cancers including the endemic form of Burkitt's lymphoma. The nuclear factors EBNA3A and EBNA3C repress Bim transcription that leads to the establishment of lymphoblastoid cell lines from primary B cells [225]. EBNA3C binds near the transcription start site and recruits PRC2 core subunits that are required for Bim repression [226]. McClellan et al. [227] identified EBNA binding sites between +87 and +921, which are downstream to the transcriptional start site described by Paschos et al. [226].

### 3.1.3. Alternative splicing

As described in Section 1.3, the Bim transcripts undergo extensive alternative splicing, giving rise to a variety of Bim isoforms with different intrinsic toxicities and modes of regulation [3, 25, 28]. Splicing of exon 3 and splicing of exon 4 usually occur in a mutual exclusive manner, and due to the presence of a stop codon and a polyadenylated signal within exon 3, its inclusion leads to a pre-mature Bim protein that lacks the BH3 domain required for apoptosis [30, 31, 228]. Intron 2 deletion polymorphism led to preferential splicing of exon 3 over exon 4, thus excluding the BH3 encoded by exon 4 [31]. Bimγ formed by the splicing of exon 3 had a very short half-life of less than an hour [31].

The splicing factor oncoprotein SRSF1 (also known as SFSR1 or SF2/ASF) promoted alternative splicing of Bim to produce isoforms that lack pro-apoptotic functions [229]. Two new isoforms termed Bimγ1 and Bimγ2 were generated due to the inclusion of an alternative 3′ exon and exclusion of exons E4 and E5, thus lacking the BH3 domain [229]. The expression of the isoforms Bim_EL_, Bim_L_ and Bim_S_ was concomitantly reduced [229]. SRSF1 is frequently upregulated in breast cancers and its overexpression leads to the formation of larger acinar cells, due to increased proliferation and delayed apoptosis during acinar morphogenesis [229]. The alternative splicing of Bim is important for the reduced apoptosis of acinar cells [229]. High SRSF1 expression occurs frequently in tumors overexpressing c-Myc, and these tumors are of higher histological grade that those with low c-Myc and SRSF1 expression [229]. Further studies showed that SRSF1 is a target gene of c-Myc [230]. Loss of SRSF1 induces G2 cell cycle arrest and apoptosis [231]. Knockout of the serine/arginine-rich (SR)-related protein Pinin (Pnn) leads to early embryonic lethality, and its knockdown in breast cancer cells leads to apoptosis due to reduced expression of SRSF1 and increased expression of Bim [232].

The human Krüppel-like zinc finger protein Gli-similar (GLIS) 3 whose mutations lead to neonatal diabetes, seem to indirectly affect Bim splicing [233]. Knockdown of GLIS3 leads to preferential expression of Bim_S_ and β-cell apoptosis that was further aggravated by cytokines [233]. Simultaneous knockdown of Bim abrogated the pro-apoptotic effect of GLIS3 [233]. One mechanism by which GLIS3 prevents Bim_S_ isoform formation is through induction of the splicing factor SRp55 (encoded by the gene splicing factor arginine/serine (RS)-rich 6 (*SFRS6*)) [233]. Overexpression of GLIS3 in β-cells antagonized cytokine-induced apoptosis [233] and SRp55 expression was modified in human islets following cytokine treatment [234]. The observation by Nogueira et al. [233] showing that downregulation of SRp55 in β-cells leads to increased expression of Bim_S_ and apoptosis, opposes the report of Jiang et al. [235] showing that SRp55 appears necessary for the preferential increase in Bim_S_ splicing observed after treating B-Raf^V600E^ mutant human melanoma cells with the B-Raf^V600E^ inhibitor PLX4720. The apparent discrepancy could be due to cell-specific effects. Also, Hara et al. [236] observed that SRp55/SFRS6 leads to preferential upregulation of the Bim_S_isoform following treatment of human neuroblastoma cells with Zn^2+^, that induces apoptosis of these cells. They identified a SFRS6 binding site in the intronic region adjacent to exon 4 [236].

Juan et al. [237] studied the 2,903-bp deletion polymorphism within Bim intron 2 that biased towards exon 3, leading to impaired Bim-dependent apoptosis. They observed that this region has many cis-acting elements that repress exon 3 inclusion [237]. A 23-nt intronic splicing silencer at the 3′-end of the deletion was found to be important for the exon 3 exclusion [237]. They demonstrated that Polypyrimidine tract binding protein 1 (PTBP1) and Heterogeneous nuclear ribonucleoprotein C (hnRNP C) repress exon 3 inclusion, and downregulation of PTBP1 inhibits Bim-mediated imatinib-induced apoptosis [237].

The catalytic subunit Brm (Brahma) of the SWI/SNF related, matrix associated, subfamily a, member 2 (also known as mating-type switch/sucrose nonfermenting) complex involved in chromatin remodeling on promoters was shown to associate with several components of the spliceosome and with Sam68, an ERK-activated enhancer of variant exon inclusion [238]. Brm favors the inclusion of variant exons in the mRNA of Bim, in addition to increasing the Bim expression level [238]. The BH3-containing exon 4 (that they coined exon 5) was included between exon 2 and 5 (that they coined exon 7), giving rise to the Bim_L_ and Bim_EL_ isoforms [238]. The Brm gene codes for an ATPase catalytic subunit that shifts histones and opens the chromatin and is considered to be a tumor suppressor gene [239].

As Bim_S_ can directly bind and activate Bax [9, 23, 24], and is not sequestrated to the cytoskeleton [22] or negatively regulated by the MEK/ERK pathway [17, 25], makes its preferential upregulation a significant contribution to apoptosis. The more potent pro-apoptotic function of Bim_S_ may explain why this isoform is barely expressed under basal conditions, in contrast to the Bim_EL_ isoform that is abundantly expressed [14, 17, 20, 25, 63, 64].

### 3.1.4. Epigenetic Regulation of the *bim* and *foxO3* Promoters

Bim expression is also epigenetically regulated. Histone deacetylase inhibitors (HDACi) induce Bim expression in various tumors, including multiple myeloma, melanoma, pancreatic adenocarcinoma, Burkitt's lymphoma and CML [240-245]. Silencing of the *bim* promoter by hypermethylation has been shown in B cell lymphomas [246]. In human B cells infected with Epstein-Barr virus (EBV), latency-associated virus gene products inhibit expression of Bim and enhance cell survival. Besides the involvement of the EBV nuclear proteins EBNA3A and EBNA3C in repressing Bim transcription, latent EBV initiates a chain of events that leads to epigenetic repression of Bim in infected B cells and their progeny [243]. There was a significant methylation of CpG dinucleotides within the large CpG island located at the 5′ end of *bim* in EBV-positive, but not in EBV-negative B cells [243]. Paschos et al. [243] also observed that EBV-mediated repression of Bim was associated with reduced acetylation of histones H3 and H4. IGF-1 treatment of multiple myeloma cells led to reduced histone H3 tail Lys9 (H3K9) acetylation, and increased H3K9 dimethylation, which contributed to the silencing of the *bim* and *foxO3* genes [247]. Patients with Burkitt's lymphoma with hypermethylated *bim* showed lower complete remission rate and shorter overall survival than those with Bim-expressing lymphomas [244].

Downregulation of Bim expression was observed in 36% of Bcr-Abl-positive chronic myeloid leukemia patients that could explain their sub-optimal response to imatinib [248]. The reduced expression of Bim was due to promoter hypermethylation that could be prevented by 5-aza-2′-deoxycytidine treatment [248]. The MAPK p38-mediated phosphorylation of the histone H2AX at Ser139 was found to be necessary for the imatinib-induced Bim expression in chronic myeloid leukemia cells [249]. H2AX regulates DNA repair after being phosphorylated at Ser139 by ATM [250], but is involved in apoptosis of cancer cells that depends on the phosphorylation of the same serine residue by Mst1 (mammalian STE20-like kinase 1) [251] or JNK [252]. Knockout of H2AX blocked apoptosis [252].

### 3.2. Post-Transcriptional Regulation

#### 3.2.1. Regulation of Bim mRNA through the 3′-Untranslated Region (3′-UTR)

Translation of Bim mRNA can be regulated through the 3′-untranslated region (3′-UTR) [253, 254]. This region binds microRNAs and RNA-binding proteins (RBPs) that regulate mRNA stability and/or translation [255]. Cytokines negatively regulate the steady-state levels of Bim through heat-shock cognate protein 70 (Hsc70), which binds to AU-rich elements (AREs) in the 3′-UTR [253]. The RNA binding potential of Hsc70 is regulated by the co-chaperones Bag-4 (SODD), CHIP, Hip, and Hsp40. Cytokines regulate the expression or function of these co-chaperones by activating Ras pathways. Thus, exposure of cells to cytokines ultimately leads to destabilization of Bim mRNA and promotion of cell survival [253].

Heat shock protein 27 (Hsp27) prevents oxidative stress-induced cell death in cerebellar granule neurons by binding to the 3′-UTR of the Bim mRNA, thereby preventing translation of Bim protein [256]. Oxidative stress induced by hydrogen peroxide led to Hsp27 depletion and increased Bim expression, resulting in subsequent neuronal death [256]. Of note, HMG-CoA reductase inhibitors (statins) increased Hsp27 expression [257] and prevented oxidative stress-induced apoptosis of endothelial progenitor cells by inhibiting FoxO4-mediated upregulation of Bim [258].

#### 3.2.2. microRNAs Regulating Bim Expression

Bim expression is negatively regulated by a variety of microRNAs. The importance of microRNAs in Bim regulation is demonstrated in embryonic stem cells deficient in the Argonaute (Ago) 1-4 proteins that are the core effectors of the microRNA pathway [259]. These embryonic stem cells are defective in microRNA silencing, show upregulated expression of the three isoforms of Bim (Bim_EL_, Bim_L_ and Bim_S_) and undergo apoptosis [259]. The upregulation of Bim was sufficient to induce apoptosis that could be prevented by simultaneous expression of activated Akt [259]. Reintroduction of any single Ago into Ago-deficient cells was able to rescue the endogenous miRNA silencing defect and apoptosis [259]. Similarly, Dicer-deficient pro-B cells showed enhanced upregulation of Bim and developmental block at the pro- to pre-B cell transition with excessive apoptosis at the pre-B cell stage [260]. B cell development could be partially rescued by ablation of Bim or transgenic overexpression of Bcl-2 [260].

##### 3.2.2.1. miR-9 and miR-181a

miR-9 and miR-181a were downregulated in the synovial fluid cells after tibial plateau fractures, leading to increased expression of the Cbl E3 ubiquitin ligase involved in bone formation and homeostasis regulation [261]. The elevated levels of Cbl led to increased Bim ubiquitination and degradation, resulting in enhanced osteoclast survival and activation [261]. Also, TGFβ upregulates miR-181 expression to promote breast cancer metastasis [262]. Inactivation of miR-181a elevated the expression of Bim, which sensitized metastatic cells to anoikis [262]. This research group further showed that miR-181a expression was essential in driving pulmonary micrometastatic outgrowth and enhancing the lethality of late-stage mammary tumors in mice [262]. miR-181a expression was upregulated in metastatic breast tumors, particularly triple-negative breast cancers, and was highly predictive for decreased overall survival in human breast cancer patients [262].

##### 3.2.2.2. The miR-17∼92 and miR-106b∼25 clusters

The 3′-UTR of Bim mRNA contains nine potential binding sites for miR-17∼92 family members with at least one binding site for each of the three distinct microRNA seeds related to the miR-17∼92 cluster [260]. miR-92, and to a lesser extent miR-19, targets the Bim 3′-UTR in reporter assays in HeLa cells [263]. Genetically engineered mice with higher expression of miR-17∼92 in lymphocytes developed lymphoproliferative disease and autoimmunity and died prematurely [263]. Lymphocytes from these mice showed more proliferation and less activation-induced cell death [263]. In contrast, mice deficient for miR-17∼92 die shortly after birth with lung hypoplasia and a ventricular septal defect as a result of increased Bim expression [264]. miR-17 and the miR-17∼92 cluster have also been shown to cause resistance of pediatric acute lymphoblastic leukemia to glucocorticoid-induced apoptosis through prevention of Bim expression [265, 266]. Dexamethasone treatment led to reduced expression of the miR-17∼92 cluster, with concomitant upregulation of Bim and apoptotic sensitization [265, 266]. miR-92a promotes glioma cell survival through repression of Bim [267]. miR-20, miR-92 and miR-302 are important for epiblast stem cell survival through repression of Bim [268]. Bim knockout rescued the cell death phenotype in epiblasts of Dicer^−/−^ embryos [268].

miR-25 of the miR-106b∼25 cluster prevents Bim expression in human ovarian cancer cells, promoting their survival [269]. miR-25 also suppresses Bim expression in gastric cancer [175]. The miR-106b∼25 polycistron is activated by genomic amplification and is potentially involved in esophageal neoplastic progression [270]. Repression of the miR-106b∼25 cluster by PKR-like endoplasmic reticulum kinase (Perk) was required for ER stress-induced apoptosis that is mediated by Bim [271]. The HDAC inhibitor trichostatin suppressed miR-106b∼93∼25 expression through downregulation of c-Myc, thereby inducing apoptosis in human endometrial cancer cells [272]. Besides c-Myc, E2F1 positively regulates the expression of the intronic microRNAs 106b∼93∼25 [175]. Of note, *myc* is a direct target gene of E2F1 [273]. Overexpression of the miR-106b∼25 cluster in gastric cancer prevented TGFβ-induced Bim expression and apoptosis [175]. Further studies showed that miR-25, but not miR-106b or miR-93, was responsible for the downregulation of Bim [175]. microRNAs 106b and 93, however, prevent E2F1 expression, forming a negative feedback loop [175].

##### 3.2.2.3. miR-148a

In repeatedly activated T helper type 1 (Th1) cells, Bim expression is downregulated posttranscriptionally by miR-148a [274]. The expression of miR-148a is induced by the transcription factors T-bet and Twist1, which promotes persistence of antigen-specific Th1 cells in long-lasting, chronic immune reactions [274]. Inhibition of miR-148 increased Bim expression and apoptosis of Th1 cells [274]. miR-148a is expressed at higher levels in T cells from rheumatoid arthritis patients than healthy donors [274]. miR-148a is also involved in the survival of glioblastoma cells through downregulation of Bim [275]. Higher levels of miR-148a expression were a risk indicator for glioblastoma patient survival [275].

##### 3.2.2.4. Other microRNAs regulating Bim expression

Upregulation of miR-32 by 1,25-dihydroxyvitamin D3 in human myeloid leukemia cells leads to targeting of Bim and prevention of arabinocytosine (AraC)-induced apoptosis [276]. miR-32 was also found to target Bim in prostate cancer [277]. miR-301a promotes pancreatic cancer cell proliferation by inhibiting Bim expression [278], while miR-363 supports human glioblastoma stem cell survival for the same reason [279].

NGF increases the expression of miR-221/222 in PC12 cells through the ERK pathway. These microRNAs bind to the 3′-UTR of Bim mRNA, thereby preventing Bim translation [254]. miR-124 that is abundantly expressed in midbrain dopaminergic neurons, was downregulated in a mouse model of Parkinson's disease, accompanied by an increase in Bim expression and death of the neurons [280].

miR-24 was found to suppress cardiomyocyte apoptosis in part through repressing Bim [281]. Forced overexpression of miR-24 in a mouse myocardial infarction model inhibited cardiomyocyte apoptosis, attenuated infarct size and reduced cardiac dysfunction [281].

##### 3.2.2.5. microRNAs affecting Bim-regulating factors

microRNAs that downregulate FoxO3 expression, indirectly affect Bim expression [13]. These include miR-1, miR-27a, miR-96, miR-155, miR-182 and miR-221/222 [13]. For instance, aberrant microRNA-182 expression was associated with glucocorticoid resistance in lymphoblastic malignancies [282]. microRNA-182 reduced FoxO3a expression with consequent reduced Bim expression [282]. miR-155 facilitates lymphoproliferation induced by a mutant version of the adaptor protein Linker for Activation of T cells (LAT) via inhibition of FoxO3-dependent Bim expression [283]. miR-155 reduces the expression of the inositol phosphatase Src homology-2 domain-containing inositol 5-phosphatase 1 (SHIP1) [284], thereby promoting Akt-mediated inactivation of FoxO3 [283].

Reduced expression of miR-101 was associated with overexpression of the Bim transcriptional repressor EZH2 in NSCLC [285]. Overexpression of miR-101 sensitized NSCLC cells to paclitaxel-induced apoptosis through inducing Bim expression [285].

microRNAs targeting Phosphatase and tensin homolog deleted on chromosome 10 (PTEN) enhance Akt signaling, resulting in inhibition of apoptosis and Bim expression. These include miR-17∼92, miR-106∼25, miR-21, miR-26a, miR-29b, miR-212, miR-216a, miR-217 and miR-221/222 [13]. Of note, miR-17∼92 targets both PTEN and Bim [263], making it a potential therapeutic target for increasing the response of anti-cancer drugs. In lymphoid malignancies, glucocorticoids repress miR-17∼92, leading to increased Bim expression and drug response [266]. miR-221/222, which is frequently upregulated in cancer [286], targets Bim, PTEN and FoxO3, thus fortifying apoptotic resistance.

microRNAs that target the transcription of anti-apoptotic proteins, also indirectly affect the pro-apoptotic function of Bim. Overexpression of Bcl-2 is common in B cell chronic lymphocytic leukemia (B-CLL) due to loss or downregulation of the human chromosome 13q14 locus, which harbors the miR-15a and miR-16-1 cluster [287]. miR-34a, miR-125b and miR-181c may also target Bcl-2 [13, 288]. Mcl-1 is targeted by miR-29a [289], miR-218 [290], miR-101 [291] and miR-193b [292]. MicroRNAs regulating Fbw7, such as miR-223, miR-27a, miR-25 and miR-129-5p [293] might also indirectly affect Bim-dependent apoptosis through Fbw7-mediated regulation of c-Myc, Mcl-1, Notch1 and c-Jun [293, 294].

### 3.3. Post-Translational Regulation

#### 3.3.1. Regulation of Bim Activity by Protein Kinases

The phosphorylation status of Bim controls its pro-apoptotic activity and stability [7, 25, 295]. Especially, the MAP kinases are involved in the regulation of the pro-apoptotic activity of Bim [25]. In general, phosphorylation of Bim by ERK1/2 leads to the degradation of Bim through the ubiquitin-proteasome pathway, while its phosphorylation by JNK or p38 increases its activity. The Akt/mTOR pathway indirectly affects Bim through phosphorylation and inactivation of the FoxO transcription factors and upregulation of the anti-apoptotic Mcl-1 protein. Thus, receptor signaling or oncogenic addictions activating ERK, Akt and/or mTOR signaling pathways confer apoptotic resistance through reducing Bim expression or antagonizing its activity.

Mutation studies showed that mutation of the phosphorylation site Thr112 in mouse Bim caused decreased binding of Bim to the anti-apoptotic protein Bcl-2 and increased cell survival [295]. However, mutation of the phosphorylation sites Ser55, Ser65, and Ser73 in mouse Bim caused increased apoptosis because of reduced proteasomal degradation of Bim [295]. The amino acid positions differ somewhat between human and mouse Bim (Figure 7), so we have adapted in the text the numbers described in the cited references.

##### 3.3.1.1. ERK1/2

Phosphorylation of BIM_EL_ by ERK1/2 at Ser69 in human or at Ser65 in mouse leads to K48-linked ubiquitin-dependent 26S proteasome-mediated degradation of Bim_EL_ [295-298]. Bim_EL_ can also be degraded by the 20S proteasome in the absence of poly-ubiquitination, as shown by mutating the only two lysine residues Lys3 and Lys108 in rat Bim [298]. This might be related to the fact that Bim_EL_ is an intrinsically unstructured protein [299]. The degradation in absence of poly-ubiquitination is prevented when Bim_EL_ is bound to Mcl-1 [298]. Since exon E2B encodes the ERK1/2-docking domain and ERK1/2 phosphorylation sites, only Bim_EL_, but not Bim_L_ or Bim_S_, is subject to phosphorylation by the MEK/ERK pathway [296, 300]. In addition, ERK phosphorylation of mouse Bim at Ser65 prevents its binding to Bax [296, 300], and leads to its dissociation from Mcl-1 and Bcl-xL [301, 302]. Binding of Bim to Mcl-1 stabilizes the latter [303]. This is in contrast to the binding of Noxa to Mcl-1 that promotes Mcl-1 degradation [303]. Thus, Bim dissociation from Mcl-1 leads to destabilization of both the pro-apoptotic Bim and the anti-apoptotic Mcl-1, the net effect determining the cell fate.

The hematopoietic survival factor Interleukin-3 (IL-3) induces ERK-mediated phosphorylation of mouse Bim on three serine residues (Ser55, Ser65 and Ser100) that prevents Bim from interacting with Bax [7]. Following IL-3 withdrawal, only the non-phosphorylated form of Bim interacts with Bax [7]. Survival is often promoted in tyrosine kinase-driven cancers such as CML and EGFR NSLCL, through repression of Bim transcription and through targeting the Bim protein for proteasomal degradation following Mitogen-activated protein kinase 1 (MAPK1/ERK2)-dependent phosphorylation [304-308]. Targeting EGFR using kinase inhibitors (e.g., erlotinib and gefitinib) led to suppression of PI3K/mTORC and MEK/ERK signaling, followed by an increase in Bim expression [307, 309, 310] and a decrease in Mcl-1 [310]. NGF induces MEK/MAPK-mediated phosphorylation of rat Bim_EL_ at Ser109 and Thr110 in sympathetic neurons [311].

Dehan et al. [312] observed that ERK1/2 co-operates with ribosomal S6 kinase (RSK) to phosphorylate Bim_EL_, allowing binding of the F-box proteins β-TrCP1/2, which promotes BIM_EL_ poly-ubiquitination. Silencing of either β-TrCP or RSK1/2 resulted in Bim_EL_-mediated apoptosis of both gefitinib-sensitive and gefitinib-insensitive NSCLC cells [312]. Phosphorylation of Ser69 of human Bim_EL_ by ERK1/2 promoted cytokine-induced phosphorylation on Ser93, Ser94 and Ser98, that was required for the binding of Bim_EL_ with β-TrCP1 [312]. RSK1 and RSK2 were responsible for the phosphorylation on Ser93/94/98 [312].

Elevated expression of urokinase plasminogen activator (uPA) in EGFR-positive glioblastoma cells leads to apoptosis resistance to EGFR tyrosine kinase inhibitors through ERK1/2-dependent repression of Bim expression [313]. Tyrosine kinase inhibitor-resistant glioblastomas can be resensitized to EGFR tyrosine kinase inhibitors by pharmacologic inhibition of MEK or a BH3 mimetic drug to replace Bim function [313].

ERK1/2 inhibition leads to Bim_EL_ stabilization and increased tumor cell death [314, 315]. Similarly, MEK inhibitors could potentiate dexamethasone lethality of acute lymphoblastic leukemia cells through upregulating Bim expression [316]. Bim accumulated by this treatment interacted with Bcl-xL and Mcl-1, resulting in the release of Bak [316]. Combined treatment of inhibitors of the PI3K/Akt and MEK/ERK1/2 pathways led to Bim-dependent leukemia cell death [317]. The pineal gland hormone melatonin may induce apoptosis of malignant cells by inhibiting Bim degradation by the proteasome, besides inducing SP-1-, FoxO3a-, and E2F1-dependent transcriptional activation of Bim [318-320].

##### 3.3.1.2. JNK

In contrast to ERK phosphorylation that usually leads to Bim degradation, JNK phosphorylation of Bim that occurs, for instance, after trophic factor deprivation, leads to phosphorylation of mouse Bim_EL_ at Ser65 that potentiates its pro-apoptotic activity [156]. Another study showed that following transient focal cerebral ischemia, Bim_L_ is phosphorylated by JNK, enhancing the interaction between Bim_L_ and Bax [321]. The Bax translocation from cytosol to mitochondria was dependent on JNK activity [321]. JNK also phosphorylates Thr112 in mouse Bim_EL_[295, 322] that lies within the dynein binding motif (aa 107-112), leading to the dissociation of Bim_EL_ from the microtubules. UV exposure of 293 human embryonic kidney (HEK) cells led to JNK-mediated phosphorylation of Bim_L_ at Thr56 [90].

The controversy that ERK promotes survival while JNK triggers apoptosis by phosphorylating the same site (Ser65 in mice/Ser69 in human) in Bim_EL_ [25], might be explained by requirement of additional modifications of Bim that affect its activity. As described above, ERK1/2 phosphorylates Ser55 and Ser100 in addition to Ser65, making a docking site for RSK1/2, while JNK phosphorylates Thr112 in addition to Ser65. Becker and Bonni [323] elegantly showed that the prolyl isomerase Pin1 interacts with Ser65-phosphorylated Bim_EL_ in neurons. Pin1 is enriched at the mitochondrial membrane in neurons, where it forms a physical complex with the neuron-specific JNK scaffold protein JIP3 [323]. Activation of JNK signaling induces the dissociation of Pin1 from JIP3 and concomitantly promotes Pin1 binding to phosphorylated Bim_EL_. The interaction of Pin1 with phosphorylated Bim_EL_ stabilizes Bim_EL_ and thereby activates neuronal apoptosis [323]. It could also be a cell-specific phenomenon. Most of the studies showed JNK-mediated phosphorylation of Bim_EL_ in neurons [156, 324], while ERK-mediated phosphorylation is mainly detected in non-neuronal cells [297, 300, 325].

In the T cell acute lymphoblastic leukemia (T-ALL) cell line Sup-T, JNK-mediated phosphorylation of Bim promotes proteasomal Bim degradation [326]. Pretreatment of these T-ALL cells with a specific JNK inhibitor, SP600125, increased the Bim_EL_ level and sensitized the cells to etoposide-induced apoptosis [326]. This may also explain why SP600125 sensitizes glucocorticoid-resistant lymphoid cells to glucocorticoid-induced apoptosis [63]. Thus, the outcome of JNK phosphorylation of Bim seems to be cell-dependent.

##### 3.3.1.3. p38 MAPK

Phosphorylation of Bim_EL_ at Ser65 by p38 increased its pro-apoptotic activity [327]. Sodium arsenite-induced apoptosis in PC12 cells may be due to the direct phosphorylation of Bim_EL_ at Ser65 by p38 [327]. p38 may indirectly increase Bim transcription through positive regulation of FoxO3a [139], Runx2 and c-Jun [159].

##### 3.3.1.4. Akt

Bim transcription is downregulated upon activation of Akt through Akt-mediated phosphorylation and inactivation of FoxO3a [122]. Akt phosphorylates FoxO3a at Thr32, Ser253 and Ser315, leading to its cytoplasmic sequestration by 14-3-3 proteins, thus preventing its nuclear translocation [328]. In addition, Akt phosphorylates Mst1 on Thr387, preventing its kinase activity on FoxO3 [329]. Mst1 mediates oxidative stress-induced neuronal apoptosis by phosphorylating FoxO3 on Ser207 that leads to disruption of its association with 14-3-3 and triggers its nuclear translocation [330]. c-Abl phosphorylates Mst1 at Tyr433 in response to oxidative stress in neuronal cells, leading to its stabilization and activation of FoxO3-mediated Bim expression [331]. In contrast, the Bcr-Abl phusion protein in CML activates Akt [332] through Ubiquitin-specific-processing protease 7 (USP7)-mediated nuclear exclusion of PTEN [333] and CK2-mediated phosphorylation and inactivation of PTEN [334].

In multiple myeloma, Bim expression is downregulated by IL-6 and adhesion to fibronectin [86, 335] that might be related to Akt activation. Qi et al. [336] provided evidence that Ser87 of Bim_EL_ could be phosphorylated by Akt following IL-3-stimulation of Ba/F3 B cells, leading to Bim_EL_ binding to 14-3-3 proteins and attenuation of its pro-apoptotic function.

The oncogenic enzyme Sphingosine kinase 1 (SphK1) that phosphorylates sphingosine to sphingosine-1-phosphate (S1P), is overexpressed in some types of cancer including glioma and gastric carcinoma, leading to apoptotic resistance. This kinase downregulates Bim expression through activation of the Akt pathway and inhibition of FoxO3a [337, 338]. Expression of the sphingosine-1-phosphate receptor S1P1 renders CCL39 lung fibroblasts resistant to apoptosis following growth factor withdrawal, which was associated with attenuated accumulation of Bim [339].

BMCC1 (BNIP2 and Cdc42GAP homology (BCH) motif-containing molecule at the carboxyl-terminal region 1), which is highly expressed in neuronal and epithelial tissues as well as in favorable neuroblastoma, promotes apoptosis by suppressing the PDK1/Akt pathway leading to increased FoxO3a-induced Bim transcription [340]. The BNIP2 homology region of BMCC1 may also interact with Bcl-2 [340].

##### 3.3.1.5. Protein Kinase A

Treatment of T lymphoma cells with the Protein kinase A (PKA) agonist 8-CPT-cAMP led to the phosphorylation of cAMP response element-binding protein (CREB) and induction of Bim, followed by apoptosis [341]. The cytotoxic effect of 8-CPT-cAMP was dependent on PKA and Bim [342]. The PRKAR1A regulatory subunit of cyclin-dependent PKA was found to interact with Bim_EL_ [343]. Phosphorylation of mouse Bim_EL_ at Ser83 (corresponding to Ser87 in human) by PKA leads to stabilization of the Bim protein and induction of apoptosis [343]. Of note, Ser83 resides within the D-box consensus sequence recognized by the Anaphase promoting complex (APC^cdc20^) E3 ligase [344] (Figure 7, and see Section 3.3.2).

#### 3.3.2. Regulation of Bim Expression during Mitosis

Bim_EL_ was found to be regulated during mitosis by Aurora A kinase and PP2A [345]. Bim_EL_ is phosphorylated at the β-TrCP1 phosphodegron Ser93/94/98 by Aurora A early in mitosis, and dephosphorylated by PP2A at the same residues after mitotic exit [345]. The Aurora A-mediated phosphorylation of Bim_EL_ leads to its interaction with β-TrCP1, resulting in the ubiquitination and degradation of Bim_EL_ [345]. Inhibition of either Cdk1 or Aurora A prevented the phosphorylation of Ser93/94/98, suggesting a cross-talk between these two kinases [345]. In accordance with this report, Mac Fhearraigh et al. [346] found that Bim undergoes transient phosphorylation during normal mitosis in K562 cells. The transition of K562 cells from mitosis to G1 resulted in the loss of Bim_EL_ and Bim_L_ phosphorylation. These authors identified the Cdk1/Cyclin B1 complex to be involved in Bim phosphorylation [346]. Also, Gilley et al. [347] observed that Cdk1/Cyclin B1 is involved in mitotic phosphorylation of Bim_EL_, which drives its polyubiquitination and proteasome-dependent degradation. A direct interaction between Cyclin B1 and Bim was observed in mitotic extracts [346, 347]. Bim_EL_ and Bim_L_ are also phosphorylated in mitosis by an MEK/ERK-dependent mechanism downstream to basic fibroblast growth factor (bFGF) signaling [348]. Phosphorylated Bim might facilitate the ordered execution of mitosis in virtue of its reduced pro-apoptotic activity [348].

Besides regulating mitotic progression, the E3 ligase APC^Cdc20^ interacts with Bim_EL_ and promotes its proteasomal degradation [344]. The C-terminal WD40 repeats motif of APC^Cdc20^ binds to the D-box consensus region of Bim_EL_ (amino acids 76-91 in human) [344]. Bim abundance is reduced during mitosis when APC^Cdc20^ is most active [344]. Phosphorylation of Bim by ERK/RSK was not required for APC^Cdc20^ to interact with Bim [344]. Knockdown of APC^Cdc20^ sensitized head and neck cancer cells to apoptotic stimuli by a Bim-dependent mechanism [344]. Human adult T cell leukemia cells that have acquired elevated APC^Cdc20^ activity due to the expression of the Tax viral oncoprotein, exhibited reduced Bim expression and resistance to anti-cancer agents [344]. Overexpression of APC^Cdc20^ predicts poor prognosis in primary NSLCL patients [349].

Extended mitotic arrest following treatment of cancer cells with microtubule-targeting agents led to phosphorylation of Bcl-2 and Bcl-x followed by Noxa-dependent Mcl-1 degradation. This enabled Bim-dependent cell death [350]. Under these conditions, Mcl-1 is also degraded by the E3 ubiquitin ligase Fbw7 [107].

## 4. PHYSIOLOGICAL AND PATHOPHYSIOLOGICAL ASPECTS OF BIM

**Figure 8:**
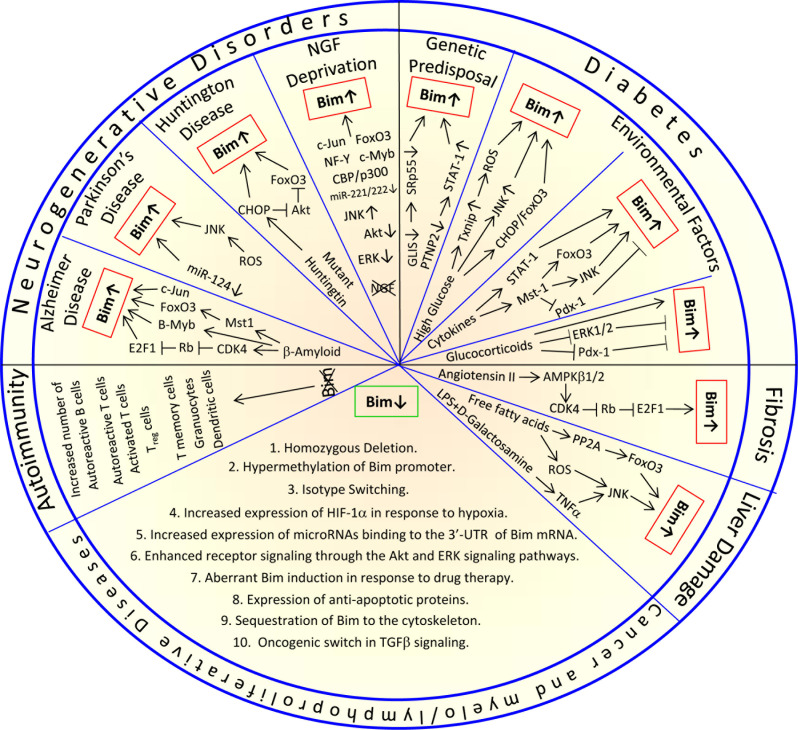
Abberant Bim expression in diseases Elevated Bim expression is associated with neuronal degenerative diseases, diabetes, fibrosis and liver damage, while too low or absent Bim expression is associated with cancer (Section 4). Absent Bim expression in the immune system may lead to autoimmune diseases due to lack of elimination of autoreactive immune cells, but is, in part, compensated by an increase in regulatory T cells. Treatment of neurodegenerative diseases and diabetes should aim in preventing Bim expression, while treatment of cancer should aim in increasing Bim expression. Too dramatic increase in Bim during cancer treatment might thus have adverse effects on the nerve system, liver and endocrine system, that needs to be taken into account. Targeting cancer-specific pro-survival pathways would reduce the adverse effects.

Bim is important for determining the lifespan of myeloid and lymphoid cells, as these cells are increased in numbers in mice lacking Bim [2]. Bim is also required for further homeostatic regulation in situations such as the proper termination of the innate and adaptive immune responses [47]. Alterations in Bim expression are associated with several diseases (Figure 8). Too rapid or too slow elimination of activated T and B cells as a result of too high or too low Bim expression, respectively, leads to chronic infection due to incomplete immune responses or autoimmune diseases. Bim downregulation is involved in cell transformation and reduces the sensitivity of cancerous cells to various chemotherapeutic drugs [121, 246, 304-307]. Increased Bim expression contributes to increased cardiomyocyte and neuronal cell death following ischemia [281, 351], increased β-cell death resulting in diabetes [40] and enhanced neuronal cell death in Alzheimer's disease [352]. Decreased Bim expression confers protection from viral-induced hepatitis and sepsis-related mortality [353, 354]. Below we will discuss these aspects in more detail.

### 4.1. Regulation of Bim by Growth Factor Withdrawal

**Figure 9:**
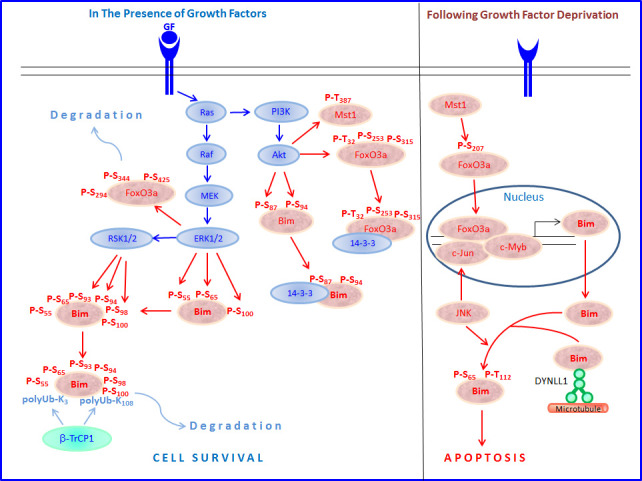
Growth factor-mediated cell survival *versus* apoptosis induction by growth factor deprivation Growth factor receptor activation leads to activation of the Ras-Raf-MEK-ERK1/2 and PI3K-Akt pathways that co-operate in inhibiting Bim activity. ERK1/2-mediated phosphorylation of Bim_EL_ leads to its subsequent phosphorylation by RSK1/2 and ubiquitin-mediated proteasomal degradation. Akt-mediated phosphorylation of Bim leads to its sequestration to 14-3-3 proteins. Akt also inactivates Mst1 and FoxO3a. Following growth factor withdrawal, the lack of activation of the Ras-Raf-MEK-ERK1/2 and PI3K-Akt pathways leads to Mst1 and FoxO3a activation, resulting in the transcriptional upregulation of Bim. In addition, JNK phosphorylates Bim resulting in its activation and apoptosis induction. Inhibition of growth factor receptor signaling (e.g., by EGFR, ALK, c-Met and B-Raf^V600E^ inhibitors) is a major cancer-specific targeting strategy that aims to promote Bim-dependent apoptosis.

Bim expression is induced *de novo* following withdrawal of survival factors (cytokines, growth factors, trophic factors) or serum from primary sympathetic neurons [125, 355], lymphocytes [2, 122, 356], hematopoietic progenitor cells [26], granulocytes [357], mast cells [358, 359], osteoclasts [360], fibroblasts [361] and multiple myeloma [247], while its expression is repressed by the PI3K/Akt or MEK/ERK signaling pathways [117, 122, 359, 361] (Figure 9). In neurons the JNK/c-Jun pathway is required for Bim mRNA expression following withdrawal of NGF [125, 355, 362], but this pathway does not seem to be important for fibroblasts [361]. Bim induction after NGF withdrawal is reduced in sympathetic neurons in mice carrying a mutant c-Jun gene that lacks activating Ser63/Ser73 phosphorylation sites [363]. Further studies showed that induction of Bim in NGF-deprived cells requires expression and activity of Cdk4 and consequent de-repression of E2 promoter binding factor (E2F)-regulated genes including members of the Myb transcription factor family [364]. Mutation in the two Myb binding sites in the *bim* promoter abolished Bim induction following NGF deprivation [364]. In lymphocytes, Bim mRNA expression is promoted by the FoxO3a transcription factor, which is normally repressed by Akt/PKB-mediated phosphorylation. Consequently, inhibition of PI3K or withdrawal of survival factors is sufficient to induce Bim expression in these cells [122].

Addition of growth factors usually leads to downregulation of Bim [26, 311, 360]. IL-3 promotes survival of hematopoietic progenitors through downregulation of Bim [26]. The IL-3-dependent downregulation of Bim is mediated through activation of Raf/MAPK and PI3K/mTOR pathways [26]. IL-3 led to Akt activation and Bim_EL_ phosphorylation at Ser87 in Ba/F3 cells, leading to attenuation of its pro-apoptotic function [336]. IL-7, that is important for T cell survival, inhibits Bim activity [365]. IL-4, that is essential for the activation of mature B cells, prevents upregulation of Bim induced by BCR cross-linking [356]. Similarly, stem cell factor (SCF) promotes mast cell survival via Akt-mediated inactivation of FoxO3a and MEK-regulated phosphorylation of Bim [359]. Erythropoietin induced MEK/ERK-mediated phosphorylation and degradation of Bim that contributes to the survival of erythroid cells [366]. While IL-15 mediates survival of natural killer (NK) cells by Bim downregulation and Mcl-1 upregulation [367], IL-15 leads to upregulation of Bim in T cells through a JAK/STAT5-dependent mechanism [368]. The simultaneous upregulation of Mcl-1 by IL-15 counters the pro-apoptotic effects of Bim, making the T cells prone to apoptosis when the short-lived Mcl-1 is degraded [368]. IGF-1 suppresses Bim expression in multiple myeloma through several mechanisms including activation of the Akt pathway, inactivation of FoxO3, promotion of ERK-induced proteasomal degradation of Bim_EL_ and epigenetic regulation of the *bim* and *foxO3a* promoters [247].

Glucagon-like peptide-1 (GLP-1) protected sympathetic neurons from degeneration and cell death caused by NGF withdrawal through prevention of Bim upregulation [369]. Trophoblast stem cells require fibroblast growth factor-4 (FGF4) for survival and proliferation [370]. The SH2 domain-containing phosphatase 2 (SHP2) was required for FGF4-evoked activation of the Src/Ras/ERK pathway that culminates in phosphorylation and destabilization of Bim [370].

### 4.2. Bim in Alzheimer's disease

#### 4.2.1. Role of Bim in Thrombin-Induced Apoptosis of Neurons

The serine protease thrombin has been shown to be a potent growth factor for a variety of cell types, including vascular smooth muscle cells [371], fibroblasts [372] and some tumor cells [373], besides its central role as a co-ordinator of the blood coagulation cascade [374]. These cellular effects of thrombin on tissues are important for tissue repair following injury. Thrombin acts through protease-activated receptors (PARs), which are members of the G-protein-coupled-receptor superfamily [375]. Thrombin was found to prevent apoptosis of fibroblasts upon serum withdrawal through prevention of Bim upregulation [376]. This effect of thrombin was mediated through the activation of the Raf-MEK-ERK1/2 and PI3K pathways [376]. Thrombin also prevents apoptosis of monocytes [377], osteoblasts [378], myoblasts [379], and astrocytes [380]. However, in motor neurons, thrombin and peptide agonists of the PAR1 receptor promote apoptosis [381, 382]. Cyclin D1, Cdk4 and Bim were shown to be involved in the thrombin-induced apoptosis of cultured cerebral cortical neurons [383]. Here, Cdk4 was responsible for the upregulation of Bim [383]. These data demonstrate that thrombin-induced apoptosis proceeds via cell cycle activation. These authors suggest that cell cycle proteins might be therapeutic targets in diseases such as Alzheimer's disease where thrombin has been implicated [383]. Indeed, Cdk4 inhibitors protected neuronal cells against death induced by NGF deprivation and β-amyloid [384]. This may also explain the anti-apoptotic function of the Cdk4 inhibitor p21 [385].

The coagulation factor FXa, which is a serine protease that plays a role during blood coagulation by converting prothrombin into active thrombin, induced apoptosis of tumor cells of epithelial origin through upregulation of Bim [386]. It is also involved in neuronal death in cerebral ischemia through activation of the JNK/c-Jun pathway [387]. In epithelial tumor cells, FXa activates ERK1/2 and p38 through PAR1 with consequent phosphorylation of the transcription factor CREB [386]. Downregulation of PAR1, Bim, ERK or p38 or the use of a dominant-negative form of CREB was sufficient to prevent FXa-mediated apoptosis of the tumor cells [386]. FXa had no effect on endothelial cells or monocytes, whereas it even enhanced fibroblast survival [386]. Of note, FXa-induced PAR1 activation in fibroblasts led to Bim downregulation [386], demonstrating a cell-dependent effect.

#### 4.2.2. Role of Bim in β-Amyloid-Induced Apoptosis

Examination of human brains of post-mortem Alzheimer's disease patients showed that Bim is upregulated in vulnerable entorhinal cortical neurons, but not cerebellum, a region usually unaffected by the disease [352]. They further demonstrated that Bim is required for β-amyloid-induced neuronal apoptosis [352]. Cdk4 and its downstream effector B-Myb were required for β-amyloid-dependent Bim induction and death in cultured neurons [352]. Cdk4 inhibitors were recently shown to be neuroprotective [384]. Also, FoxO3a and AP-1 (c-Jun/c-Fos) are involved in the β-amyloid-induced upregulation of Bim [388, 389]. The activation of FoxO3a by β-amyloid is in part due to reduced Akt-mediated FoxO3a phosphorylation and increased Mst1-mediated FoxO3a phosphorylation [388]. Estrogen protects against β-amyloid peptide-induced apoptosis by upregulating Bcl-w and downregulating Bim [390].

In addition to parenchymal accumulation and neuronal degeneration in the brain of Alzheimer's disease patients, β-amyloid accumulates in the cerebrovascular wall leading to cerebral amyloid angiopathy. Amyloid-β peptide-induced death in cerebral endothelial cells is preceded by mitochondrial dysfunction and signaling events characteristic of apoptosis. The expression of *Bim*, but not other BH3-only members, was selectively increased in cerebral microvessels isolated from 18-month-old APPsw (Tg2576) mice, a model of cerebral amyloid angiopathy, suggesting a pivotal role for Bim in β-amyloid-induced cerebrovascular degeneration *in vivo* [391]. β-Amyloid increases FoxO3a activity through activation of PP2A that negatively regulates Akt [391]. Suppression of PP2A attenuated Bim expression and cell death in cerebral endothelial cells [391].

### 4.3. Involvement of Bim in Regulating Immune Responses

#### 4.3.1. General Aspects of Bim on Hematopoiesis

Bim is essential for hematopoietic cell homeostasis, thymocyte negative selection and as a barrier against autoimmunity [53]. Bim is required for the deletion of autoreactive T and B cells [36, 46], the death of germinal center-derived memory B cells and antibody-forming cells [392] and the regulation of naïve and memory T cell homeostasis [393]. While the numbes of both CD4^−^8^−^ pro-T cells and mature CD4^+^8^−^ or CD4^−^8^+^ T cells were 2-3-fold higher in Bim KO mice than normal mice, the CD4^+^8^+^ thymocytes population was only half the normal level [2]. Bim^−/−^ mice had increased number of CD25^low^Foxp3^+^CD4^+^ T-regulatory (T_reg_) cells in the thymus and peripheral lymph nodes that were anergic [394]. Bim KO mice show a 2-4 fold increase in B and T cell numbers and suffer from splenomegaly and lymphadenopathy due to accumulation of excess lymphoid and myeloid cells [2]. Bim-deficient mice develop a late-onset autoimmune disease [2].

Also, apoptosis of activated T and B cells and the proper termination of immune responses are dependent on Bim [47, 395]. The Bim KO mice are unable to terminate CD8-driven immune responses after clearing a viral challenge [395] and showed impaired superantigen-induced T cell death [47]. Bcl-2 levels are downregulated in T cells after activation, leading to the sensitization of T cells to Bim following super-antigen injection [47].

Hutcheson et al. [396] studied Bax^−/−^Bim^−/−^ and Bak^−/−^Bim^−/−^ mice, and found that both have dysregulated hematopoiesis. The defects in myeloid and B-lymphoid development were more severe than those found in single KO mice. The thymocytes from these double KO mice were more resistant to apoptotic stimuli mediated by the intrinsic pathway [396]. Further studies by Hutcheson et al. [397] showed that combined deficiency of Bim and Fas resulted in early onset of systemic lupus erythematosus. Hughes et al. [398] observed that Bim^−/−^Fas^lpr/lpr^ mice developed remarkably enhanced and accelerated fatal lymphadenopathy and autoimmunity compared to mice lacking only one of these apoptosis inducers. They further showed that shutdown of an acute T cell response to herpes simplex virus (HSV) involved only Bim with no contribution by Fas, whereas both pathways synergized in killing antigen-stimulated T cells in chronic infection with murine gamma-herpes virus [398]. Similarly, Weant et al. [399] showed that loss of both Bim and Fas function resulted in a synergistic disruption of lymphoid homeostasis, rapid-onset autoimmunity, and organ-specific blocks on contraction of antiviral immune responses. Following lymphocytic choriomeningitis virus (LCMV)-specific immune responses, double-mutant mice had 100-fold more antigen-specific memory CD8^+^ T cells in their lymph nodes than wild-type mice [399]. Bim^−/−^Bmf^−/−^ double deficiency in mice caused more B lymphadenopathy than loss of either BH3-only protein alone [400]. Combined loss of Bim and Bmf favored development of λ light-chain secreting plasma cells [400]. The Bim^−/−^Bmf^−/−^ double knockout mice showed premature lethality due to vasculitits, insulitis and glomerulonephritis [400].

#### 4.3.2. Specific Roles of Bim in T cells

Within the thymus, Bim_L/EL_ expression was observed in the cortex where the double positive thymocytes are located, while Bim was absent from the thymic medulla [6]. Bim is required for the apoptosis of double positive thymocytes induced by high-affinity antigens [401]. In immature murine thymocytes, Bim is associated with mitochondria before stimulation and its level is not increased following CD3ε cross-linking, a treatment stimulating signals through the TCR [402]. However, CD3ε cross-linking led to rapid phosphorylation of Bim_EL_ in CD4^+^CD8^+^ thymocytes [402]. In contrast to TCR signaling, dexamethasone did not lead to Bim phosphorylation in CD4^+^CD8^+^ thymocytes [402]. Bim is overexpressed in pro-apoptotic, pre-TCR-deficient thymocytes [403]. Pre-TCR signaling suppresses Bim expression through PI3K/Akt-mediated inhibition of FoxO3a [403]. Bim is also essential for the deletion of CD4^+^CD8^−^CD24^+^ thymocytes in response to TCR ligation [404]. TCR ligation upregulates Bim expression in thymocytes and promotes the interaction of Bim with Bcl-xL, leading to thymocyte killing [46].

Bim is also required for the activation of autoreactive T cells [41]. Deletion of Bim in hematopoietic cells rendered mice resistant to autoimmune encephalomyelitis and diabetes, and Bim-deficient T cells showed diminished cytokine production. The Bim-deficient T cells showed defective calcium signaling upon T cell receptor activation, that was associated with an increase in the formation of an inhibitory complex containing Bcl-2 and the inositol triphosphate receptor (IP3R). Thus, in addition to mediating the death of auto-reactive T cells, Bim controls T cell activation through the inositol triphosphate receptor (IP3R)/calcium/nuclear factor of activated T cells (NFAT) pathway [41]. This function may explain why Bim-deficient mice do not reject their own organs despite lacking thymic negative selection [41].

Bim is required for negative selection of thymocytes recognizing tissue-restricted (TRA), but not ubiquitous (UbA), self-antigens [401, 405, 406]. In polyclonal Bim KO mice, there is an increase in anergic phenotype CD4^+^ T cells, which may indicate an increase in self-specificities due to a block of clonal deletion [405]. Also, Bim deficiency in Vβ5 transgenic mice led to impaired peripheral deletion of CD4^+^ T cells, resulting in more Recombination activating gene (RAG)-expressing, revising CD4^+^ T cells [407].

Selective clonal deletion of the CD4^+^ T cell compartment during the transition from effector to memory is accompanied by enhanced expression of Bim. Bim deficiency enables the survival of poorly functional Th1 responders that are normally eliminated during contraction [408]. Bim^−/−^ CD4^+^ memory T cells showed deficient effector functions, poor sensitivity to antigen and an inability to respond to secondary challenge [408]. Thus, Bim plays a key role in shaping the CD4^+^ memory T cell repertoire, ensuring the emergence of highly functional CD4^+^ memory T cells and the elimination of Th1 effector cells with sub-optimal function [408].

Bim was also found to be involved in the apoptosis of effector T cells following interaction of B7-H1 (PD-L1) with its receptor PD-1 [409]. B7-H1 is expressed on primary and metastatic tumor cells as well as antigen-presenting cells and exerts immunosuppressive functions on CD8^+^ T cells. Interruption of the PD-L1/PD-1 interaction using blocking antibodies to either of the two molecules has proven effective in increasing the anti-tumor immune responses [410]. More memory CD8^+^ T cells were generated in B7-H1-deficient mice following immunization as compared to wild-type mice [409]. At the peak of the expansion phase, CD8^+^ T cells expressed lower levels of Bim in the B7-H1-deficient mice than wild-type mice [409]. Bim is a key regulator of T cell apoptosis during the contraction phase of CD8^+^ T cell response [399]. Thus, B7-H1 negatively regulates CD8^+^ T cell memory by enhancing the depletion of effector CD8^+^ T cells through upregulation of Bim [409]. Also, Kurtulus et al. [411] observed that Bim controls T cell memory development by limiting the survival of pre-memory effector cells. The absence of Bim increased the effector CD8^+^ T cell population with more memory potential, which was due to increased IL-15-dependent survival of memory precursors [411]. Although Bim was critical to limit survival of killer-cell lectin like receptor G1 (KLRG1)^hi^CD127^lo^ T effector cells, the absence of Bim enriched for KLRG1^lo^CD127^hi^ pre-memory T cells as the response progressed [411].

Also the production of regulatory T cells (T_reg_) was affected by Bim [37-39]. Regulatory T cells accumulate dramatically in aged mice, which is due to reduced Bim expression [38]. Bim expression naturally declines in peripheral wild-type CD4^+^ T cells during aging [412], which is co-incident with the increasing numbers of T_reg_ cells [37]. In the absence of Bim, T_reg_ cells accumulate rapidly, accounting for >25% of the CD4^+^ T cell population by 6 months of age [38]. The increased T_reg_ population in Bim KO mice may explain, at least in part, why these mice do not develop aggressive T cell-mediated autoimmune disease.

The preferential accumulation of T_reg_ cells in the absence of Bim might be explained by the rapid peripheral T_reg_ cell turnover that depends on Bim [413] and antagonized by Mcl-1 [39]. Tischner et al. [414] observed that the longer-lived Bim-deficient T_reg_ cells showed reduced suppressive capacity in a model of T cell-driven colitis. However, Reckling et al. [415] noticed increased resistance of Bim^−/−^ mice to persistent Leishmania major infection. The initial parasite growth and lesion development were similar in Bim^−/−^ and wild-type mice after primary Leishmania major infection [415]. At later times after infection, Bim^−/−^ mice had significantly increased Leishmania major-specific CD4^+^ T cell responses and were resistant to persistent infection [415].

Retinoic acid inhibits TCR-mediated cell death of thymocytes during negative selection by inhibiting DNA binding of Nur77 and synthesis of Bim [416]. This study showed that anti-CD3- and specific antigen-driven, but not superantigen-mediated apoptosis, involves Bim [416]. Nur77 didn't directly upregulate Bim [416], but could increase apoptosis by antagonizing Bcl-2 [417]. A recent report by Kiss et al. [418] suggests that Nur77 induces STAT-1 that, in turn, enhances Bim expression [418].

Larrubia et al. [419] observed that Bim expression in hepatitis C virus (HCV)-specific CD8^+^ cells was elevated in persistent HCV-infected patients in comparison to those with resolved infection. The CD8^+^ cells from resolved infection showed a PD-1^−^CD127 (IL-7 receptor)^+^ phenotype and proliferated after stimulation, while the CD8^+^ cells from persistent infection patients showed a PD-1^+^CD127^−^Bim^+^ phenotype with impaired proliferation after stimulation [419]. Blocking apoptosis and the PD-1/PD-L1 pathway enhanced their reactivity *in vitro* [419].

CD137L (4-1BBL), a stimulator of anti-viral CD8 T cell responses, led to TNFR-associated factor 1 (TRAF-1)-dependent Bim downregulation in activated virus-specific CD8^+^ T cells, resulting in enhanced T cell expansion [420]. This study confirms the studies showing that CD137L recruits TRAF1 during signaling in T cells [421] and TRAF-1 reduces Bim expression in activated T cells [422]. TRAF1-deficient, antigen-activated T cells accumulated higher levels of Bim, particularly the Bim_S_ isoform, thereby showing reduced CD8 T cell response to influenza virus [422]. Bim downregulation led to increased memory T cell survival in TRAF1-deficient mice [422]. Agonistic antibodies to CD137 (e.g., Urelumab) promoted survival and activation of specific T cells, and increased the immune responses against melanoma [423].

#### 4.3.2. Specific Roles of Bim in B cells

Bim-deficient B lymphocytes are refractory to apoptosis induced by ligation of B cell antigen receptor (BCR) [36]. BCR ligation promoted interaction of Bim with Bcl-2, inhibiting its survival function [36]. B cell activating factor belonging to the tumor necrosis factor family (BAFF) and A proliferation-inducing ligand (APRIL) block BCR-induced apoptosis by downregulating Bim expression [424]. BAFF induced ERK-mediated Bim phosphorylation and inhibited BCR-induced association of Bim with Bcl-2 [424]. Autoreactive B cells appear to depend on increased BAFF signaling for survival to counteract self-antigen-driven increases in Bim levels [425]. Normal B cells require BAFF both for differentiation and survival, while Bim-deficient B cells require BAFF only for differentiation [426]. IL-4 showed a unique protective effect against anti-IgM apoptotic signals on transitional B cell checkpoints by reducing Bim expression [356]. IL-4 and BAFF synergized to promote B cell maturation [356].

Although transitional immature type 1 (T1) B cell numbers are normal in Bim KO mice, T2 and follicular mature B cells are elevated and marginal zone B cells are reduced [424]. Bim loss prevented deletion of autoreactive B cells induced by soluble self-antigen and promoted accumulation of self-reactive B cells developing in the presence of membrane-bound self-antigen [36]. Bim-deficient B cells were protected *in vivo* from superantigen-induced death and underwent persistent massive supraclonal expansion without functional impairment [427]. The microbial virulence factor protein A of *Staphylococcus aureus* induced specifically the Bim_S_ isoform in B cells [427].

Bim expression is required for cross-linked anti-μ antibody-mediated apoptosis in various Burkitt's lymphoma cell lines, whereas it is not required for apoptosis triggered by soluble anti-μ antibody [83]. Following anti-μ antibody stimulation, Bim_EL_ is phosphorylated by ERK followed by proteasome-dependent degradation, a process that occurs concomitantly with *de novo* synthesis of Bim_L_ and Bim_S_ that were responsible for the pro-apoptotic effect through direct interaction with Bax [83]. This shift in Bim isoform expression was not observed when the Burkitt's lymphoma cells were exposed to TRAIL or TGFβ [83].

Craxton et al. [428] presented data suggesting a role for of Bim in regulating BCR-induced entry of B cells into the cell cycle. Bim KO B cells had reduced cell division compared to wild-type B cells in response to BCR, Toll-like receptor 3 (TLR3) or TLR4 signaling, whereas Bim deficiency did not affect TLR9-induced B cell division [428]. Cell cycle progression in BCR- and lipopolysaccharide (LPS)-stimulated Bim KO B cells was blocked at the G_0_/G_1_ stage [428]. BCR-induced p130 degradation and pRb hyperphosphorylation on Ser807/811, which are critical for G_1_ entry, were reduced in Bim KO compared to wild-type B cells [428].

B cell Sialic acid-binding immunoglobulin-type lectins (Siglecs) mediate tolerance to cell surface antigens by initiating an inhibitory signal that culminates in elimination of antigen-reactive B cells. CD22 and Siglec-G are recruited to the immunological synapse by sialic acid ligands on the antigen-binding cells, producing a tolerogenic signal involving Lyn and Bim that promotes deletion of the B cells and failure of mice to develop antibodies to the antigen upon subsequent challenge [429]. This mechanism may be involved in the induction of humoral tolerance in recipients by donor-specific transfusion following organ transplantation [429].

#### 4.3.3. Role of Bim in Macrophages and Dendritic cells

Bim is phosphorylated and upregulated during Toll-like receptor (TLR) stimulation of macrophages [430]. Inhibition of the MAPK p38 reduced both upregulation of Bim and apoptosis [430]. Bim upregulation was induced by LPS that stimulates TLR4, the bacterial lipopeptide Pam_3_Cys that stimulates TLR2, and CpG oligodeoxynucleotide that stimulates TLR9 [430]. It should be noted that upregulation of Bim per se was insufficient in inducing apoptosis, that required an additional signal [430]. The adaptor protein MyD88 was both necessary and sufficient for the induction of Bim [430]. FoxO3a seems to be involved in Bim induction, as it translocates into the nucleus upon TLR stimulation [430]. Of note, Bim^−/−^ macrophages were resistant to *E. coli* phagocytosis-induced apoptosis [430]. TLR stimulation might also promote Bim degradation through ERK-mediated phosphorylation of Bim at Ser55, Ser65 and Ser100 [431].

Bim KO mice had an increased number of dendritic cells [432]. Bim is expressed at low levels in dendritic cells, but is significantly upregulated by signaling from CD40 or TLRs [432]. Simultaneously with the upregulation of Bim, the anti-apoptotic Mcl-1 is also upregulated by TLR signaling, that antagonizes the pro-apoptotic function of Bim during the early stages of dendritic cell activation [432]. GM-CSF treatment of dendritic cells led to reduced Bim expression and increased dendritic cell survival [432]. Bim-deficient dendritic cells showed decreased spontaneous cell death and induced more robust T cell activation, thus being more immunogenic [432]. Adoptive transfer of Bim-deficient dendritic cells led to the induction of autoantibody production that may contribute to the spontaneous systemic autoimmunity observed in Bim KO mice [432].

#### 4.3.4. Role of Bim in Granulocytes

Myeloid cell life span is substantially increased in Bim KO mice, and blood neutrophil counts in these animals are increased by a factor of 2.5 [2]. Both immature and mature neutrophils express the three major isoforms of Bim: Bim_EL_, Bim_L_ and Bim_S_ [433]. Bim deficiency, like Bcl-2 overexpression, renders granulocytes resistant to cytokine withdrawal and cytotoxic drugs such as etoposide and paclitaxel, but does not have any impact on FasL-induced apoptosis [357].

The neutrophil survival factor GM-CSF does not only prevent the normal time-dependent loss of the anti-apoptotic proteins Mcl-1 and Bcl2A1 in neutrophils important for their survival, but also increases Bim transcription and Bim_EL_ expression by a mechanism dependent on NFκB [434]. It is possible that neutrophils can tolerate the increase in Bim expression because of the corresponding increase in anti-apoptotic Bcl-2 members [434]. Increased Bim mRNA expression was observed in neutrophils in lung lavage from patients with ventilator-associated pneumonia [434] and in neutrophils from blood of septic patients [433]. The paradoxical increase in Bim expression by the neutrophil survival factor may function to facilitate rapid apoptosis at the termination of inflammation [434]. *In vitro*, GM-CSF primes neutrophils for TNFα-mediated killing [434]. A similar pro-apoptotic counter-regulation of GM-CSF on neutrophils was observed by Andina et al. [433]. A lack of Bim resulted in a much higher efficacy of the survival cytokines IL-3 and GM-CSF to block neutrophil apoptosis [433]. Bauer et al. [435] showed a similar paradox that LPS stimulation of mouse bone marrow neutrophils reduced spontaneous apoptosis, but at the same time increased Bim expression.

#### 4.3.5. Role of Bim in NK cells

Natural killer (NK) cells undergo antigen-driven expansion to become memory cells after mouse cytomegalovirus (MCMV) infection. Bim^−/−^Ly49H^+^ NK cells expanded normally during the early infection phase, but showed reduced contraction after the expansion phase leading to higher NK cell numbers than wild-type cells [436]. The inability to reduce the effector pool leads to larger Bim^−/−^ NK memory subsets, which displays a less mature phenotype (CD11b^lo^, CD27^+^) and lower levels of NK cell memory-associated markers KLGR1 and Ly6C [436]. Bim^−/−^ memory NK cells showed a reduced response to the MCMV-encoded ligand m157-mediated stimulation and were less effective than wild-type NK cells in protecting against MCMV infection [436].

#### 4.3.6. Role of Bim in Mast Cells

Bcl-xL and Bim were both induced upon FcεRI activation of mast cells [358]. Bim deficiency prevented cytokine withdrawal-induced mast cell apoptosis, suggesting for an essential role of Bim in this cell death process [358]. The Kit receptor tyrosine kinase signaling via SHP2 and ERK was found to be important for the downregulation of Bim required for mast cell survival [437]. SHP2^−/−^ mast cells failed to mount an IgE-mediated late phase cutaneous response [437]. PGE_2_ increases mast cell death during cytokine deprivation by augmenting Bim expression [438]. Influenza A virus induces apoptosis of mast cells that concurs with upregulation of Bim [439].

#### 4.3.7. Role of Bim in Megakaryopoiesis

Bim-deficient CD34^−^/c-kit^+^/Sca-1^+^/Lin^−^ hematopoietic stem cells and megakaryocytes are resistant to apoptosis induced by cytokine depletion [440]. Platelet recovery after 5-fluorouracil-induced thrombocytopenia is delayed in Bim KO mice, which is reflected in reduced number of megakaryocytes [440]. Bim-deficient c-Kit^+^/Lin^−^ progenitor cells poorly proliferate and differentiate into CD41^+^ cells in response to thrombopoietin, but once differentiated into megakaryocytes, these cells mature normally. The transition from G_1_ to S phase is delayed in Bim-deficient hematopoietic stem cells, suggesting a role for Bim in regulating cell cycle progression in hematopoietic progenitors during megakaryopoiesis [440].

### 4.4. Involvement of Bim in Neuronal Differentiation and Apoptosis

Bim and Puma have been shown to be involved in the death of cells that have been newly generated during neurogenesis in the adult hippocampus [441]. Bim-deficient mice showed similar rates of precursor cell proliferation in the mouse hippocampus as wild-type mice, but the survival of adult-born cells in the dentate gyrus was increased [441]. There was no change in the early markers of neuronal differentiation, while the differentiation of newly generated cells into a neuronal phenotype was accelerated in the Bim-deficient mice [441]. These findings suggest that besides a role in regulating neuronal progenitor cell survival, Bim may inhibit neuronal differentiation [441]. Also the anti-apoptotic Bcl-2 has been implicated in neuronal differentiation [442] and promotes axonal outgrowth of retinal ganglion cells [443]. As Bim binds Bcl-2, it could be that Bim determines the available Bcl-2.

Cellular stresses resulting from extracellular stimulation by H_2_O_2_ or β-amyloid promote hyperactivation of cyclin-dependent kinase 5 (Cdk5) [444]. Cdk5 prevents cell death of postmitotic cortical neurons through phosphorylation of FoxO1 at Ser249, thus preventing FoxO1-mediated upregulation of Bim [444].

Dysfunction of mitochondrial complex I associated with Parkinson's disease leads to degeneration of dopaminergic neurons of the substantia nigra pars compacta (SNpc) through a mechanism involving JNK-dependent activation of Bim with consequent Bax translocation to the mitochondria and apoptosis [445]. Analysis of post-mortem human brain samples from Parkinson's disease patients has shown elevated JNK activation [446].

Activation of Notch contributes to ischemic neuronal cell death [447]. Inhibition of the Notch-activating enzyme γ-secretase protected against ischemic neuronal cell death by reducing Bim expression [447]. Treatment of mice with the γ-secretase inhibitor compound E reduced infarct size and improved functional outcome in a model of focal ischemic stroke [447]. The upregulation of Bim by active Notch in neuronal cells was mediated by NFκB [447]. Of note, constitutively active Notch1 downregulates Bim expression in other cell types (e.g., T cell acute lymphoblastic leukemia) through induction of c-Myc and repression of PTEN that leads to the activation of the PI3K/Akt/mTOR pathway [294]. The Notch1 transcriptional function in T malignancies and pancreatic cancer was supported by Bcl-2 that fortified drug resistance [199, 448].

### 4.5. Involvement of Bim in Diabetes

Bim contributes to cytokine- [183, 188], virus- [449] and high glucose-induced [450, 451] pancreatic β-cell apoptosis. Bim can be regulated by pro-inflammatory cytokines at the transcriptional level through STAT1 [183] and by phosphorylation [188]. Puma and Bax are also involved in high glucose-induced apoptosis of islet cells [450, 451]. Loss of Bid, Noxa or Bak had no impact on glucose-induced apoptosis [451]. Islets deficient in both Bim and Puma, but not Bim or Puma alone, were protected from killing induced by the mitochondrial reactive oxygen species donor rotenone [450].

Non-obese diabetic (NOD) mice that develop spontaneous autoimmune diabetes, show decreased thymic clonal deletion associated with diminished induction of Bim and Nur77 [452]. These mice show defective thymic enrichment of CD4^+^CD25^+^ T_reg_ cells [452]. Bim deficient NOD mice developed less insulitis and were protected from diabetes despite substantial defects in the deletion of autoreactive thymocytes [40]. Bim deficiency didn't impair effector T cell function, but NOD Bim^−/−^ mice had increased numbers of antigen-specific T_reg_ cells both in the thymus and peripheral lymphoid tissues [40]. It is likely that the absence of Bim in the β-cells prevents their cell death, thereby avoiding the trigger of the immune system, which often happens due to cell death-released factors such as High-mobility group box 1 (HMGB1) that activates the receptor for advanced glycation end products (RAGE) and spliceosome-associated protein 130 (SAP130) activating C-type lectin 4e (Clec4e) [453].

The Bim gene Bcl2l11 lies within the Idd13 diabetes susceptibility locus [40, 452]. As described in Sections 3.1.3 and 3.1.1.7, diabetes-related GLIS deficiency leads to preferential upregulation of the Bim_S_ isoform [233], and diabetes-related PTPN2 deficiency leads to increased activation of STAT-1 resulting in increased Bim transcription [183]. Also, other candidate risk genes for type 1 diabetes have been described that protects β-cells from apoptosis through prevention of Bim upregulation. For instance, deficiency in the basic leucine zipper transcription factor 2 (BACH2) leads to increased phosporylation of JNK1 by mitogen-activated protein kinase kinase 7 (MKK7) and downregulation of PTPN2. These changes, in turn, led to increased Bim expression [454]. The candidate gene cathepsin H prevents β-cell apoptosis by suppressing JNK and p38 signaling and reducing Bim expression [455]. Pro-inflammatory cytokines decreased the expression of cathepsin H in human islets, and overexpression of cathepsin H protected β-cells against cytokine-induced apoptosis [455].

Islets of human donors with type 2 diabetes had higher mRNA levels of Bim and Puma, suggesting that these pro-apoptotic proteins contribute to β-cell death [450]. High concentrations of glucose led to increased expression of the transcription factor CHOP (C/ERB homologous protein) [450], that co-operates with FoxO3a to regulate Bim and Puma expression [181]. The increased apoptosis of β-cells in pancreatic duodenal homeobox-1 (Pdx1)-haploinsufficient mice could be prevented by simultaneous knockout of either Bim or Puma [456]. Both Bim and Puma expression is upregulated in β-cells following Pdx1 suppression [456]. Similarly, Bim mediates β-cell death upon Insulin receptor substrate 2 (IRS2) deficiency [457]. Dexamethasone induced Pdx1 downregulation and Bim activation in β-cells by a mechanism dependent on glucocorticoid receptor activation, but independent of FoxO1 [458]. Diabetogenic conditions lead to the upregulation of Mst1 in β-cells of both human and mouse islets, resulting in the induction of Bim expression and apoptosis [459]. Mst1 phosphorylates Pdx1 at Thr11, resulting in ubiquitination and degradation of Pdx-1 [459].

### 4.6. Involvement of Bim in Osteoarthritis

Osteoarthritis is a chronic degenerative joint disorder characterized by increased apoptosis of chondrocytes. Bim is upregulated in chondrocytes obtained from the articular cartilage of osteoarthritis patients and in cultured mouse chondrocytes treated with IL-1β [460]. The cytokine IL-1β induces chondrocyte apoptosis through activation of the JNK-c-Jun pathway that leads to induction of Bim expression [460]. Genetic knockdown of Bim reduced chondrocyte apoptosis, suggesting a role for Bim [460].

### 4.7. Involvement of Bim in Rheumatoid Arthritis

The expression of Bim was reduced in macrophages from synovial tissue of patients suffering from rheumatoid arthritis [461]. Macrophages from Bim KO mice displayed elevated expression of inflammation markers and secreted more IL-1β in response to LPS or thioglycollate, suggesting for a role of Bim in limiting the activation of macrophages [461]. A Bim-BH3 mimetic (TAT-BH3) peptide induced apoptosis of myeloid cells and reduced the symptoms of arthritis in mice treated with K/BxN serum [461].

### 4.8. Involvement of Bim in Liver Damage

A fine rheostatic balance between the anti- and pro-apoptotic multidomain Bcl-2 family proteins controls hepatocyte apoptosis in the healthy liver. Bim and Bid are functionally active, but are restrained by the anti-apoptotic Bcl-2 family proteins under physiological conditions [462]. Spontaneous hepatocyte apoptosis in Bcl-xL- or Mcl-1-knockout mice was ameliorated by simultaneous Bim deletion [462]. Hepatocyte apoptosis caused by the BH3 mimetic ABT-737 was completely prevented in Bim/Bid double knockout mice [462]. The Wnt/β-catenin signaling pathway protects mouse liver against oxidative stress-induced apoptosis through SGK1-mediated inhibition of FoxO3, resulting in reduced Bim expression [463]. Acetaminophen (Paracetamol) induced apoptosis of hepatocytes through JNK-mediated upregulation of Bim that was aggravated by simultaneous stimulation by TRAIL [464]. TRAIL-deficient or Bim-deficient mice were protected from acetaminophen-induced liver damage [464]. Similarily, TRAIL enhanced Fas-induced hepatocyte apoptosis through activation of JNK and Bim [465].

Hepatocyte lipoapoptosis caused by exposure of hepatocytes to saturated free fatty acids (e.g., palmitic and stearic acids) is mediated by FoxO3a-dependent Bim induction [466]. Free fatty acids induce the expression of PP2A that is responsible for FoxO3a dephosphorylation and activation [466]. Free fatty acids also activate JNK that increases Bim activity [467]. Knockdown of Bim prevented hepatocyte apoptosis induced by free fatty acids [467]. Fatal hepatitis mediated by TNFα, produced in response to LPS and the transcriptional inhibitor D(+)-galactosamine, could be prevented by the simultaneous deletion of Bid and Bim [468]. Palmitic acid, but not oleic acid, induced the degradation of the Kelch-like ECH-associated protein-1 (Keap-1) in hepatocytes through autophagy in a p62-dependent mechanism [469]. The reduced expression of Keap-1 led to JNK1-dependent upregulation of Bim and Puma and induction of hepatocyte apoptosis [469].

Bim is involved in the elimination of virus-specific T cells that have been activated by hepatocytes following hepatitis B and C virus (HBV/HCV) infections [470]. This leads to peripheral immunological tolerance towards the virus, contraction of the immune response and prevention of immune cell-mediated liver damage [470]. Bim especially promotes the elimination of CD127^low^-expressing virus-specific T cells [471]. CD127^high^ T cells receive survival signals delivered by IL-7, leading to upregulation of Mcl-1 that captures Bim, thereby preventing apoptosis while promoting resolution of the viral infection [472]. Thus, the CD127 phenotype of the virus-specific T cells affects T cell reactivity by regulating apoptosis determined by the Bim/Mcl-1 balance.

Liver-activated CD8^+^ T cells showed increased expression of Bim and caspase 3, making the T cells prone to apoptosis following intrahepatic activation [473]. T cells deficient for Bim survived following intrahepatic activation [473]. HBV-specific CD8^+^ T cells from patients with chronic infection showed higher Bim expression than those with resolved infection [474]. Blocking Bim-mediated apoptosis enhanced the recovery of HBV-specific CD8^+^ T cells [474]. The few surviving HBV-specific CD8^+^ T cells were CD127^hi^ and expressed elevated levels of Mcl-1, suggesting that they were amenable to IL-7-mediated rescue from apoptosis [474].

During persistent lymphocytic choriomeningitis virus (LCMV) infection, Bim was important for the specific elimination of CD8^+^D^b^NP396-404^+^ T cells [475]. Bim played a dual role in the development of LCMV-induced, T cell-mediated hepatitis [353]. Absence of Bim in parenchymal cells attenuated liver damage, while loss of Bim in the lymphoid compartment enhanced hepatitis [353]. In Bim KO mice, the effect of Bim deficiency in the lymphoid compartment was counterbalanced by the reduced sensitivity of Bim KO hepatocytes to T cell-induced apoptosis, resulting in the protection of the mice from hepatitis [353]. The signal adaptor TNFR-associated factor 1 (TRAF1) negatively correlates with Bim and it contributes to CD8 T cell-mediated control of chronic viral infections. 4-1BB (CD137), a TNFR family member implicated in prolonging the survival of activated and memory CD8 T cells, activates ERK through association with TRAF1 and TRAF2, resulting in accelerated degradation of Bim [476]. TRAF1 is specifically lost from virus-specific CD8 T cells during the chronic phase of infection with human immunodeficiency virus (HIV) or LCMV [477]. TGFβ induces the posttranslational loss of TRAF1, whereas IL-7 restores TRAF1 expression [477]. Thus, IL-7 may have therapeutic applications in the treatment of chronic viral illnesses.

### 4.9. Role of Bim in Mammary Lumen Formation

Epithelial cells organize into cyst-like structures that contain a spherical monolayer of cells that enclose a central lumen. Reginato et al. [325] showed a requirement for Bim in selectively triggering apoptosis of the centrally localized acinar cells, leading to controlled lumen formation. In a three-dimensional basement membrane culture model in which mammary epithelial cells form hollow, acinus-like structures, Bim was not detectable during early stages of mammary acinar morphogenesis, but was later highly upregulated in acinar cells [325]. Inhibition of Bim expression by RNA interference transiently blocked luminal apoptosis and delayed lumen formation [325]. Oncogenes that induce acinar luminal filling, such as ErbB2 and v-Src, suppressed Bim expression through ERK-dependent pathway [325]. Bim is also involved in the death of alveolar cells during involution of the mammary gland after cessation of lactation [478].

### 4.10. Radioresistance of Mesenchymal Stromal Cells (MSCs)

Mesenchymal stromal cells (MSCs) are intrinsically resistant to γ-irradiation-induced cell death by expressing high levels of DNA damage response proteins and high levels of Bcl-2 and Bcl-xL together with low Bim expression [479]. This property of MSCs has implications for allogeneic bone-marrow transplantation, graft-versus-host disease and cancer treatment.

### 4.11. Involvement of Bim in Cancer

Reduced Bim expression is a hallmark for carcinogenesis. Tumor cells have evolved different mechanisms to suppress Bim expression and/or activity thereby overcoming the apoptotic barrier that else would have led to their eradication. The efficacy of many anti-cancer drugs depend on Bim, and insufficient Bim induction or Bim function is often an underlying cause of therapy failure. Many cancer cells have developed one or more mechanisms for preventing Bim from acting, intervention of which may result in the reactivation of the apoptotic process. Determination of the specific survival dependency pathway in each cancer case is important for choosing the right targeting drug therapy.

#### 4.11.1. A Tumor Suppressive Function of Bim

Several studies suggest that Bim functions as a tumor suppressor. In mice, inactivation of one allele of Bim accelerates Myc-induced B cell leukemia [480]. In this experimental system, Bim is induced by Myc, and inactivation of one Bim allele was sufficient for Myc-induced tumor development. Whereas the p19^Arf^/p53 pathway is frequently mutated in tumors arising in Bim^+/+^ Eμ-Myc mice, it was unaffected in most Bim-deficient tumors, indicating that Bim reduction is an effective alternative to loss of p53 function [480]. Similarly, Bim deficiency in mice overexpressing the Eμ-vAbl oncogene accelerated the development of plasmacytomas [481]. The v-Abl-expressing plasmacytomas frequently harbor a rearranged c-Myc gene [481]. Another example is the formation and growth of tumors derived from baby mouse kidney epithelial (BMK) cells transformed by E1A and dominant negative p53, that is facilitated by simultaneous Bim deficiency [482], suggesting a role for Bim in preventing epithelial cancer cell formation. These authors [482] also showed that Bim-deficiency led to paclitaxel-resistant tumor cells.

An interesting study by Merino et al. [163] showed that Bim deficiency in PyMT (MMTV-Polyoma middle-T) female mice didn't affect primary breast tumor growth, but rather increased the survival of metastatic cells within the lung. They further showed a correlation between Bim expression and the EMT transcription factor SNAI2 at the proliferative edge of the tumor. Chromatin immunoprecipitation analysis suggests that Bim is a target of SNAI2 [163]. These data suggest a role for Bim in the suppression of breast cancer metastasis.

Bim is frequently eliminated in human cancer, providing a growth advantage to the tumor cells. For instance, homozygous deletions of the Bim locus have been observed in mantel cell lymphomas and methylation of the *bim* promoter has been found in certain Burkitt's lymphoma and diffuse large B cell lymphoma [246, 483]. Bim is downregulated in a subset of colorectal cancers through Cox2/PGE_2_ signaling that activates the c-Raf/MEK/ERK1/2 pathway [484]. Treatment of Cox2-expressing colorectal carcinoma with selective Cox2 inhibitors induced Bim expression [484]. Downregulation of Bim expression was associated with tumor progression towards an anchorage-independent phenotype [484].

When the ERK pathway is constitutively activated in tumor cells, Bim is degraded, and confers chemoresistance. This was demonstrated by Tan et al. [482] where apoptotic sensitivity to paclitaxel could be restored in H-Ras/ERK-dependent tumor cells, by treatment with the proteasome inhibitor bortezomib that promoted Bim-dependent tumor regression [482]. Bortezomib also sensitized prostate and colon cancer cells to TRAIL-mediated cell death through a mechanism dependent on Bik and Bim [485]. Similarly, Bim levels are low in NSCLC cells harboring activating EGFR mutations [306-309]. Inhibition of EGFR tyrosine kinase activity by drugs such as gefitinib, results in Bim_EL_ accumulation and induction of apoptosis.

Metastatic melanomas had lower levels of Bim than dysplastic nevi [486]. Reduced Bim expression was significantly correlated with poor 5-year survival of melanoma patients [486]. The single point mutation V600E in B-Raf, frequently observed in melanoma, inhibited Bim expression through ERK-dependent phosphorylation and degradation [314, 487]. This might be one mechanism for the anti-apoptotic actions of B-Raf^V600E^ [487].

Petrocca et al. [175] observed that Bim expression is higher in gastric primary tumors than normal tissues. This might be due to upregulation of Bim by oncogenic stress stimuli [480]. Bim_EL_ and Bim_L_ were also found to be expressed at higher levels in prostate cancer cells than normal prostate tissue [30].

#### 4.11.2. Role of Bim in Anoikis

Epithelial cells usually need to attach to extracellular matrix (ECM) or interact with neighboring cells in order to receive integrin signals required for survival. When detached from substratum, the epithelial cells undergo apoptosis through a process termed anoikis. In order to survive, a metastatic cell needs to acquire anchorage independence to overcome anoikis. Bim plays a key role in the anoikis of a variety of tumor cells, such as breast cancer, lung cancer, osteosarcoma, fibrosarcoma, and melanoma [488-490]. Bim is strongly induced after cell detachment as a result of attenuated integrin signal with consequent reduced ERK activity [489]. Overexpression of EGFR results in the maintenance of ERK activity following detachment, thus preventing Bim induction and anoikis [489]. The cell surface glycoprotein mesothelin, which is overexpressed in various tumors, promotes anchorage-independent growth and suppresses Bim expression via ERK activation in human breast cancer cells [490]. Also, the focal adhesion protein PINCH-1, which is a cytoplasmic component of cell-extracellular matrix adhesions, can activate ERK-mediated Bim degradation, besides suppressing Bim transcription [210]. Giannoni et al. [491] observed that integrin engagement leads to the production of reactive oxygen species (ROS) that are responsible for the redox-mediated activation of Src that transphosphorylates EGFR in a ligand-independent manner. EGFR, in turn, activates ERK and Akt signaling pathways that culminate in the degradation of Bim [491]. Overexpression of the receptor tyrosine kinase HER2/neu (ErbB2) in breast cancer, led to increased HIF-1α expression, increased ERK and Akt activities, reduced Bim expression and resistance to anoikis [224]. The transcription factor Pokemon renders liver cells resistant to anoikis through suppression of Bim transcription [492]. G1/S cell cycle arrest induced by overexpression of p16^INK4a^, p21^Cip^ or p27^Kip^ leads to sustained ERK activation, Bim suppression and anoikis resistance [493]. However, overexpression of CXCL12 in colorectal carcinoma led to increased sensitivity to anoikis, enhanced expression of Bim and reduced metastatic potential [494].

During mammary morphogenesis, cells that become detached from the extracellular matrix undergo apoptosis, leading to the formation of the mammary lumen [495]. In contrast to normal mammary epithelial cells that require attachment to ECM for survival, inflammatory breast cancer (IBC) cells evade anoikis. ErbB2/EGFR signaling through the ERK/MAPK pathway protects the cells from anoikis by facilitating the formation of a protein complex containing Bim_EL_, LC8 and Beclin-1 [496]. This complex forms as a result of Bim_EL_ phosphorylation on Ser59, and thus Bim_EL_ cannot localize to the mitochondria and cause anoikis. The extracellular matrix metalloproteinase inducer CD147 (EMMPRIN) confers resistance to anoikis through inhibition of Bim, supposedly through the ERK pathway [497]. Hypoxic conditions, that prevail when the lumen of ducts is filled with breast carcinoma cells, inhibit anoikis through suppressing Bim and Bmf expression in the carcinoma cells [495]. The hypoxia-mediated anoikis protection is associated with increased activation of the EGFR/ERK signaling pathway and requires the hypoxia-activated transcription factor HIF-1α [495]. HIF-1α expression predicts poor therapeutic response and clinical outcome in human breast cancers [498] and contributes to resistance to the tyrosine kinase inhibitor Lapatinib in ErbB2-positive breast cancer cells [499]. Downregulation of Protein tyrosine kinase 6 (PTK6), a non-receptor tyrosine kinase highly expressed in HER2^+^ breast cancer, led to p38 activation, Bim induction and apoptosis of Lapatinib-resistant HER2^+^ breast cancer cells [500].

#### 4.11.3. Bim Expression in Leukemia and Implications for Chemotherapy

The Bcr-Abl fusion protein overexpressed in CML suppresses Bim expression [305]. The Bcl-Abl kinase inhibitor imatinib (Gleevec/STI-571) induces killing of Bcr-Abl^+^ CML through a mechanism that depends on Bim and Bad, but not Bmf or Puma [304, 305, 501]. Cytokines antagonized imatinib- and nilotinib-induced apoptosis that was related to reduced Bim accumulation [501]. Drug resistance due to loss of Bim could be overcome by the BH3 mimetic ABT-737 [304].

Similarly, insulin-like growth factor 1 (IGF-1), which acts as a growth and survival factor in multiple myeloma (MM), downregulates Bim expression in these malignant cells [247]. IGF-1 prevented Bim transcription through Akt-mediated inactivation of FoxO3a, and increased proteasomal degradation of Bim_EL_ by activating MAPK [247]. In addition, IGF-1 reduced histone H3 tail Lys9 (H3K9) acetylation and increased H3K9 dimethylation, both contributing to Bim silencing [247]. The deacetylase inhibitor LBH589 induced Bim expression [247]. High levels of Baculoviral inhibitor of apoptosis repeat-containing 5 (BIRC5/Survivin) expression in multiple myeloma cells correlates with reduced Bim transcription in response to IL-6 deprivation and shorter overall survival of patients [502].

Bim was shown to be epigenetically silenced in NPM/ALK^+^ anaplastic large cell lymphoma (ALCL) [503]. The Bim silencing involved the recruitment of the methyl-CpG-binding protein MeCP2 and the SIN3a/histone deacetylase 1/2 (HDAC1/2) co-repressor complex [503]. The deacetylase inhibitor (HDACi) Trichostatin A restored histone acetylation, with concomitant upregulation of Bim [503]. While NPM/ALK induces *de novo* Bim 5′UTR methylation, its later silencing or inhibition by using crizotinib (PF02341066) did not lead to reacetylation of the Bim locus [503].

Treatment resistance in T cell acute lymphoblastic leukemia (T-ALL) is associated with PTEN deletions which result in the activation of the PI3K-Akt pathway, and Notch1-mediated c-Myc overexpression [504, 505]. Treatment with c-Myc or PI3K/Akt pathway inhibitors induced Bim upregulation and apoptosis, indicating that Bim is repressed downstream of c-Myc and PI3K/Akt in high-risk T-ALL [506]. Restoring Bim function in human T-ALL cells using a stapled peptide mimetic of the Bim BH3 domain had beneficial therapeutic effect [506].

Multiple myeloma cells often express high basal Bim levels that are important for bortezomib-induced apoptosis [507]. Repeated exposure to bortezomib led to marked Bim downregulation, Mcl-1 upregulation and drug resistance [507, 508]. The drug resistance could be overcome by combining HDAC inhibitors that upregulate Bim, with ABT-737 that releases Bim from the anti-apoptotic proteins [507]. Chloroquine, that interrupts the autophagic process, could further enhance the cytotoxic effect [507]. 3-Phosphoinositide protein kinase 1 (PDPK1) that is frequently activated in multiple myeloma cells, suppresses Bim expression and is associated with reduced overall survival [509]. IL-6 and IGF-1 in the microenvironment further promote drug resistance through activation of the PI3K/Akt pathway that leads to Bim downregulation and Mcl-1 upregulation [86]. Similarily, the CD28 receptor on multiple myeloma cells promote survival following interaction with its ligand CD80/CD86 on dendritic cells that is related to PI3K activation and Bim downregulation [510]. Interruption of the CD28-CD80/CD86 interaction sensitized multiple myeloma cells to chemotherapy [510]. Multiple myeloma cells also show enhanced NFκB activity, and the NFκB target gene YY-1 is often hyperexpressed resulting in transcriptional repression of Bim transcription [205].

B cell receptor (BCR)- and microenvironmental-derived signals promote survival of chronic lymphoblastic leukemia (CLL) cells through increased proliferation and decreased apoptosis. Surface IgM stimulation increased phosphorylation of Bim_EL_ and/or Bim_L_ by an MEK1/2-dependent mechanism [511]. Other studies have shown that BCR engagement in CLL leads to Akt activation in addition to ERK1/2 activation, both pathways being pro-survival [512]. CLL cells are also characterized by high expression of Mcl-1 that antagonizes the pro-apoptotic effects of Bim [511, 512], and high Mcl-1 expression is associated with poor clinical outcome [513]. This is in contrast to normal B cells where Bim mediates BCR-induced apoptosis [36], unless the MEK/ERK signaling pathway is activated by mitogenic stimuli [514]. Bim is also highly sequestered to Bcl-2 in CLL cells that make Bim rapidly available upon treatment with the BH3 mimetics ABT-737 [515]. Also, ALL cells with Bcl-2 dependency respond well to ABT-737 [516].

#### 4.11.4. Role of Bim in Glucocorticoid-Induced Apoptosis

Glucocorticoids are critical components of combination chemotherapy regimens in pediatric acute lymphoblastic leukemia (ALL). Bim is crucial for mediating cell death in thymocytes, chronic lymphocytic leukemia, T acute lymphoblastic leukemia and multiple myeloma cells induced by glucocorticoids [14, 21, 49, 63, 158, 517-520], and its expression correlates with therapy response in lymphoid malignancies [518, 521, 522]. Glucocorticoid resistance in ALL xenografts was associated with failure to upregulate Bim expression after dexamethasone [522]. Overexpression of Bcl-2 and other anti-apoptotic proteins antagonizes glucocorticoid-induced apoptosis [14, 64]. Bcl-2-dependent resistance could be overcome by staurosporine through a Nur77-dependent mechanism [64]. Glucocorticoid resistance in xenografts and patient biopsies correlated with decreased histone H3 acetylation [522]. Glucocorticoids also lead to apoptosis of other cell types such as osteoblasts and insulin-producing β-cells through upregulation of Bim [458, 523].

The glucocorticoid-induced Bim expression is indirectly mediated through activation of transcription factors such as FoxO3, c-Jun and Runx1 [159, 524, 525]. Another transcription factor upregulated by glucocorticoids that may promote Bim expression is the bZIP transcriptional repressor gene E4BP4 [526-528]. While FoxO3 is uttermost important for the glucocorticoid-induced upregulation of Bim, FoxO3 may also transactivate glucocorticoid-induced leucine zipper (GILZ) [529], which antagonizes FoxO transcription factors through Chromosome region maintenance 1/Exportin 1 (CRM1)-dependent nuclear exclusion of these proteins [530]. GILZ induction may therefore serve as a negative feedback mechanism. Similarly, the glucocorticoid-response gene PLZF/ZBTB16, act as a repressor on Bim-transcription [531]. Interestingly, Jing et al. [518] observed a differential glucocorticoid response in sensitive and resistant pediatric ALL patient-derived xenografts. They demonstrated a novel glucocorticoid receptor binding site in a Bim intronic region (IGR) that was engaged only in the dexamethasone-sensitive xenografts [518]. The absence of GR binding at the Bim IGR was associated with Bim silencing and dexamethasone resistance [518]. Moreover, they show that the glucocorticoid receptor transactivates KLF13, which repressed Myb expression only in the sensitive cells [518]. Sustained Myb expression in resistant xenografts resulted in maintenance of Bcl-2 expression and inhibition of apoptosis [518]. The presence of the detoxification enzyme Glutathione S-Transferase mu 1 (GSTM1) inhibits dexamethasone-induced apoptosis in lymphoblastic leukemia through suppression of Bim [532].

The upregulation of Bim *per se* is insufficient for initiating apoptosis. Bim needs to be activated. One activation mechanism is through interaction with GSK3, which is essential for glucocorticoid-induced apoptosis [63]. Protein kinases that inactivate GSK3 confer resistance to glucocorticoid-induced apoptosis [63]. Sensitization can be achieved by inhibiting Src, PI3K or Akt [63]. Similarly, the mTOR inhibitor rapamycin may sensitize T-ALL and MM cells to glucocorticoid-induced apoptosis through upregulation of Bim and downregulation of Mcl-1 [519, 533, 534]. Proteasomal inhibition may increase Bim expression [535], and therefore forms the rationale for combining bortezomib with glucocorticoids in the treatment of hematopoietic malignancies [517, 536]. However, Bortezomib does not only stabilize Bim, but also Mcl-1, that outranges the pro-apoptotic effect of Bim, thus leading to therapy resistance [537, 538].

#### 4.11.5. Bim Expression in Carcinoma and Implication for Chemotherapy

Bim expression was found to be lost in a large part of renal cell carcinoma (RCC) [539]. Apoptosis sensitivity correlated with Bim protein levels [539]. Inhibition of histone deacetylation restored Bim expression in RCC cell lines [539]. Bim expression was lower in AJCC II-IV stages of melanoma than in AJCC I-II stages [540]. Loss of PTEN in B-Raf^V600E^-mutated melanoma cells confers resistance to the B-Raf inhibitor PLX4720 through the suppression of Bim expression [541]. PLX4720 stimulated Akt activity in PTEN^−^, but not in PTEN^+^ cells [541]. The treatment resistance of PTEN^−^ cells could be overcome by co-treatment of PLX4720 with a PI3K inhibitor that leads to enhanced Bim expression [541].

G1P3, a survival protein induced by interferons and a contributor to poor outcomes in estrogen receptor (ER)-positive breast cancer patients, attenuated the induction of Bim [542]. Elevated expression of G1P3 was associated with decreased relapse-free and overall survival in ER-positive breast cancer patients [542]. Abnormal overexpression of the chaperone-associated E3-ligase C terminus of Hsc70-interacting protein (CHIP) induced apoptosis resistance in breast cancer cells by activating the Akt pathway with subsequent prevention of FoxO-dependent Bim and PTEN transcription [543]. CHIP activates the Akt pathway through targeting PTEN for proteasomal degradation [543, 544]. Pyruvate kinase M2 (PKM2) overexpressed in hepatocellular carcinoma promotes Bim degradation and is associated with poor outcome [545]. Depletion of PKM2 induced apoptosis that could be prevented by simultaneous silencing of Bim [545].

Bim expression was low or intermediate in 64% of EGFR mutation-positive NSCLC and high in 36% of the patients. Those with higher Bim expression showed longer progression-free survival when treated with the EGFR tyrosine kinase inhibitor erlotinib and longer overall survival [546-548]. EGFR signaling prevents Bim expression through activation of the MAPK-ERK and PI3K-Akt signaling pathways. Imatinib that inhibits c-Kit activity in gastrointestinal stromal tumors, leads to an increase in the dephosphorylated and deubiquitinated form of Bim in addition to increased FoxO3a-induced Bim transcription [549].

Overexpression of the differentiation-related gene-1 (Drg1) promotes metastasis of colorectal cancer and confers resistance to the topoisomerase inhibitor irinotecan (CPT-11) [550]. Drg1 interacts with Bim and negatively regulates its stability by promoting its interaction with the ElonginB-Cullin2-CIS ubiquitin-protein ligase complex resulting in proteasomal degradation [550]. In the absence of Drg1, Bim was stabilized and bound more abundantly to Hsp70, thereby sensitizing the tumor cells to irinotecan [550]. Similarily, the Receptor for activated C kinase 1 (RACK1) has been shown to confer paclitaxel resistance to breast cancer cells by binding to both the dynein light chain 1 and Bim_EL_ upon exposure to paclitaxel [551]. RACK1 then promotes the degradation of Bim_EL_ through the ElonginB/C-Cullin2-CIS complex [551]. RACK1 is frequently overexpressed in solid tumors and the CIS protein level was negatively correlated with Bim_EL_ in cancer specimens [551].

##### 4.11.5.1. Role of Bim in TGFβ-induced apoptosis

**Figure 10:**
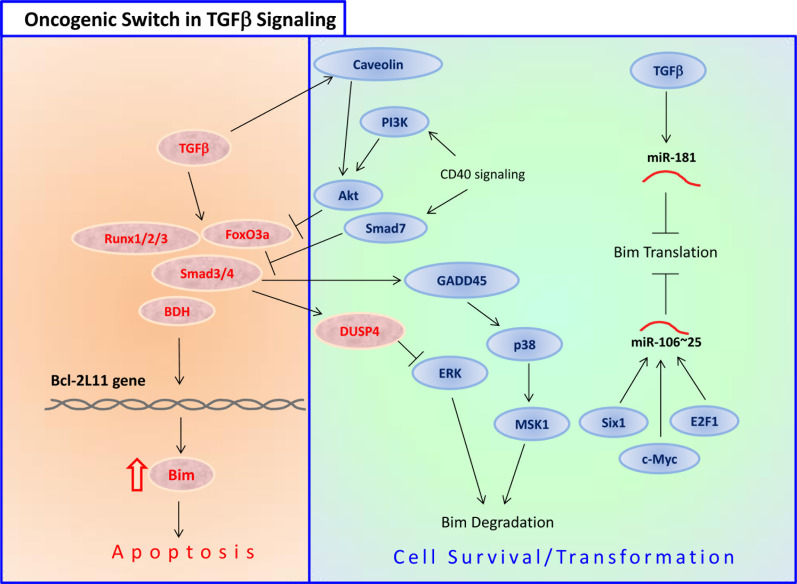
The decision between TGFβ-induced apoptosis and cell transformation TGFβ may induce either apoptosis or cell transformation dependent on the cellular context (Section 4.11.5.1). TGFβ induces Bim expression by activating the Runx1/2/3, Smad3/4 and FoxO3a transcription factors. Also, BDH was found to be important for Bim induction. The simultaneous induction of DUSP4 that antagonizes ERK signaling, leads to stabilization of the Bim protein. TGFβ may also promote cell survival through Akt and MSK1-dependent mechanisms. Upon oncogenic switch, Bim expression is downregulated by reduced transcription, increased degradation and inhibition of Bim translation by the oncomiRs miR-106∼25 and miR-181. miR-106∼25 is upregulated by E2F1, c-Myc and Six1, while TGFβ itself may upregulate miR-181.

TGFβ is a pleiotropic cytokine that can induce various signal transduction pathways, ultimately leading to cell growth, apoptosis or tumor progression, dependent on the cellular context [552] (Figure 10). TGFβ induces apoptosis of normal epithelial cells, osteoclasts and lymphoid cells by a mechanism that depends on Bim and Bmf [42, 152, 153, 166, 553-555]. Acquisition of resistance to TGFβ-induced apoptosis is a critical step for carcinogenesis in many organs.

The transcription factors Smad3/4 and Runx1/3, the p38 MAPK signaling pathway, and the generation of ROS are all involved in the TGFβ-mediated upregulation of Bim transcription [152, 164, 553]. Runx1 cooperates with FoxO3 to transcriptionally induce Bim [165]. TGFβ also induces the expression of the MAPK phosphatase MKP2/DUSP4 to rapidly increase Bim_EL_ by inactivation of ERK1/2 [166]. The Birt-Hogg-Dubé (BHD) tumor suppressor gene was found to be important for TGFβ-induced Bim expression [556]. Loss-of-function mutations in BHD lead to increased risk for skin and kidney cancer [556].

TGFβ could sensitize gastric carcinoma cells to TNFα-induced apoptosis where TGFβ is responsible for Smad3-induced Bim transcription and TNFα to JNK-mediated stabilization of the Bim protein [153]. The tyrosine kinase inhibitor sorafenib could sensitize hepatocellular carcinoma cells to TGFβ through Bim upregulation and Mcl-1 downregulation [557]. TGFβ could also sensitize non-small cell lung carcinomas to the broad-acting tyrosine kinase inhibitor dasatinib through upregulation of Bim and downregulation of Smad7 [558]. Smad7 is a negative regulator of the TGFβ signaling pathway that inhibits Bim expression [42]. miR-106b∼25 that targets Bim, also targets Smad7, resulting in activation of TGFβ signaling and induction of EMT in human breast cancer [559]. Thus, miR-106b∼25 promotes tumor progression by a dual mechanism. The homeotic transcription factor Six1, which is involved in breast cancer progression and metastasis through upregulation of miR-106b∼25, switches the TGFβ signaling from being tumor suppressive to tumor promotional [559]. The miR-106b∼25 cluster is also upregulated by E2F1 that leads to TGFβ-resistance in gastric cancer [175].

Early stage intestinal adenomas with mutations in the adenomatous polyposis coli (APC) gene were more sensitive to TGFβ-induced apoptosis than wild-type intestinal crypts [554]. This was explained by higher Bim levels in the APC mutant cells, and IGF-R signaling in wild-type cells that antagonizes Bim [554]. Mutant K-Ras oncogene activation led to TGFβ resistance in APC-mutant cells due to activation of ERK1/2 [554]. The K-Ras mutant cells were still sensitive to BH3 mimetics, suggesting that the resistance is due to impaired Bim upregulation and not defects in downstream pathways [554].

In cultured hepatocytes, TGFβ rapidly activates Akt through a mechanism dependent on Caveolin-1, leading to suppression of Bim expression and apoptosis [560]. In highly metastatic breast cancer cells, autocrine TGFβ protects the cells from apoptosis through suppression of Bim [561]. In these cells, TGFβ suppressed the forkhead box C1 (FoxC1) required for Bim expression [561]. TGFβ may also promote breast cancer metastasis through upregulation of miR-181a that targets Bim [262]. Moreover, TGFβ activates mitogen- and stress-activated protein kinase 1 (MSK1) that prevents TGFβ-induced apoptosis through downregulation of Bim [562]. Induction of MSK1 activity by TGFβ depends on Smad4-induced GADD45 expression, which in turn activates p38 MAPK that positively regulates MSK1 [562]. These activities of TGFβ may explain its dual actions on tumorigensis.

## 5. THERAPEUTIC APPLICATIONS

Under normal conditions, Bim expression is tightly and timely regulated. However, excessive Bim activation may lead to neurodegenerative diseases, liver damage and diabetes, while reduced Bim expression is associated with autoimmune diseases, increased risk for cancer development and poor response of malignant cells to chemotherapeutics (Figure 8). One strategy to treat these conditions is through regulating Bim expression and/or activity.

In Parkinson's disease for instance, excessive ROS production due to mitochondrial Complex I dysfunction is responsible for JNK-mediated activation of Bim that leads to degeneration of dopaminergic neurons of the substantia nigra pars compacta (SNpc) [445]. Inhibition of JNK or neutralizing ROS by using anti-oxidants should help to attenuate disease progression. This may also be applicable for diabetes that is also caused by ROS-induced JNK-dependent Bim activation [188]. Osteocalcin reduces high glucose-induced H_2_O_2_ levels and protects β-cells from apoptosis [563].

As Mst1 leads to Pdx-1 degradation and Bim upregulation [459], inhibition of Mst1 may also prevent β-cell dysfunction. Mst1 is activated by ROS and promotes apoptosis through phosphorylation of and activation of FoxO transcription factors [564]. Mst1 activity is inhibited by interaction with the redox sensor thioredoxin-1 (Trx1) [564], whose activity, in turn, is antagonized by Thioredoxin-interacting protein (Txnip). Txnip is upregulated by high glucose and dexamethasone in insulin-producing β-cells, leading to apoptosis of these cells [565, 566]. Incretins such as GLP-1 and exendin-4, protect against β-cell damage [567]. GLP-1 has been shown also to diminish neuronal degeneration and death caused by NGF deprivation through suppressing Bim induction [369]. Thus, GLP-1 and exendin-4 are not only beneficial for the treatment of type 2 diabetes, but also for neurodegenerative disorders such as Alzheimer's and Parkinson's diseases [568, 569]. Incretins could also delay the onset of diabetes and neurodegeneration that appear in Friedriech's ataxia disorder caused by Frataxin deficiency [570].

Untimely activation of Bim in activated specified T cells may lead to premature resolution of the immune response, resulting in persistent infection, chronic inflammation and cancer development. Increasing the survival of viral-specific or tumor-specific T cells, e.g., by preventing PD-1 signaling or increasing 4-1BB (CD137) signaling [410], should lead to increased anti-viral and anti-tumor immune responses. On the other side of the Bim coin, too little Bim expression in hematopoietic cells may lead to skewed immune responses.

Chemoresistance in cancer cells is often due to improper Bim function that might be due to disturbance of one or more of the regulatory pathways described in this review. These include reduced transcription due to excessive PI3K-Akt activation, hyperexpression of transcriptional repressors (e.g., YY1) and/or epigenetic silencing; altered Bim splicing; reduced translation due to elevated expression of microRNAs or RNA-binding proteins; reduced protein stability due to hyperactivation of the Ras-MEK-ERK pathway; and Bim sequestration to anti-apoptotic proteins among them overexpression of Mcl-1 is a critical determinant. Different chemotherapeutic interventions have been developed to restore Bim activity that act either upstream or downstream to the critical regulatory point. These include suppression of the inhibitory signal transduction pathway, prevention of Mcl-1 expression and use of BH3 mimetics. These approaches will be described in more details below.

### 5.1. Use of BH3 Mimetics as Anti-Cancer Agents

Compounds that mimic the BH3 domain of Bim may disrupt the interaction of BH3-containing proteins to anti-apoptotic Bcl-2 proteins, thus releasing BH3-containing proteins to initiate intrinsic apoptosis. These drugs will only be efficient against tumors that show dependency on anti-apoptotic proteins. ABT-737 is a BH3 mimetic that binds with high affinity to and antagonizes the functions of Bcl-2 and Bcl-xL, but not Mcl-1 [571]. ABT-737 displaces Bim from the BH3-binding pocket of Bcl-2, allowing Bim to activate Bax and induce mitochondrial outer membrane permeabilization (MOMP) [515]. The amount of Bim sequestrated to Bcl-2, rather than total Bcl-2 expression levels, determines cellular sensitivity to ABT-737 [309, 515, 572]. An increase in Bim expression, e.g., by using the HDAC inhibitor suberoyl bis-hydroxamic acid (SBHA), potentiates the effect of ABT-737 [573].

Preclinical studies demonstrated that ABT-737 induces apoptosis and potentiates the anti-tumor activity of multiple agents in various cancers, including leukemia [571]. The BH3 mimetic ABT-737 increased the response to EGFR tyrosine kinase inhibitors [31]. But since ABT-737 antagonizes Bcl-2 and Bcl-xL, but not Mcl-1, which is overexpressed, among others in NSCLC [574], the effect of this BH3 mimetic is limited, unless combined with a drug reducing Mcl-1 expression. The dual PI3K/mTOR inhibitor NVP-BEZ235 that decreases Mcl-1 expression, synergized with ABT-737 in inducing apoptosis of ovarian carcinoma cells, provided that Bim expression was induced [575]. Simultaneous inhibition of the ERK1/2 pathway restored Bim expression and sensitized low Bim-expressing cancer cells to NVP-BEZ235/ABT-737 treatment [575]. Also Rahmani et al. [576] observed that concomitant inhibition of the PI3K/Akt/mTOR pathway and Bcl-2 using BEZ235 and ABT-737 increased apoptosis in human myeloid leukemia cells. This treatment led to reduced Mcl-1 expression, GSK3 activation, release of Bim from Bcl-2/Bcl-xL and release of Bak and Bax from Mcl-1/Bcl-2/Bcl-xL [576]. Treatment of high-grade serous ovarian cancer *ex vivo* with ABT-737 and carboplatin, which can indirectly inhibit Mcl-1, showed a requirement for Bim and low activity of ERK to obtain a response to ABT-737 [577]. In the Eμ-Myc mouse model, caloric restriction reduced Mcl-1 expression through inhibition of protein translation and sensitized Eμ-Myc lymphoma cells to ABT-737 treatment [578].

Although Mcl-1 dominance renders squamous cell carcinoma cells resistant to ABT-737, the HDAC inhibitor vorinostat primes them for sensitivity to ABT-737 by shuttling Bim from Mcl-1 to Bcl-2/Bcl-xL, resulting in synergy for this drug combination and sustained tumor regression *in vivo* [579]. Somatic FBW7 mutation in squamous cell carcinoma cells is associated with stabilized Mcl-1 and high Bim levels, resulting in a poor response to standard chemotherapy, but a robust response to HDAC inhibitors and enhanced synergy with the combination vorinostat/ABT-737 [579]. Also, combined treatment of ABT-737 with a HDAC inhibitor could overcome adaptive bortezomib resistance of multiple myeloma cells through re-expression of Bim [507]. Chloroquine that disrupts autophagy, further enhanced HDACi/ABT-737 lethality in these cells [507].

ABT-263 (Navitoclax) is a clinical derivative of ABT-737 that are currently undergoing phase I and II clinical evaluation in various tumor types including leukemia [580-582]. *In vivo*, Navitoclax induced durable and complete tumor regression in a murine xenograft model of acute lymphocytic leukemia and significantly improved the cure rate of rituximab (anti-CD20 mAb) plus chemotherapy in a xenograft model of mantle cell lymphoma [583]. Small-cell lung cancer (SCLC) with high Bim expression is among the most sensitive cells to ABT-263 treatment [584]. As with ABT-737, ABT-263 activity is directed against Bcl-2 and Bcl-xL, but not Mcl-1, making Mcl-1 expressing cells treatment resistant. Simultaneous treatment with a TORC1/2 inhibitor (e.g., AZD8055) that reduces Mcl-1 expression improved the response of SCLC to ABT-263 [584].

Alford et al. [585] also showed that primary B-ALL cells expressing high levels of Bcl-2 exhibited great sensitivity to ABT-263 and ABT-199. ABT-263 disrupted Bcl-2:Bim interaction in the cells [585]. However, overexpression of Mcl-1 rendered the B-ALL cells resistant to ABT-263 and ABT-199 [585]. The Mcl-1 and Bcl-xL-dependent resistance to ABT-199 could be overcome by inhibiting the PI3K/Akt/mTOR pathway in lymphoid malignancies [586]. NVP-BEZ235, a dual inhibitor of AKT and mTOR, reduced Mcl-1 levels causing Bim release from Mcl-1 and Bcl-xL, thus leading to cell death by Bax activation [586]. The PI3Kδ inhibitor GS-1101 (idelalisib) downregulated Mcl-1 and sensitized resistant cells to ABT-199 [586].

Obatoclax (GX15-070) binds to Mcl-1 and A1, besides Bcl-2, Bcl-xL and Bcl-w, albeit at low affinity [587]. Obatoclax also kills Bak/Bax deficient cells, thus having additional effects in addition to be a BH3 mimetic [588]. It leads to the upregulation of Noxa that promotes the dissociation of Bak from Mcl-1 [589]. Obatoclax also induces autophagy and necroptosis [590]. Obatoclax is not efficient as a single agent to treat cancer and may cause neurological toxicity that limits its use. Obatoclax may increase the susceptibility of multiple myeloma cells to other chemotherapeutic drugs such as melphalan, dexamethasone and bortezomib [591]. Other Mcl-1 inhibiting agents such as gossypol and its synthetic analogue AT-101 could sensitize otherwise glucocorticoid-resistant MLL-arranged ALL cells to glucocorticoid-induced apoptosis [592]. These authors showed that gossypol and AT-101 increased Bim expression and the apoptosis could proceed without downregulation of Mcl-1 [592].

A series of indole-2-carboxylic acid derivatives have been formulated to target Mcl-1 [593]. One of these compounds, A-1210477, induces apoptosis and kills multiple myeloma and non-small cell lung cancer cell lines [593]. A-1210477 disrupts the Mcl-1:Bim and Mcl-1:Noxa complexes, while having no effect on Bcl-2:Bim interactions [593]. A-1210477 synergized with the Bcl-2/Bcl-xL inhibitor Navitoclax to kill a variety of cancer cell lines [593]. The pan-Bcl-2 inhibitor (−)B197D6 induced apoptosis of acute myeloid leukemia (AML) cells by disrupting Mcl-1/Bim and Bcl-2/Bax interactions [594].

Kazi et al. [595] developed a BH3 α-helical mimetic BH3-M6 that binds to Bcl-xL, Bcl-2 and Mcl-1, thereby preventing their binding to Bax, Bak, Bad and Bim. BH3-M6 disruption of these protein-protein interactions is associated with cytochrome C release from mitochondria, caspase 3 activation and Poly(ADP-ribose)polymerase (PARP) cleavage [595]. BH3-M6 sensitized tumor cells to a proteasome inhibitor [595]. LaBelle et al. [596] developed a hydrocarbon-stapled peptide of the Bim BH3 helix that targeted Bcl-2 family members with high affinity, leading to cell death of otherwise resistant hematologic malignancies. The stapled peptide is structurally stable, protease-resistant and cell-permeable. The stapled Bim BH3 helix (amino acids 146-166) contains an i, i+4 all-hydrocarbon crosslink spanning positions 154 and 158 [596].

### 5.2. Use of Protein Kinase Inhibitors to Increase Bim Expression and Activity

The use of BH3 mimetics is hampered by high hepatotoxicity, which limits their applications. Also, the dependency on sufficient Bim expression for apoptosis induction makes BH3 mimetics unsuitable for overcoming tumor resistance caused by low or absent Bim expression [577]. In many cases, Bim expression is repressed through activation of the PI3K-Akt-mTOR and/or Ras-MEK-ERK1/2 signaling pathways, and inhibition of these pathways might be sufficient for Bim upregulation and induction of apoptosis. Usually combined treatment of drugs targeting different pathways is advantageous.

The mTOR inhibitors rapamycin (Sirolimus) and RAD001 (Everolimus) have long been known to increase the sensitivity of malignant hematopoietic cells to chemotherapeutic drugs such as glucocorticoids [519, 533, 534, 597-599], rituximab [600] and IFNα [601], and solid tumors to cisplatin [602], mitoxantrone [603], carboplatin [604], vinorelbine [604] and taxoids [603, 604]. One of the mechanisms by which rapamycin sensitizes tumor cells is through downregulation of Mcl-1 with simultaneous upregulation of Bim [533, 534]. Inhibition of glucose metabolism or mTORC1 leads to decreased Mcl-1 expression and upregulation of Bim, thereby sensitizing diffuse large B cell leukemic cells to ABT-737 [605]. As rapamycin inhibits TORC1, but not TORC2, what leads to a feedback loop activating the Akt pathway [606] and further Mcl-1 stabilization [108], dual TORC1/TORC2 inhibitors have been developed with enhanced synergistic effect on other chemotherapeutics. In the case of rapamycin sensitization of oral squamous cell carcinoma cells to cisplatin, rapamycin increased FoxO3a protein stability and cisplatin inhibited the feedback activation of Akt by rapamycin [602]. This resulted in FoxO3a activation and Bim induction [602]. Similarily, the Akt inhibitor MK-2206 enhanced the anti-tumor effect of rapamycin on neuroblastoma cells [607]. The dual TORC1/2 inhibitor AZD8055 sensitized colorectal cancers with K-Ras or B-Raf mutations to ABT-263 by suppressing Mcl-1 [608]. AZD8055 induced apoptosis in laryngeal carcinoma by upregulating Bid, Bad and Bim [609]. The dual mTORC1/TORC2 inhibitor OSI-027 induced apoptosis in specimens from B cell acute lymphoblastic leukemia, mantle cell lymphoma and marginal zone lymphoma by a mechanism that was dependent on Puma and Bim [610]. The PI3K/mTOR dual inhibitor NVP-BEZ235 reduced Mcl-1 expression and sensitized ovarian carcinoma cells to ABT-737 through a Bim-dependent mechanism [575]. Inhibition of the PI3K/Akt pathway also sensitized lymphoid malignancies to glucocorticoid-induced apoptosis, which can be explained by the requirement for both GSK3 and Bim [63].

Sorafenib, a potent multikinase inhibitor, induces apoptosis of human acute myeloid leukemia (AML) cells through downregulating Mcl-1 and enhancing binding of Bim to Bcl-2 and Bcl-xL [611]. Sorafenib may also upregulate Bim expression [612]. Combined treatment of sorafenib with obatoclax or ABT-737 had a synergistic effect in reducing tumor growth [611, 612]. Similarly, combining obatoclax with the pan-CDK inhibitor flavopiridol increased apoptosis of both drug-naïve and drug-resistant multiple myeloma cells in a Bim- and Noxa-dependent mechanism [613]. Flavopiridol inhibited Mcl-1 transcription, but increased transcription of Bim and its binding to Bcl-2 and Bcl-xL [613]. Obatoclax prevented Mcl-1 recovery and caused release of Bim from Bcl-2, Bcl-xL and Mcl-1, accompanied by activation of Bak and Bax [613].

Simultaneous targeting of PI3K and mTOR using NVP-BGT226 induced apoptosis in multiple myeloma cells by upregulating Bim [614]. The growth stimulatory effect of IGF1 and IL-6 on multiple myeloma cells was completely abrogated by NPV-BGT226 [614]. NVP-BGT226 has also been shown to exert cytotoxic effects against other cancer cell types such as ALK-positive anaplastic large cell lymphoma [615] and hepatocellular carcinoma [616] and has entered Phase I/II clinical trials for breast cancer [617].

Inhibition of EGFR using the tyrosine kinase inhibitors (TKIs) erlotinib or gefitinib could induce apoptosis of NSCLC with mutant constitutively active EGFR (ΔL747-S752 or L858R) through a process dependent on Bim [306, 307, 309]. The three Bim isoforms Bim_EL_, Bim_L_ and Bim_S_ were induced by erlotinib in single mutated EGFR-sensitive cells, but not in PTEN-deficient or double mutated EGFR (additional T790M mutation)-insensitive cells [307, 308, 546]. Pretreatment mRNA levels of Bim predicted the capacity of EGFR inhibitors to induce apoptosis in EGFR-mutant cancer cells [618]. TKI-resistance usually develops upon repeated treatment which was shown in some cases to be due to overexpression of paxillin [619] or neutrophil gelatinase-associated lipocalin (NGAL) [620]. Both paxillin and NGAL activate ERK, resulting in enhanced Bim degradation and increased Mcl-1 expression [619, 620]. Patients with lower plasma NGAL levels showed a better erlotinib response [620]. TKI-mediated reactivation of ERK1/2 is also due to reduced Akt-dependent Ets-1-mediated DUSP6 induction [621]. TKI resistance could be overcome by simultaneous delivery of an ERK inhibitor (AZD6244) [619]. Also, the HDAC inhibitor vorinostat could circumvent EGFR-TKI resistance in EGFR-mutant NSCLC cells [32]. Another mechanism for acquired resistance to erlotinib or gefitinib is the appearance of a secondary EGFR mutation. A second-generation TKI afatinib binds to the mutated EGFR, but causes redistribution of EGFR to the cell surface through a Rab11a-dependent recycling that reduces its effectiveness [622]. However, combining afatinib with the anti-EGFR monoclonal antibody cetuximab synergistically induced apoptosis of the erythroleukemic K562 cells through upregulation of Bim [622].

Hepatocyte growth factor (HGF) confers EGFR TKI resistance by inducing two cancer-promoting functions. HGF makes the cancer cells independent of EGFR signaling and enables EGFR to interact with other proteins such as CUB domain-containing protein-1 (CDCP1), EphA2 and AXL, forming a c-Met (HGFR)-EGFR cross-talk that can't be inhibited by EGFR TKI treatment [546]. The dual anaplastic lymphoma kinase (ALK) and c-Met receptor tyrosine kinase inhibitor CM-118 inhibited ALK-signaling and HGF-induced c-Met signaling, thus interrupting the c-Met-EGFR cross-talk. This compound led to inhibition of proliferation or induction of apoptosis in c-Met- and ALK-addicted cancer cells [623]. Combined with EGFR inhibitors, CM-118 induced apoptosis of c-Met amplified NSCLC cells through Bim upregulation and Mcl-1 downregulation [623]. mTOR inhibitors further potentiated the anti-tumor effect of CM-118 [623]. Also, the dual ALK/c-Met inhibitor crizotinib that is effective in treating EML4-ALK positive and c-Met amplificated NSCLC patients [624], induces apoptosis by a Bim-dependent mechanism [625]. Bim upregulation by ALK inhibition was a result of ERK suppression [626]. Crizotinib also induced apoptosis of c-Met-positive gastric cancer cells through upregulation of Bim [627]. Foretinib, which is an oral multikinase inhibitor that inhibits c-Met, Recepteur d'origine nantais (RON; MST1R), the Gas6 tyrosine kinase receptor AXL and vascular endothelial growth factor receptor (VEGFR), improves overall survival in a preclinical model of hepatocellular carcinoma, through upregulation of Bim and p27 and downregulation of cyclin B1 and phosphorylated c-Myc [628]. Overexpression of AXL in CML led to imatinib resistance that was related to increased PKCα/β and ERK1/2 activation [629]. The AXL receptor tyrosine kinase receptor is also involved in the survival of B-CLL cells. Combining the AXL inhibitor TP-0903 with Bruton's tyrosine kinase inhibitors (e.g., ibrutinib) reduced the expression of Mcl-1, Bcl-2 and XIAP, while upregulated Bim expression, resulting in increased B-CLL apoptosis [630].

Treatment of B-Raf^V600E^/PTEN-null melanoma cells with the B-Raf inhibitor vemurafenib led to increased fibronectin expression that abrogates the therapeutic response due to enhanced PI3K-Akt signaling and Mcl-1 expression [631]. Simultaneous use of a PI3K inhibitor overcomes the drug resistance [631]. Acquired B-Raf inhibitor resistance in melanoma cells could also be overcome by simultaneous use of the ATP-competitive MEK/Aurora kinase inhibitor BI-847325 [632]. BI-847325 decreased the expression of MEK and Mcl-1, while increased the expression of Bim [632]. Combining vemurafenib with the MEK inhibitor trametinib increased Bim upregulation and apoptosis [633]. Trametinib alone caused activation of Akt in B-Raf non-V600 mutated cells that was nullified with the combination of vemurafenib [633]. Another approach to overcome vemurafenib resistance is to use the Hsp90 inhibitor XL888 [634] or ganetespib [635]. XL888 induced apoptosis of vemurafenib-resistant melanoma cells by increasing FoxO3a-induced Bim transcription and downregulation of Mcl-1 [634].

Altogether, these studies repeatedly show that Bim function can be revived in cancer cells, and the most efficient treatment is usually a drug combination that simultaneously targets various critical nodal points ultimately leading to Bim upregulation (e.g., PI3K-Akt inhibitors, HDAC inhibitors), Bim stabilization (e.g., ERK inhibitors, proteasomal inhibition), and Bim release from sequestered intracellular storage (e.g., BH3 mimetics, JNK activation, dual TORC1/2 inhibitors).

### 5.3. The Bim Status as a Prognostic Criterium

Usually cancer cells with high basal Bim expression show better response to Bim-dependent chemotherapy than those with low Bim expression, which can be explained by the rapid available pre-made Bim, a state termed “primed for apoptosis”. When Bim is expressed at relative high basal levels, the cancer cells have often developed a mechanism (e.g., concomitant Mcl-1 or Bcl-2 upregulation) that antagonizes the pro-apoptotic function of Bim. As the cancer cells become dependent on the anti-Bim mechanism for survival, targeting this mechanism will induce Bim-dependent cancer cell death. For instance, it was sufficient to reduce Mcl-1 levels to induce Bim-dependent apoptosis in c-Myc positive HER-positive breast cancer cells [192]. Not only the basal Bim level account for better prognosis, but also the ability of the cancer cells to elevate Bim expression in response to chemotherapy is important for the clinical response. This has been demonstrated for glucocorticoid susceptibility of pediatric acute lymphoblastic leukemia (ALL) [521, 522, 636]. Bim was only upregulated in prednisolone/dexamethasone-sensitive pediatric ALL cells, but not in those being resistant [521, 636]. Patients whose Bim protein expression levels failed to upregulate at day 8 compared to day 0 had a poorer event-free survival than those patients whose Bim expression levels did upregulate [521]. The resistance was correlated with reduced histone H3 acetylation and could be overcome by the HDAC inhibitor vorinostat [522]. Also, Bim induction in chronic lymphocytic leukemia in response to glucocorticoids correlated with the response rate [637].

The pretreatment RNA levels of Bim predicted the ability of EGFR, HER2 and PI3K inhibitors to induce apoptosis in EGFR-mutant, HER2-amplified and PIK3CA-mutant cancer cells, respectively, while Bim levels did not predict responsiveness to standard chemotherapies such as gemcitabine and cisplatin [618]. Also, the extent of Bim induction in response to the inhibitors is predicative [618]. Patients with EGFR-mutant lung cancers expressing high levels of Bim showed longer progression-free survival (PFS) than those with tumors expressing low levels of Bim [618]. PFS to erlotinib was longer for high Bim-expressing NSCLC than those with intermediate or low Bim expression [548]. Bim RNA levels may be assessed in diagnostic cancer specimens to predict which patients will benefit from single-agent kinase inhibitor [618]. Glioblastoma multiforme patients with high levels of phospho-Bad (Ser136) and phospho-Bim (Ser69) indicative for increased ERK activation displayed shorter overall survival [638]. IgM-induced phosphorylation of Bim correlated with progressive disease in CLL patients with mutated IGHV genes [511].

Bim deletion polymorphism was significantly associated with the clinical efficacy of tyrosine kinase inhibitors in terms of response rate and disease control rate in EGFR-mutated NSCLC patients, but not in CML or hepatocellular carcinoma (HCC) [639]. EGFR-mutated NSCLC patients harboring Bim deletion polymorphism was associated with a shorter PFS, while there was no association with overall survival [639]. Shorter overall survival of pediatric acute lymphoblastic leukemia patients was associated with the Bim C29201T polymorphism located in the BH3 domain [34].

A strategy termed BH3 profiling using BH3 peptides can identify apoptotic defects in cancer cells. BH3 profiling of CLL can identify cells that require Bcl-2 for survival and can predict sensitivity to ABT-737 [515, 572]. Bcl-2 dependence correlates with high levels of Bim sequestered by Bcl-2 [572].

## 6. CONCLUSIONS

In this review we have attempted to survey the various signal transduction pathways regulating the expression and activity of Bim. The multiple regulatory mechanisms affecting Bim, make it critical for determining the cell fate in response to any changes in the cell's microenvironment or in the intrinsic cell signal pathways. Many of these pathways can also affect Mcl-1 expression, often in an inverse manner than Bim, thereby tipping the Bim/Mcl-1 balance towards either apoptosis or survival. As an essential component of the intrinsic apoptotic pathway, alterations in Bim expression affect almost every process in the body from regulating the immune system, the nerve system, β-cell and liver physiology to affecting mammary lumen formation and cancer progression. Therefore there is no wonder why abnormal Bim expression causes a range of pathological conditions (Figure 8). As Bim expression is regulated differentially in different cell types, each pathological condition needs to be considered separately. For instance, an increase in intracellular cAMP levels makes malignant lymphoid cells more susceptible to an apoptotic stimulus, while in insulin-producing β-cells and neurons it is protective. Another example of a cell-specific effect is JNK activation. In neuronal cells and β-cells excessive JNK activation is detrimental, activating Bim-dependent cell death, while in lymphoid cells, JNK activation actually antagonizes apoptosis. This cell-specific effect may be explained by Bim sequestration to microtubules in the former cell types where JNK phosphorylates and releases Bim, while in lymphoid cells pre-made Bim is tonically sequestered to anti-apoptotic proteins rather than microtubules. Death of various immune cells can be prevented by respective cytokines. For instance, IL-7 can rescue T cell death, whereas IL-4 and BAFF prevent B cell death. In the context of cancer, increased survival of tumor-specific cytotoxic T cells can be achieved by interrupting the PD-1L/PD-1 or enhancing the 4-1BB signaling using specific antibodies, thereby increasing the anti-tumor effect [410]. On the contrary, in autoimmune diseases, elimination of autoreactive T and B cells is desirable. Several approaches have been examined that can increase apoptosis of autoreactive immune cells. These include the use of ABT-737 mimetics [640], monoclonal antibody against IL-6R (e.g., tocilizumab or siltiximab) [641, 642], mTOR inhibition [643] or activation of the PD-1 pathway [644], all tipping the Bim/Mcl-1 balance in favor of Bim. Another approach is to prevent the death of target cells affected by the autoimmune cells. This is best exemplified by the intrinsic higher β-cell death in diabetes-predisposed patients that triggers insulitis which, in turn, promotes further β-cell death, thereby forming a bad negative feedback loop. As we have learned, some of the diabetes susceptibility genes e.g., GLIS3, PTPN2, BACH2 and Cathepsin H, regulate Bim expression. Thus, preventing Bim upregulation in β-cells should reduce the propensity to develop diabetes. Similarly, preventing β-amyloid aggregation should restrain the development of Alzheimer's disease.

On the other side of the pathological spectrum lies the cancer issue. Each tumor cell has often developed its own characteristic dependency on specific protein kinases that antagonizes Bim expression, while fortifying Mcl-1 expression. Enormous efforts have been made to develop drugs that specifically target the specific upstream kinase responsible for tumor cell survival. Classical examples are the Bcr-Abl/c-Kit inhibitor imatinib, the EGFR inhibitors erlotinib and gefitinib, the Flt3 inhibitor ponatinib and the B-Raf^V600E^ inhibitor vemurafenib, all inducing Bim expression. Although initial clinical response is often seen when using these drugs, adaptive resistance mechanisms frequently develop, leading to cancer recurrence. The main resistance mechanisms involve the single or combined reactivation of the PI3K-Akt-mTOR and Ras-Raf-MEK-ERK1/2 survival pathways that prevent Bim expression. The current trend is to combine the specific targeting therapies with inhibitors targeting the downstream survival protein kinases. This combined treatment ensures proper reactivation of Bim. Other approaches include the combined treatment of a tyrosine kinase inhibitor with an HDAC inhibitor that aims to synergistically increase Bim expression. In summary, drug-induced apoptosis of tumor cells that depends on the intrinsic apoptotic pathway, needs a sufficient amount of Bim. Any mechanism that prevents Bim expression in cancer cells should therefore be targeted.

## HIGHLIGHTS

- Bim is essential for initiating the intrinsic apoptotic pathway and is often required for efficient response to chemotherapeutics.
- Bim expression and activity are tightly regulated at the transcriptional, translational and post-translational levels, making it readily available when required.
- Protein kinases and phosphatases regulate Bim stability and activity, while microtubules and anti-apoptotic proteins of the Bcl-2 family sequester Bim, keeping it in an inactive state.
- Elevated Bim expression leads to neurodegenerative disorders, liver damage and diabetes, while suppression of Bim supports tumor progression and metastasis.
- Therapeutic interventions aim to prevent neuronal, hepatocyte and β-cell apoptosis by reducing Bim expression, while, on the contrary, should increase Bim expression in cancer cells for apoptosis induction.
- Chronic inflammation prevailing under persistent viral infections and in the tumor microenvironment can be overcome by rescuing cytotoxic T cells from Bim-dependent cell death.

## SUPPLEMENTARY MATERIAL TABLE



## Abbreviations

ALK: Anaplastic lymphoma kinase
ALL: Acute lymphoblastic leukemia
AML: Acute myeloblastic leukemia
AP-1: Activator protein-1
APC: Anaphase promoting complex
APRIL: A proliferation-inducing ligand
ARE: AU-rich element
ASK1: Apoptosis signal-regulating kinase 1
ATF4: Activating transcription factor 4
ATM: Ataxia telangiectasia-mutated protein kinase
Bak: Pro-apoptotic Bcl-2 antagonist/killer
BACH2: Basic leucine zipper transcription factor 2
BAFF: B cell activating factor belonging to the tumor necrosis factor family
Bax: Bcl-2-associated X
Bcl-2: B cell lymphoma-2
Bcl-xL: B cell lymphoma-extra large
BCR: B cell receptor
Bcr-Abl: Breakpoint cluster region-Abelson murine leukaemia viral oncogene
BDH: Birt-Hogg-Dubé
Bfl-1: Bcl-2-related protein A1
BH: Bcl-2 homology domain
BHRF-1: BamHI fragment H rightward open reading frame 1 (Epstein-Barr virus homologue of Bcl-2).
Bid: BH3 interacting domain death protein
Bim: Bcl-2 interacting mediator of cell death/BOD (Bcl-2 related ovarian death agonist)
BMCC1: BNIP2 and Cdc42GAP homology (BCH) motif-containing molecule at the carboxyl-terminal region 1
BNIP2: Bcl-2/adenovirus E1B 19kDa interacting protein 2
BOD: Bcl-2 related ovarian death agonist
BRCA-1: Breast cancer susceptibility protein 1
Brd4: Bromodomain 4
CBP: CREB-binding protein
Cdk4: Cyclin dependent kinase 4
CHIP: C terminus of Hsc70-interacting protein
CHOP: C/ERB homologous protein
CK2: Casein kinase II
CLL: Chronic lymphocytic leukemia
CML: Chronic myeloid leukemia
CREB: cAMP response element-binding protein
DBD: Dynein binding domain
E2F: E2 promoter binding factor
EBNA3A/3C: Epstein-Barr virus determined nuclear antigen 3A or 3C
EBV: Epstein-Barr virus
ECM: Extracellular matrix
EGFR: Epidermal growth factor receptor
Egr-1: Early growth response protein 1
EMT: Epithelial to mesenchymal transition
ER: Endoplasmic reticulum
ERK1/2: Extracellular signal-regulated kinases 1 and 2
EZH2: Enhancer of zeste homolog 2
Fbw7: F-box and WD repeat domain-containing 7
FGF4: Fibroblast growth factor-4
Flt3: FMS-like tyrosine kinase 3
FoxO3a: Forkhead box O3 isoform a
GADD45a: Growth arrest and DNA-damage-inducible protein 45 alpha
GATA-1: Transcription factor binding to the DNA sequence GATA
GILZ: Glucocorticoid-induced leucine zipper
GLIS: Gli similar
GLP-1: Glucagon-like peptide-1
GSK3: Glycogen synthase kinase 3
H2AX: Histone 2A variant X
HCC: Hepatocellular carcinoma
HBV: Hepatitis B virus
HCV: Hepatitis C virus
HDAC: Histone deacetylase
HER2: Human epidermal growth factor receptor 2
HIF-1: Hypoxia-inducible factor 1
hnRNP C: Heterogeneous nuclear ribonuclear protein C
HoxB8: Homeobox protein B8
HGF: Hepatocyte growth factor
Hrk/DP5: Harakiri protein
Hsc70: Heat-shock cognate protein 70
Hsp27: Heat shock protein 27
HSV: Herpes simplex virus
HtrA2: High temperature requirement serine protease A2
IBC: Inflammatory breast cancer
IL-3: Interleukin-3
IL-7: Interleukin-7
IP3R: Inositol triphosphate receptor
JAK: Janus-family tyrosine kinase
JNK: c-Jun N-terminal kinase
Keap-1: Kelch-like ECH-associated protein-1
KLRG1: Killer-cell lectin like receptor G1
LCMV: Lymphocytic choriomeningitis virus
LPS: Lipopolysaccharide
MAPK-1: Mitogen-activated protein kinase 1
Mcl-1: Myeloid cell leukemia-1
MCMV: Mouse cytomegalovirus
MEK: Mitogen-activated protein kinase (MAP)-extracellular signal-regulated protein kinase (ERK) kinase
MKP2/DUSP4: MAP kinase phosphatase 2/Dual specificity protein phosphatase 4
MLL: Mixed lineage leukemia
MM: Multiple myeloma
MOMP: Mitochondrial outer membrane permeabilization
MSK1: Mitogen- and stress-activated protein kinase 1
Mst1: Mammalian sterile-20-like kinase 1
mTOR: Mammalian target of rapamycin
c-Myb: Avian myeloblastosis virus oncogene cellular homolog
c-Myc: Avian myelocytomatosis virus oncogene cellular homolog
NGF: Nerve growth factor
NFAT: Nuclear factor of activated T cells
NFκB: Nuclear factor κB
NF-Y: Nuclear transcription factor Y
NK: Natural killer
NOD: Non-obese diabetes
NSCLC: Non-small-cell lung cancer
PAR: Protease-activated receptor
PARP: Poly(ADP-ribose)polymerase
Pdx-1: Pancreatic duodenal homeobox 1
Perk: PKR-like endoplasmic reticulum kinase
PHLPP2: PH domain and leucine-rich repeat protein phosphatase 2
PI3K: Phosphatidylinositol 3-kinase
Pin1: Peptidylprolyl cis/trans isomerase, NIMA-interacting 1
PINCH-1: Particularly interesting cysteine-histidine rich protein-1
PKM2: Pyruvate kinase M2
PML: Promyelocytic leukemia
Pnn: Pinin
POKEMON: POZ and Krüppel (POK) erythroid myeloid ontogenic factor
PP2A: Protein phosphatase 2A
PRC2: Polycomb-repressive complex 2
PTBP1: Polypyrimidine tract binding protein
PTEN: Phosphatase and tensin homolog deleted on chromosome 10
PTPN2: Protein tyrosine phosphatase, non-receptor type 2
Puma: p53-upregulated modulator of apoptosis
RACK1: Receptor for activated C kinase 1
Rb: Retinoblastoma
RBP: RNA binding protein
ROS: Reactive oxygen species
RSK: Ribosomal S6 kinase
Runx: Runt-related transcription factor
SAHA: Suberoylanilide hydroxamic acid
SBHA: Suberoyl bis-hydroxamic acid
SCLC: Small-cell lung cancer
SGK: Serum- and Glucocorticoid-inducible kinase
SHIP1: Src homology-2 domain-containing inositol 5-phosphatase 1
SIRT1: Sirtuin 1
Smac: Second mitochondria-derived activator of caspase
Smad: Contraction of Sma and Mad (Mothers against decapentaplegic)
SNAI2: Snail family zinc finger 2
SFSR6 (SRSF6): Splicing factor arginine/serine (RS)-rich 6
SHP2: SH2 domain-containing phosphatase 2
SphK1: Sphingosine kinase 1
SRSF1 (SFSR1): Serine/arginine-rich splicing factor-1
STAT-1: Signal transducer and activator of transcription 1
SUZ12: Suppressor of zeste 12 homolog
TCR: T cell receptor
TGFβ: Transforming growth factor β
TKI: Tyrosine kinase inhibitor
TLR: Toll-like receptor
TNFα: Tumor necrosis factor α
TRAIL: Tumor necrosis factor-related apoptosis-inducing ligand
β-TrCP1: Beta-transducin repeat containing E3 ubiquitin protein ligase 1
Treg: T regulatory cells
Trim33: Tripartite motif containing 33
Trx-1: Thioredoxin 1
Txnip: Thioredoxin-interacting protein
USP9X: Ubiquitin-Specific Protease 9X
3′-UTR: 3′-Untranslated region
VDAC: Voltage-dependent anion channel
YY-1: Ying Yang 1
XIAP: X-linked inhibitor of apoptosis protein
