# Supplementary material for: Regulation of Bim in Health and Disease
Source: Oncotarget. 2015 Sep 5;6(27):23058–134. doi: 10.18632/oncotarget.5492 (PMC4695108; doi:10.18632/oncotarget.5492)
Supplement: Supplementary file 1 [file oncotarget-06-23058-s001.pdf]

Regulation of Bim in Health and Disease

Supplementary Material

Supplementary Table 1. A Summary of Human Bim isoforms. This table summarizes the Bim isoforms described in human cells and their respective nucleotide and protein accession number. All data has been integrated from Pubmed and Swiss UniProt information and the papers describing the various isoforms [9, 17, 28, 30]. The pro-apoptotic function of Bim depends on the BH3 domain (aa 148-162 of Bim_{EL}) encoded by exon E4. The Bim structures of some of the isoforms are shown in Figure 1.

Isoform	Protein Length	Mass (Dalton)	The structure in comparison to Bim_{EL}
**Bim_{EL}**; Bcl2L11 transcript variant 1; NM_138621.4 NP_619527.1	198 aa	22,171	• This variant encodes the longest and most prominently expressed isoform, and is therefore considered the canonical isoform.<br>• Contains coding exons E2A, E2B, E2C, E4 and E5.<br>• It is pro-apoptotic.
**Bim_{L}**; Bcl2L11 transcript variant 6; NM_006538.4 NP_006529.1	138 aa	15,967	• Lacks aa 42-101 (exon E2B).<br>• Contains coding exons E2A, E2C, E4 and E5.<br>• It is pro-apoptotic.
**Bim_{S}**; Bcl2L11 transcript variant 11; NM_001204106.1 NP_001191035.1	108 aa	12,717	• Lacks aa 42-131 (exons E2B, E2C).<br>• Contains coding exons E2A, E4 and E5.<br>• It is pro-apoptotic.
**Bimα1**; BimABCD; Bcl2L11 transcript variant 2; NM_138622.3 NP_619528.1	169 aa	18,536	• aa 167-198 are exchanged with an alternate intronic region to get: VFLNNYQAAEDHPRMVILRLLRYIVRLVWRMH→LEK<br>• Contains coding exons E2A, E2B, E2C, E4 and an alternative intron region located between exon E4 and E5 giving rise to a distinct C-terminus, thus lacking the hydrophobic C-terminal region.<br>• It is pro-apoptotic.
**Bimα2**; BimACD; Bcl2L11 transcript variant 3; NM_138623.3 NP_619529.1	109 aa	12,332	• Lacks aa 42-101 (exon 2B).<br>• aa 167-198 are exchanged with an alternate intronic region to get: VFLNNYQAAEDHPRMVILRLLRYIVRLVWRMH→LEK<br>• Contains coding exons E2A,E2C, E4 and an alternative intron region located between exon E4 and E5 giving rise to a distinct C-terminus, thus lacking the hydrophobic C-terminal region.<br>• It is pro-apoptotic.
**Bimα3**; BimAD**;** Bcl2L11 transcript variant 10; NM_207003.2 NP_996886.1	79 aa	9,081	• Lacks aa 42-131 (exons E2B and E2C)<br>• aa 167-198 are exchanged with an alternate intronic region to get: VFLNNYQAAEDHPRMVILRLLRYIVRLVWRMH→LEK<br>• Contains coding exons E2A, E4 and an alternative intron region located between exon E4 and E5, giving rise to a distinct C-terminus, thus lacking the hydrophobic C-terminal region.<br>• . It is pro-apoptotic.
**Bimα4**; Bcl2L11 transcript variant 12; NM_001204107.1 NP_001191036.1	85 aa	9,596	• Lacks aa 42-131 (exons E2B and E2C).<br>• aa 167-198 are exchanged with an alternative C terminus: VFLNNYQAAEDHPRMVILRLLRYIVRLVWRMH→LAKLLASST<br>• Contains coding exons E2A, E4 and an alternative intron region located between exon E4 and E5 giving rise to a distinct C-terminus, thus lacking the hydrophobic C-terminal region.<br>It is pro-apoptotic

Supplemementary Table 1 Cont'd

Isoform	Protein Length	Mass (Dalton)	The structure in comparison to Bim_{EL}
**Bimα5;** Bcl2L11 transcript variant 13; NM_001204108.1 NP_001191037.1	172 aa	18,816	• aa 167-198 are exchanged with an alternative C terminus: VFLNNYQAAEDHPRMVILRLLRYIVRLVWRMH→MPLPPD<br>• Contains coding exons E2A, E2B, E2C, E4 and an alternative intron region located between exon E4 and E5 giving rise to a distinct C-terminus, thus lacking the hydrophobic C-terminal region.<br>• It is pro-apoptotic.
**Bimα6;** Bcl2L11 transcript variant 15; NM_001204110.1 NP_001191039.1	82 aa	9,362	• Lacks aa 42-131 (exons E2B and E2C).<br>• aa 167-198 are exchanged with an alternative C terminus: VFLNNYQAAEDHPRMVILRLLRYIVRLVWRMH→MPLPPD<br>• Contains coding exons E2A, E4 and an alternative intron region located between exon E4 and E5 giving rise to a distinct C-terminus, thus lacking the hydrophobic C-terminal region.<br>• It is pro-apoptotic.
**Bimβ1**; Bcl2L11 transcript variant 4; NM_138624.3 NP_619530.1	135 aa	14,458	• aa 133-135 are exchanged: SMR→NWD.<br>• Lacks aa 136-198 (coding exons E4 and E5).<br>• Contains coding exons E2A, E2B, E2C and an alternative intron region located between exon E3 and E4 giving rise to a distinct C-terminus, thus lacking the hydrophobic C-terminal region.<br>• Lacks the pro-apoptotic BH3 region.
**Bimβ2**; Bcl2L11 transcript variant 7; NM_138626.3 NP_619532.1	135 aa	14,418	• aa 132-135 are exchanged: ASMR→GIFE<br>• Lacks aa 136-198 (coding exons E4 and E5)<br>• Contains coding exons E2A, E2B, E2C and a frame shift in E5, thus lacking the hydrophobic C-terminal region.<br>• Lacks the pro-apoptotic BH3 region.
**Bimβ3**; Bcl2L11 transcript variant 5; NM_138625.3 NP_619531.1	75 aa	22,649	• aa 42-75 are exchanged: GNPEGNHGGEGDSCPHGSPQGPLAPPASPGPFAT→VSLCHPG WSALVRSWLTATSNSQVQAVLLPQPPK<br>• Contains coding exons E2A and E3.<br>• Lacks the pro-apoptotic BH3 region.
**Bimβ4**; Bcl2L11 transcript variant 8; NM_138627.3 NP_619533.1	44 aa	4,834	• aa 43-44 are exchanged: NP→IF.<br>• Lacks aa 45-198 (exons E2B, E2C, E4).<br>• Contains coding exons E2A and a frame shift in E5, thus lacking the hydrophobic C-terminal region.<br>• Lacks the pro-apoptotic BH3 region.
**Bimβ5**; Bcl2L11 transcript variant 14; NM_001204109.1 NP_001191038.1	140 aa	15,025	• aa 132-140 are exchanged: ASMRQAEPA→VREIEEVVV.<br>• Lacks aa 141-198 (coding exons E4 and E5), thus lacking the hydrophobic C-terminal region.<br>• It contains exons E2A, E2B and E2C.<br>• Lacks the pro-apoptotic BH3 region.
**Bimβ6**; Bcl2L11 transcript variant 16; NM_001204111.1 NP_001191040.1	75 aa	8,214	• Lacks aa 42-101 (exon E2B)<br>• aa 132-135 are exchanged: ASMR→GIFE.<br>• Lacks aa 136-198 (coding exons E4 and E5), thus lacking the hydrophobic C-terminal region.<br>• Lacks the pro-apoptotic BH3 region.

Supplemementary Table 1 Cont'd

**Isoform**	**Protein Length**	**Mass (Dalton)**	**The structure in comparison to Bim$_{EL}$**
**Bimγ2**; Bcl2L11 transcript variant 9; NM_207002.3 NP_996885.1	112 aa	12,412	• Lacks aa 42-101 (exon E2B)<br>• aa 132-198 are exchanged: ASMRQAEPAD...YIVRLVWRMH→VVILEDIGDL...TEQLNHKDF S<br>• It contains exons E2A and E2C and the alternative E3, leading to premature stop codon, thus lacking E4 and E5 exons.<br>• Lacks the hydrophobic C-terminal region.<br>• Lacks the pro-apoptotic BH3 region.
Bcl2L11 transcript variant 17; NM_001204112.1 NP_001191041.1	80 aa		• It contains E2A and E2C followed by a 29 bp C-terminal coding sequence from the intron insertion region between E4 and E5.
Bcl2L11 transcript variant 18; NM_001204113.1 NP_001191042.1	98 aa		• It contains E2A and E2C followed by an 83 bp C-terminal coding sequence.
**BimAC**	103 aa	11,773	• Lacks aa 42-101 (exon E2B) and aa 132-166 (exon E4).<br>• Contains coding exons E2A, E2B, E2C and a truncated C-terminus expressing GIFE, thus lacking the hydrophobic C-terminal region.<br>• Lacks the pro-apoptotic BH3 region.
**BimABC**	163 aa	17,977	• Lacks aa 132-166 (exon E4).<br>• Contains coding exons E2A, E2B, E2C and a truncated C-terminus expressing GIFE, thus lacking the hydrophobic C-terminal region.<br>• Lacks the pro-apoptotic BH3 region.
**BimA**	73 aa	8,523	• Lacks aa 42-166 (exons E2B, E2C and E4).<br>• Contains coding exons E2A and and a truncated C-terminus expressing GIFE, thus lacking the hydrophobic C-terminal region.<br>• Lacks the pro-apoptotic BH3 region.
